# Supplementary material for: Developing Prediction Models Using Near-Infrared Spectroscopy to Quantify Cannabinoid Content in Cannabis Sativa
Source: Sensors (Basel). 2023 Feb 27;23(5):2607. doi: 10.3390/s23052607 (PMC10007171; doi:10.3390/s23052607)
Supplement: Supplementary file 1 [file sensors-23-02607-s001.zip › sensors-2200076-supplementary/Figure S1 Histogram plot of Cannbinoids by Harvest Group.pdf]

Figure S1: Histogram plots of all cannabinoids by harvest group (HG1: n = 479, HG2: n = 126, HG 3: n = 78, HG4: n = 51).

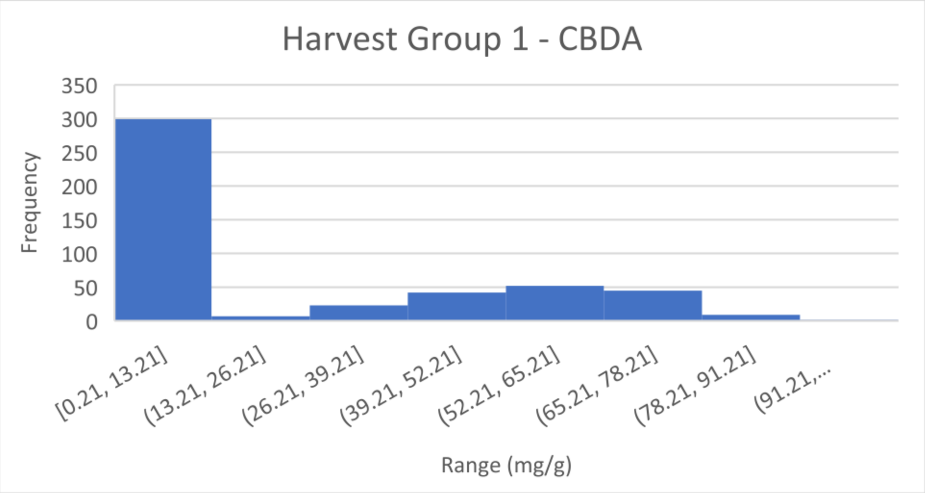

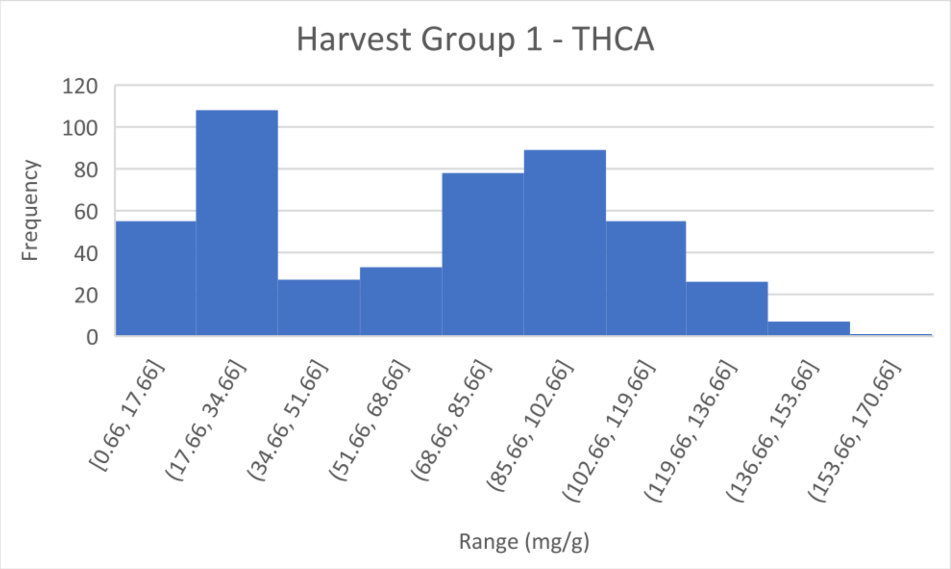

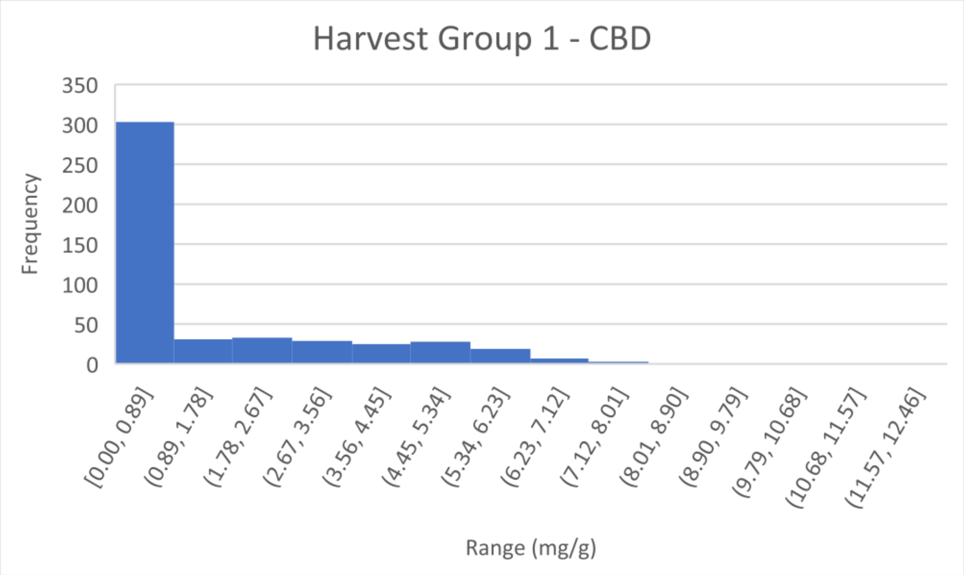
Harvest Group 1 - CBD
Frequency
350
300
250
200
150
100
50
0
[0.00, 0.89]
(0.89, 1.78]
(1.78, 2.67]
(2.67, 3.56]
(3.56, 4.45]
(4.45, 5.34]
(5.34, 6.23]
(6.23, 7.12]
(7.12, 8.01]
(8.01, 8.90]
(8.90, 9.79]
(9.79, 10.68]
(10.68, 11.57]
(11.57, 12.46]
Range (mg/g)

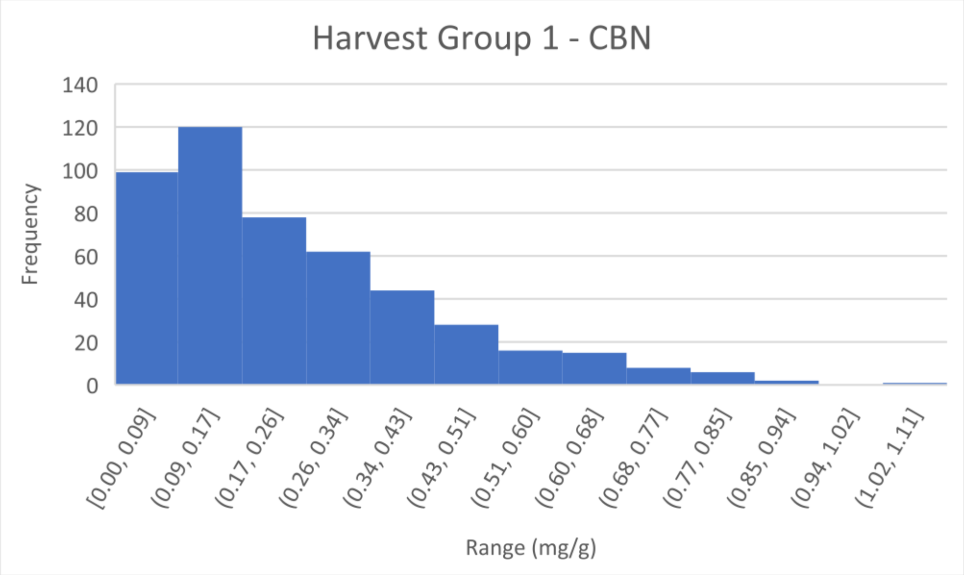
Harvest Group 1 - CBN
Frequency
140
120
100
80
60
40
20
0
[0.00, 0.09]
(0.09, 0.17]
(0.17, 0.26]
(0.26, 0.34]
(0.34, 0.43]
(0.43, 0.51]
(0.51, 0.60]
(0.60, 0.68]
(0.68, 0.77]
(0.77, 0.85]
(0.85, 0.94]
(0.94, 1.02]
(1.02, 1.11]
Range (mg/g)

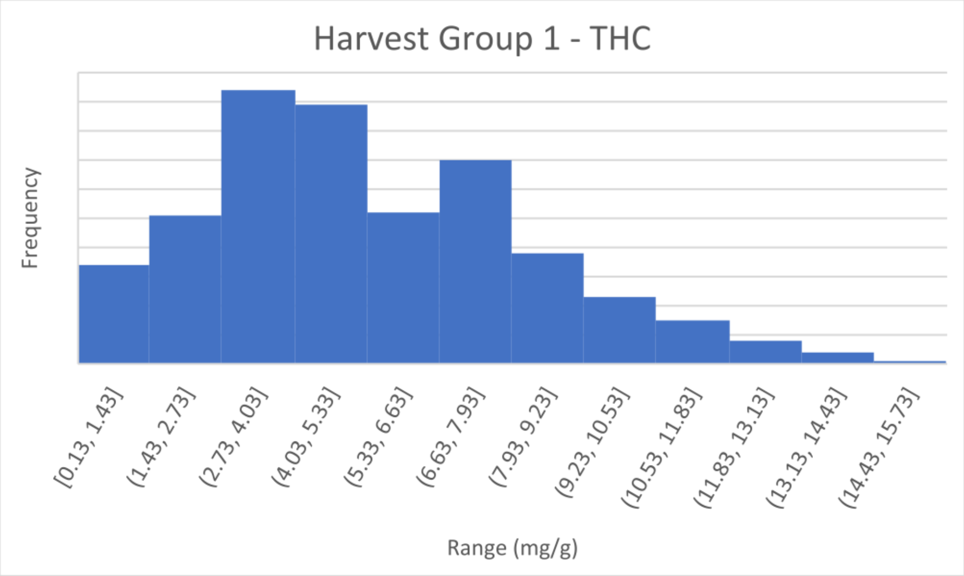
Harvest Group 1 - THC
Frequency
[0.13, 1.43]
(1.43, 2.73]
(2.73, 4.03]
(4.03, 5.33]
(5.33, 6.63]
(6.63, 7.93]
(7.93, 9.23]
(9.23, 10.53]
(10.53, 11.83]
(11.83, 13.13]
(13.13, 14.43]
(14.43, 15.73]
Range (mg/g)

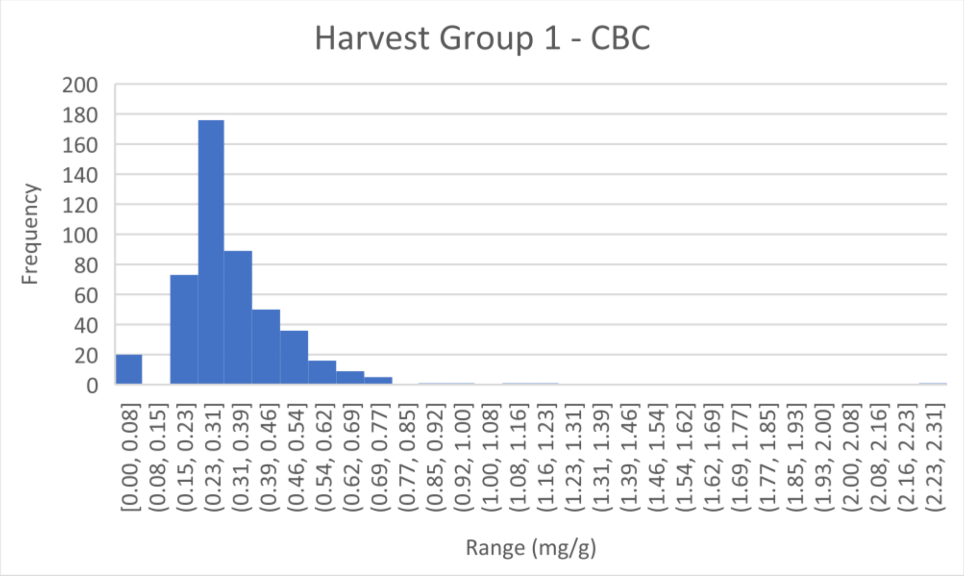
Harvest Group 1 - CBC
Frequency
200
180
160
140
120
100
80
60
40
20
0
[0.00, 0.08]
(0.08, 0.15]
(0.15, 0.23]
(0.23, 0.31]
(0.31, 0.39]
(0.39, 0.46]
(0.46, 0.54]
(0.54, 0.62]
(0.62, 0.69]
(0.69, 0.77]
(0.77, 0.85]
(0.85, 0.92]
(0.92, 1.00]
(1.00, 1.08]
(1.08, 1.16]
(1.16, 1.23]
(1.23, 1.31]
(1.31, 1.39]
(1.39, 1.46]
(1.46, 1.54]
(1.54, 1.62]
(1.62, 1.69]
(1.69, 1.77]
(1.77, 1.85]
(1.85, 1.93]
(1.93, 2.00]
(2.00, 2.08]
(2.08, 2.16]
(2.16, 2.23]
(2.23, 2.31]
Range (mg/g)

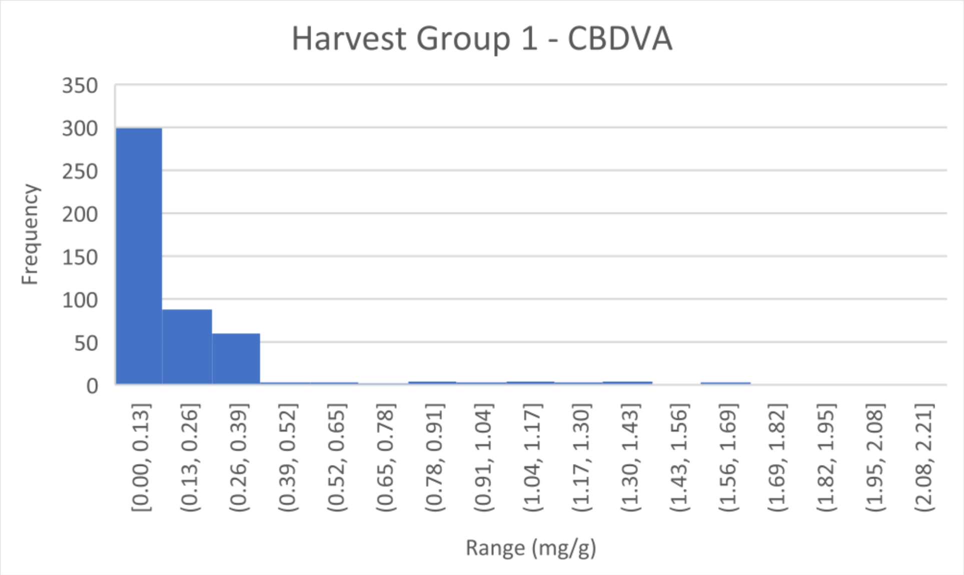
Harvest Group 1 - CBDVA
Frequency
350
300
250
200
150
100
50
0
[0.00, 0.13]
(0.13, 0.26]
(0.26, 0.39]
(0.39, 0.52]
(0.52, 0.65]
(0.65, 0.78]
(0.78, 0.91]
(0.91, 1.04]
(1.04, 1.17]
(1.17, 1.30]
(1.30, 1.43]
(1.43, 1.56]
(1.56, 1.69]
(1.69, 1.82]
(1.82, 1.95]
(1.95, 2.08]
(2.08, 2.21]
Range (mg/g)

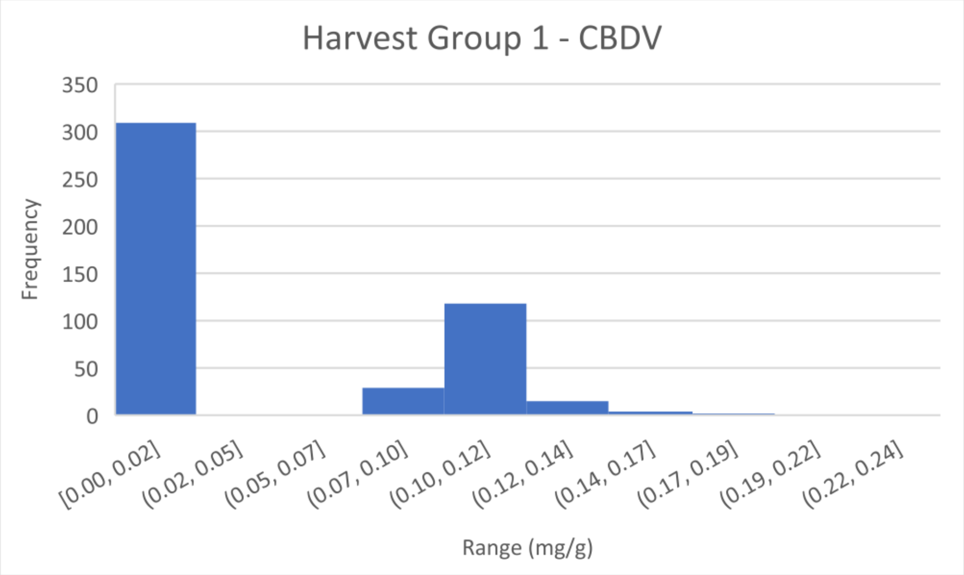
Harvest Group 1 - CBDV
Frequency
350
300
250
200
150
100
50
0
[0.00, 0.02]
(0.02, 0.05]
(0.05, 0.07]
(0.07, 0.10]
(0.10, 0.12]
(0.12, 0.14]
(0.14, 0.17]
(0.17, 0.19]
(0.19, 0.22]
(0.22, 0.24]
Range (mg/g)

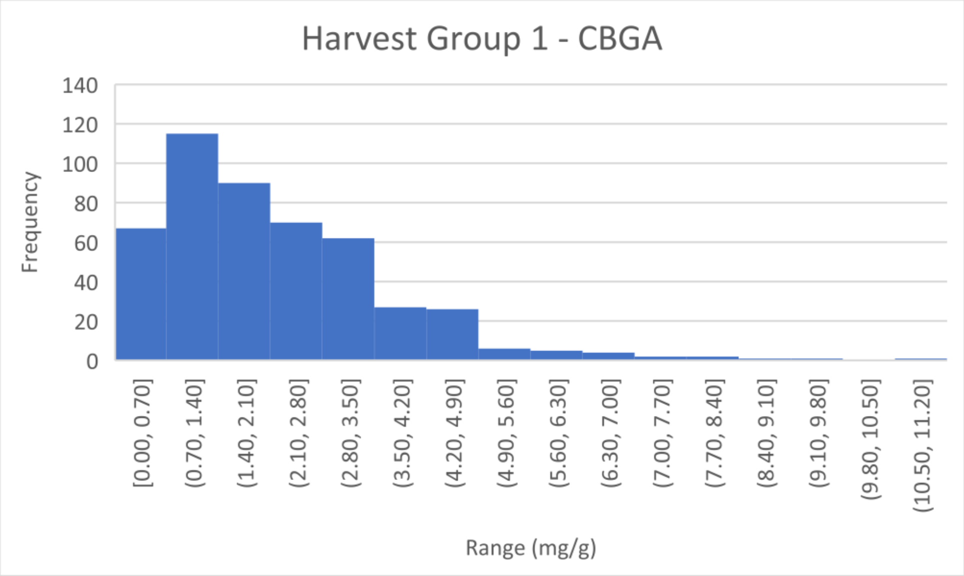
Harvest Group 1 - CBGA
Frequency
140
120
100
80
60
40
20
0
[0.00, 0.70]
(0.70, 1.40]
(1.40, 2.10]
(2.10, 2.80]
(2.80, 3.50]
(3.50, 4.20]
(4.20, 4.90]
(4.90, 5.60]
(5.60, 6.30]
(6.30, 7.00]
(7.00, 7.70]
(7.70, 8.40]
(8.40, 9.10]
(9.10, 9.80]
(9.80, 10.50]
(10.50, 11.20]
Range (mg/g)

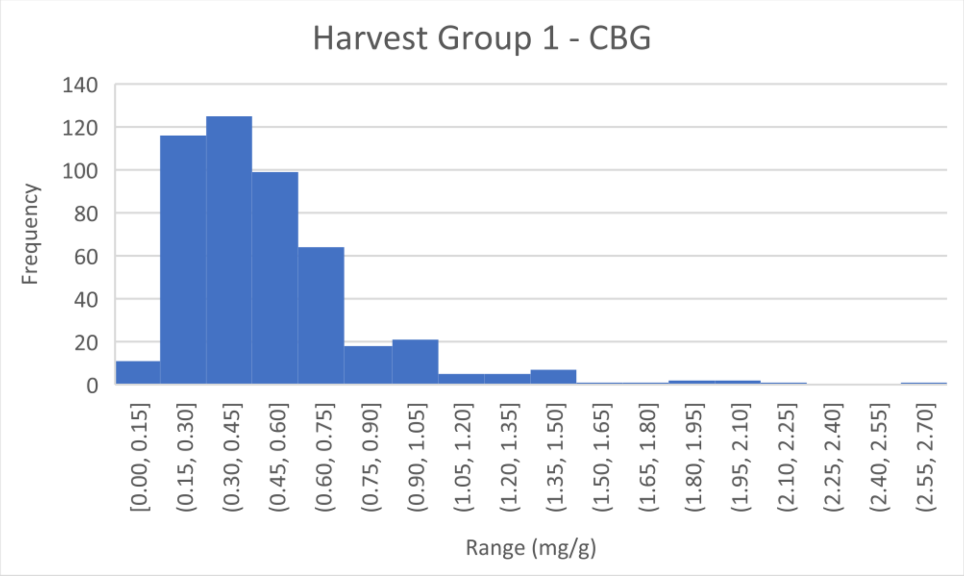
Harvest Group 1 - CBG
Frequency
140
120
100
80
60
40
20
0
[0.00, 0.15]
(0.15, 0.30]
(0.30, 0.45]
(0.45, 0.60]
(0.60, 0.75]
(0.75, 0.90]
(0.90, 1.05]
(1.05, 1.20]
(1.20, 1.35]
(1.35, 1.50]
(1.50, 1.65]
(1.65, 1.80]
(1.80, 1.95]
(1.95, 2.10]
(2.10, 2.25]
(2.25, 2.40]
(2.40, 2.55]
(2.55, 2.70]
Range (mg/g)

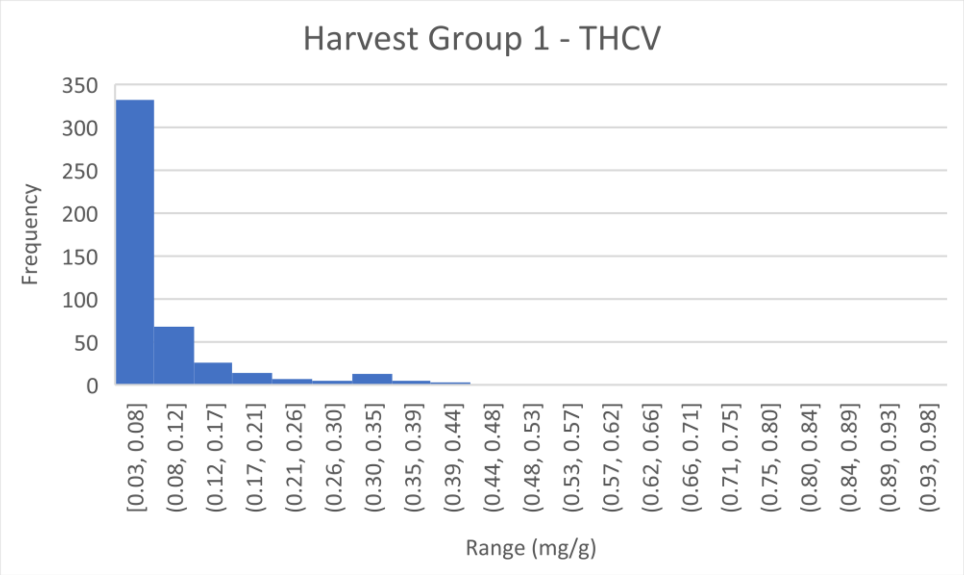
Harvest Group 1 - THCV
Frequency
350
300
250
200
150
100
50
0
[0.03, 0.08]
(0.08, 0.12]
(0.12, 0.17]
(0.17, 0.21]
(0.21, 0.26]
(0.26, 0.30]
(0.30, 0.35]
(0.35, 0.39]
(0.39, 0.44]
(0.44, 0.48]
(0.48, 0.53]
(0.53, 0.57]
(0.57, 0.62]
(0.62, 0.66]
(0.66, 0.71]
(0.71, 0.75]
(0.75, 0.80]
(0.80, 0.84]
(0.84, 0.89]
(0.89, 0.93]
(0.93, 0.98]
Range (mg/g)

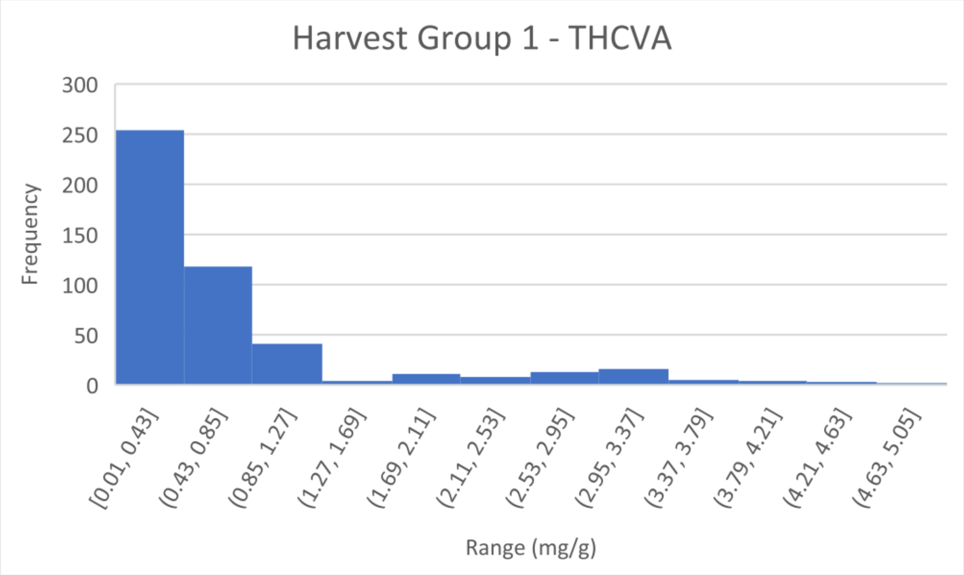
Harvest Group 1 - THCVA
Frequency
300
250
200
150
100
50
0
[0.01, 0.43]
(0.43, 0.85]
(0.85, 1.27]
(1.27, 1.69]
(1.69, 2.11]
(2.11, 2.53]
(2.53, 2.95]
(2.95, 3.37]
(3.37, 3.79]
(3.79, 4.21]
(4.21, 4.63]
(4.63, 5.05]
Range (mg/g)

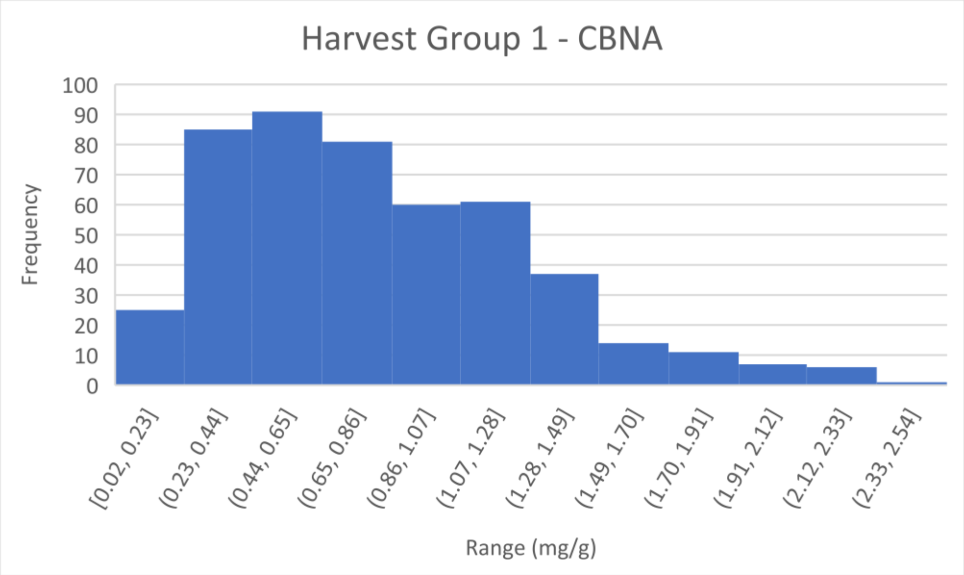
Harvest Group 1 - CBNA
Frequency
100
90
80
70
60
50
40
30
20
10
0
[0.02, 0.23]
(0.23, 0.44]
(0.44, 0.65]
(0.65, 0.86]
(0.86, 1.07]
(1.07, 1.28]
(1.28, 1.49]
(1.49, 1.70]
(1.70, 1.91]
(1.91, 2.12]
(2.12, 2.33]
(2.33, 2.54]
Range (mg/g)

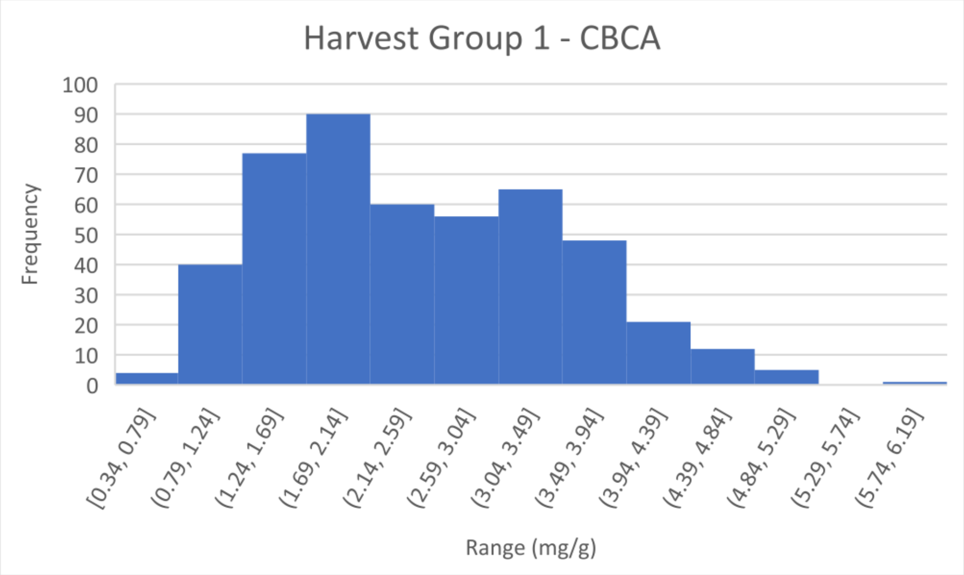
Harvest Group 1 - CBCA
Frequency
100
90
80
70
60
50
40
30
20
10
0
[0.34, 0.79]
(0.79, 1.24]
(1.24, 1.69]
(1.69, 2.14]
(2.14, 2.59]
(2.59, 3.04]
(3.04, 3.49]
(3.49, 3.94]
(3.94, 4.39]
(4.39, 4.84]
(4.84, 5.29]
(5.29, 5.74]
(5.74, 6.19]
Range (mg/g)

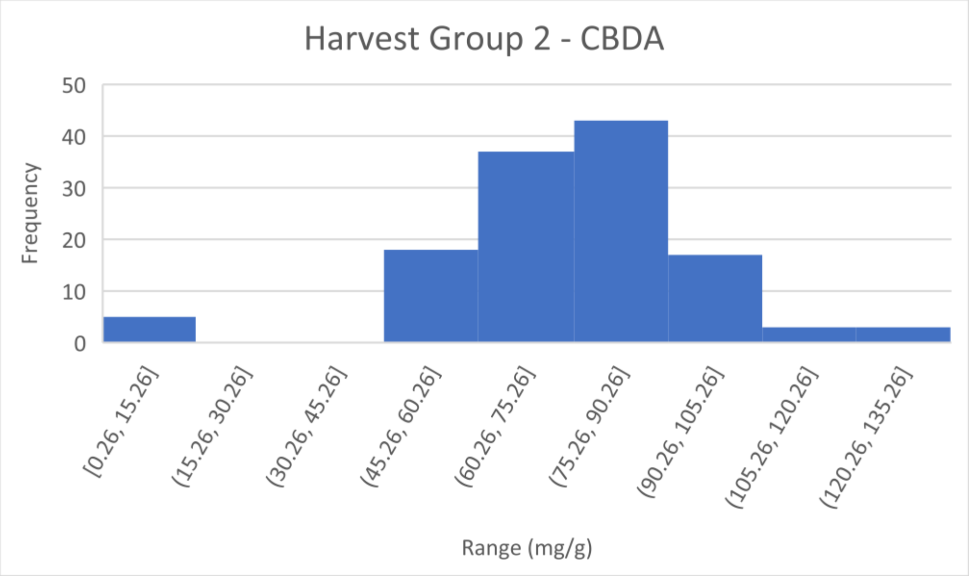
Harvest Group 2 - CBDA
Frequency
0
10
20
30
40
50
[0.26, 15.26]
(15.26, 30.26]
(30.26, 45.26]
(45.26, 60.26]
(60.26, 75.26]
(75.26, 90.26]
(90.26, 105.26]
(105.26, 120.26]
(120.26, 135.26]
Range (mg/g)

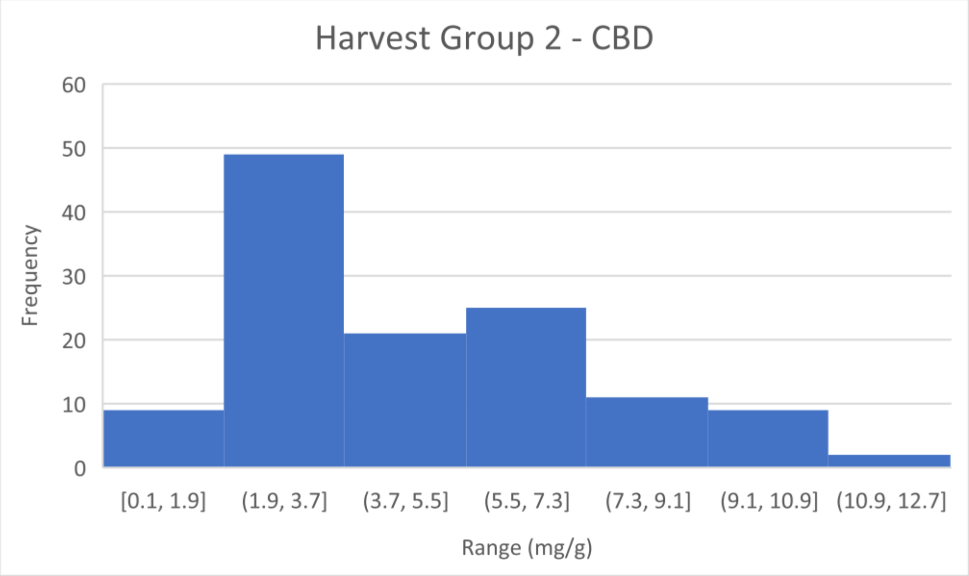
Harvest Group 2 - CBD
Frequency
0
10
20
30
40
50
60
[0.1, 1.9]
(1.9, 3.7]
(3.7, 5.5]
(5.5, 7.3]
(7.3, 9.1]
(9.1, 10.9]
(10.9, 12.7]
Range (mg/g)

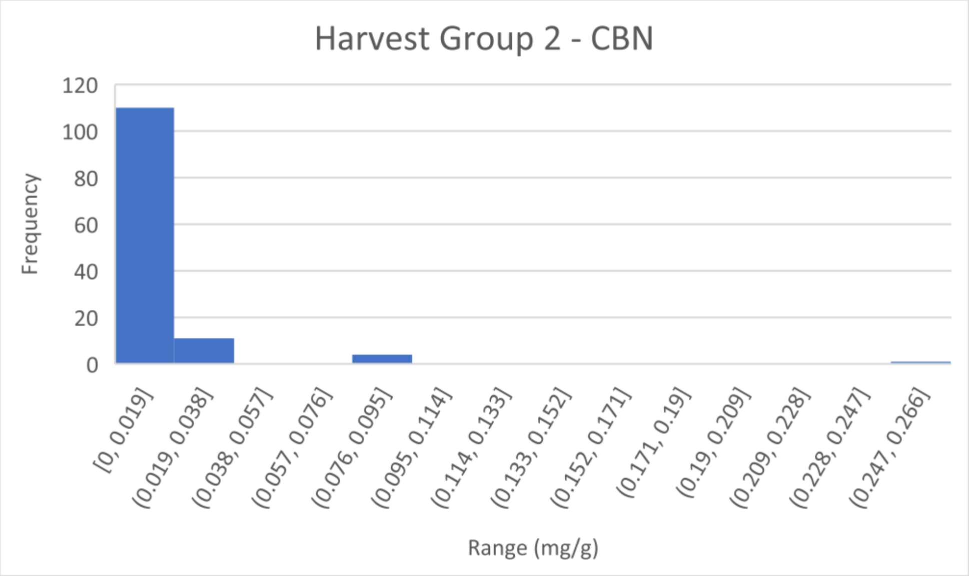
Harvest Group 2 - CBN
120
100
80
60
40
20
0
Frequency
[0, 0.019]
(0.019, 0.038]
(0.038, 0.057]
(0.057, 0.076]
(0.076, 0.095]
(0.095, 0.114]
(0.114, 0.133]
(0.133, 0.152]
(0.152, 0.171]
(0.171, 0.19]
(0.19, 0.209]
(0.209, 0.228]
(0.228, 0.247]
(0.247, 0.266]
Range (mg/g)

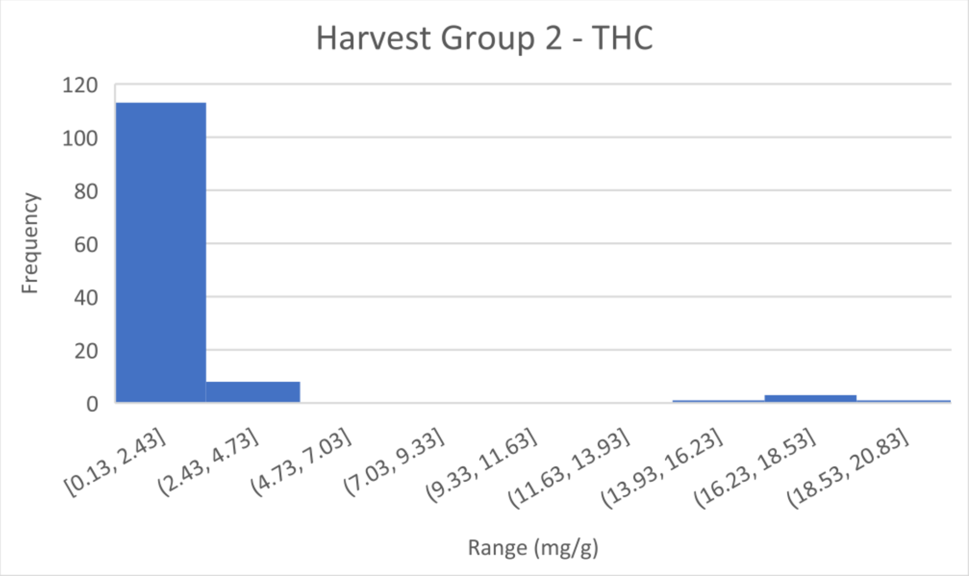
Harvest Group 2 - THC
120
100
80
60
40
20
0
Frequency
[0.13, 2.43]
(2.43, 4.73]
(4.73, 7.03]
(7.03, 9.33]
(9.33, 11.63]
(11.63, 13.93]
(13.93, 16.23]
(16.23, 18.53]
(18.53, 20.83]
Range (mg/g)

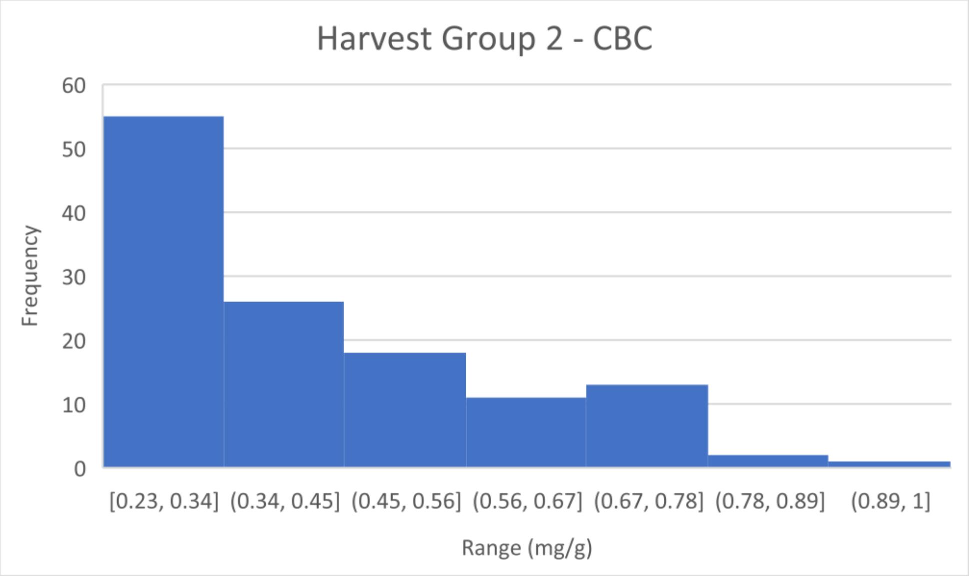
Harvest Group 2 - CBC
Frequency
60
50
40
30
20
10
0
[0.23, 0.34]
(0.34, 0.45]
(0.45, 0.56]
(0.56, 0.67]
(0.67, 0.78]
(0.78, 0.89]
(0.89, 1]
Range (mg/g)

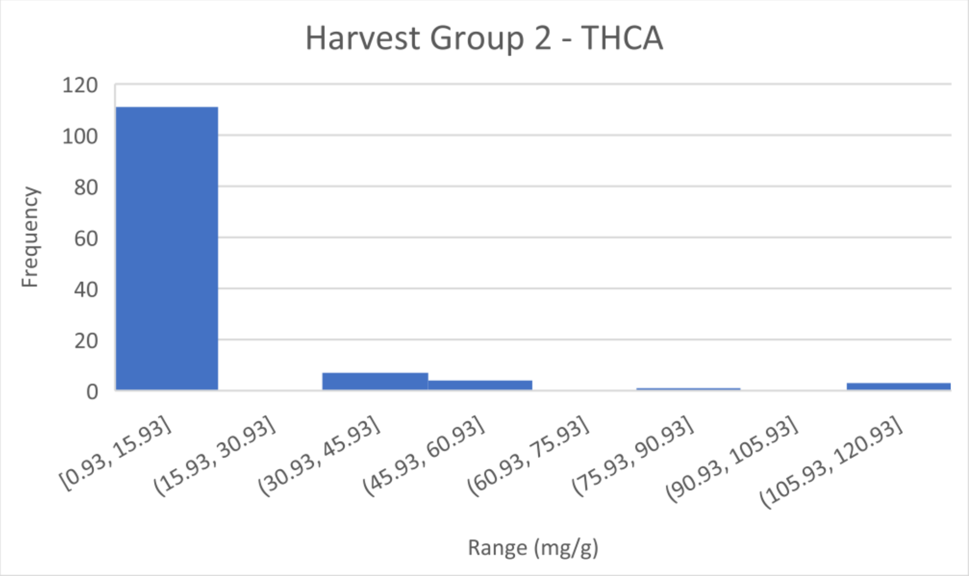
Harvest Group 2 - THCA
Frequency
120
100
80
60
40
20
0
[0.93, 15.93]
(15.93, 30.93]
(30.93, 45.93]
(45.93, 60.93]
(60.93, 75.93]
(75.93, 90.93]
(90.93, 105.93]
(105.93, 120.93]
Range (mg/g)

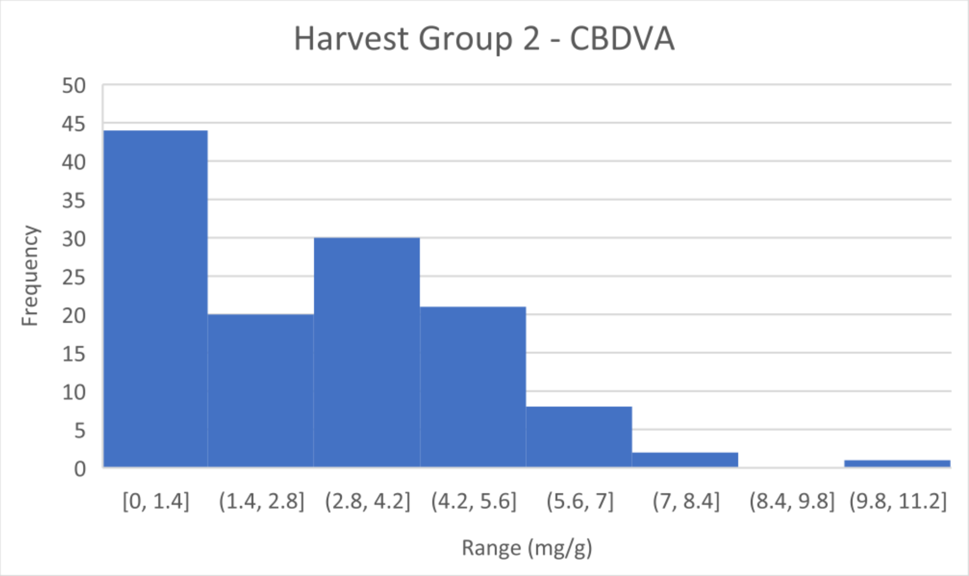
Harvest Group 2 - CBDVA
Frequency
50
45
40
35
30
25
20
15
10
5
0
[0, 1.4]
(1.4, 2.8]
(2.8, 4.2]
(4.2, 5.6]
(5.6, 7]
(7, 8.4]
(8.4, 9.8]
(9.8, 11.2]
Range (mg/g)

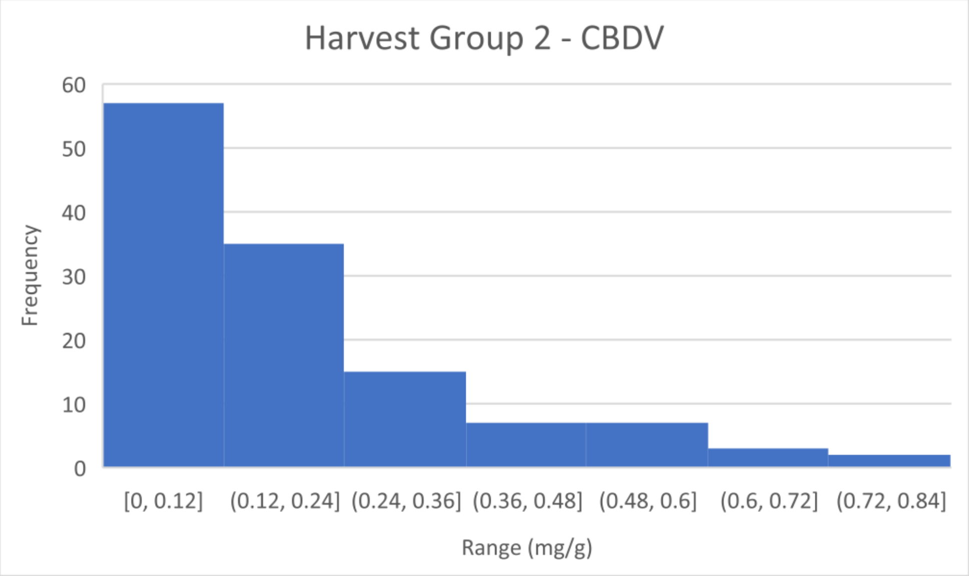
Harvest Group 2 - CBDV
Frequency
60
50
40
30
20
10
0
[0, 0.12]
(0.12, 0.24]
(0.24, 0.36]
(0.36, 0.48]
(0.48, 0.6]
(0.6, 0.72]
(0.72, 0.84]
Range (mg/g)

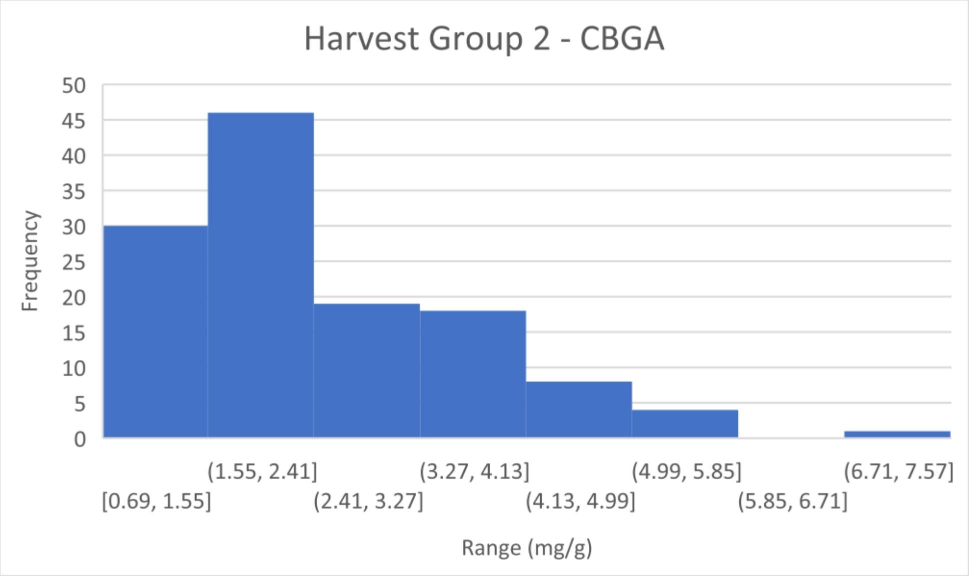
Harvest Group 2 - CBGA
Frequency
50
45
40
35
30
25
20
15
10
5
0
[0.69, 1.55]
(1.55, 2.41]
(2.41, 3.27]
(3.27, 4.13]
(4.13, 4.99]
(4.99, 5.85]
(5.85, 6.71]
(6.71, 7.57]
Range (mg/g)

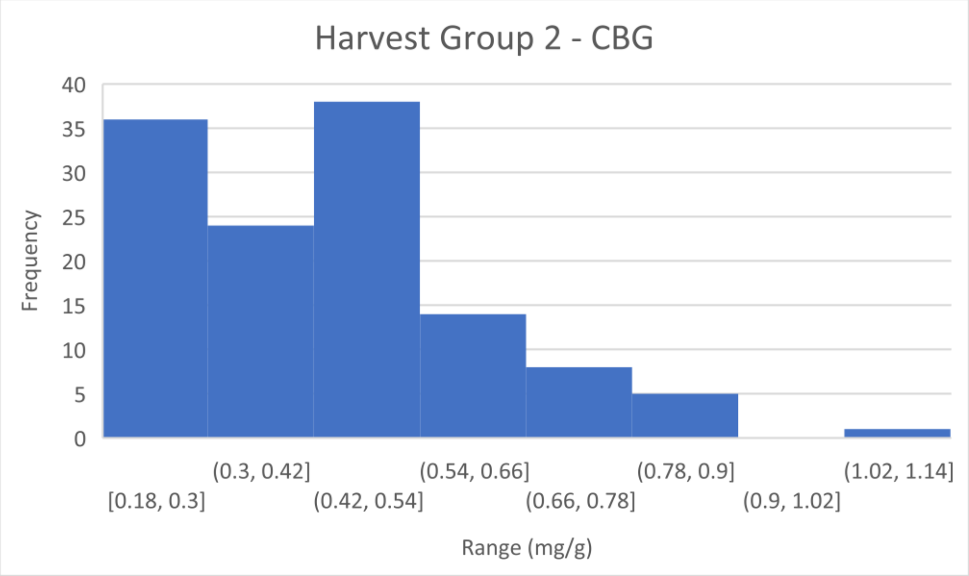
Harvest Group 2 - CBG
Frequency
40
35
30
25
20
15
10
5
0
[0.18, 0.3]
(0.3, 0.42]
(0.42, 0.54]
(0.54, 0.66]
(0.66, 0.78]
(0.78, 0.9]
(0.9, 1.02]
(1.02, 1.14]
Range (mg/g)

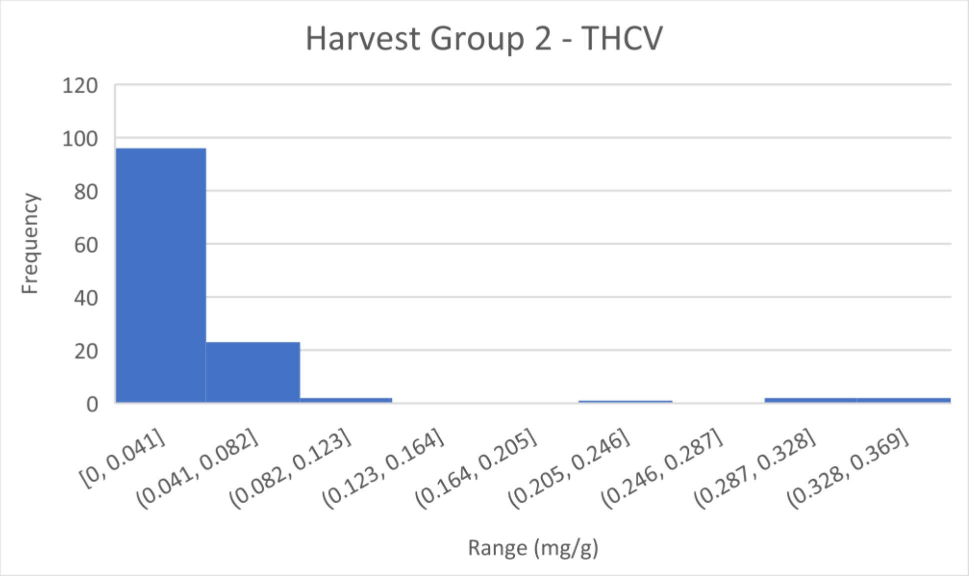

Harvest Group 2 - THCV
120
100
80
60
40
20
0
Frequency
[0, 0.041]
(0.041, 0.082]
(0.082, 0.123]
(0.123, 0.164]
(0.164, 0.205]
(0.205, 0.246]
(0.246, 0.287]
(0.287, 0.328]
(0.328, 0.369]
Range (mg/g)

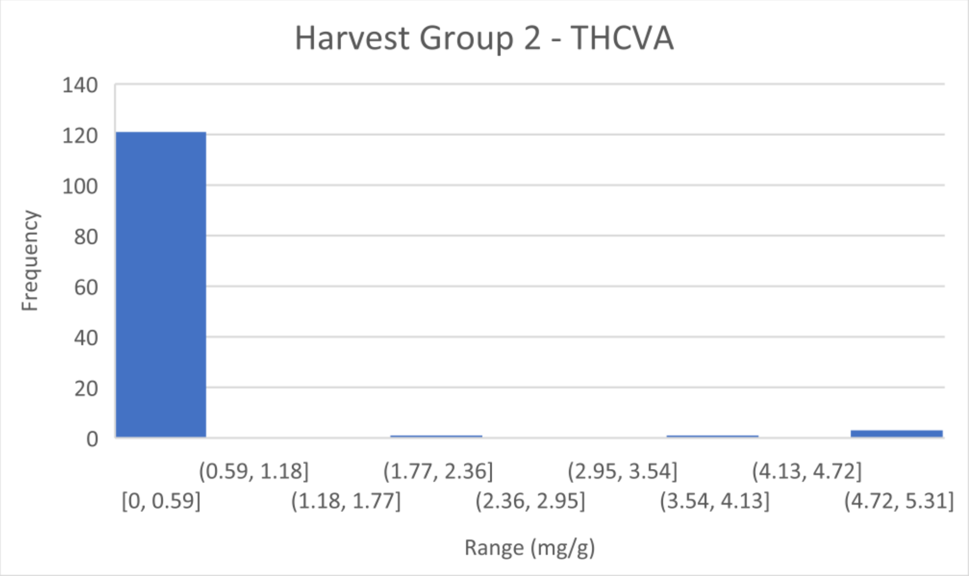

Harvest Group 2 - THCVA
140
120
100
80
60
40
20
0
Frequency
[0, 0.59]
(0.59, 1.18]
(1.18, 1.77]
(1.77, 2.36]
(2.36, 2.95]
(2.95, 3.54]
(3.54, 4.13]
(4.13, 4.72]
(4.72, 5.31]
Range (mg/g)

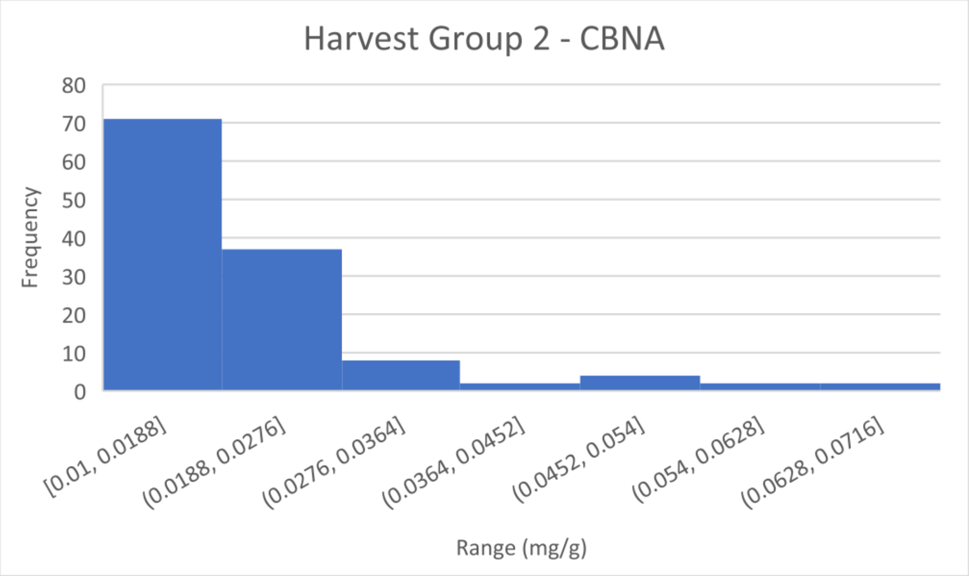
Harvest Group 2 - CBNA
Frequency
0
10
20
30
40
50
60
70
80
[0.01, 0.0188]
(0.0188, 0.0276]
(0.0276, 0.0364]
(0.0364, 0.0452]
(0.0452, 0.054]
(0.054, 0.0628]
(0.0628, 0.0716]
Range (mg/g)

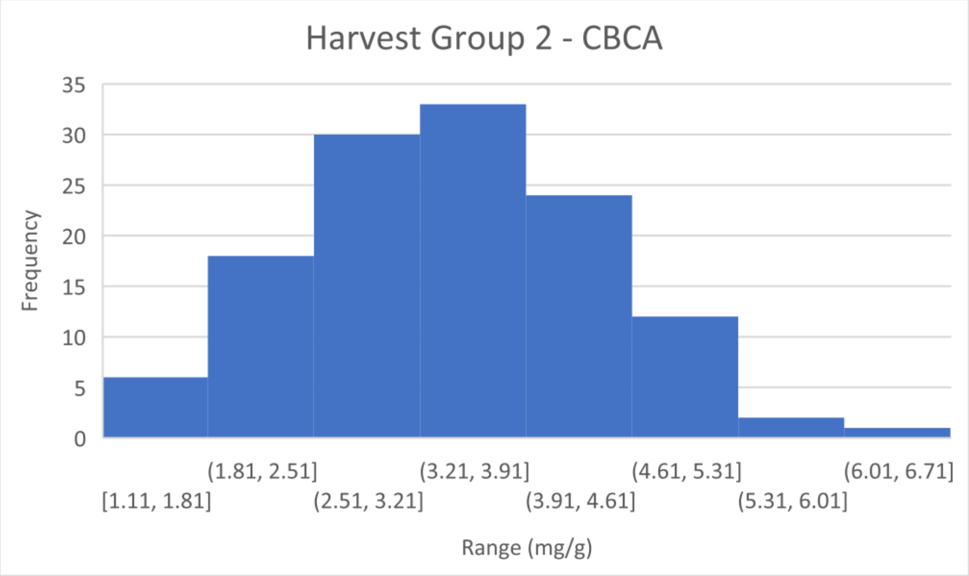
Harvest Group 2 - CBCA
Frequency
0
5
10
15
20
25
30
35
[1.11, 1.81]
(1.81, 2.51]
(2.51, 3.21]
(3.21, 3.91]
(3.91, 4.61]
(4.61, 5.31]
(5.31, 6.01]
(6.01, 6.71]
Range (mg/g)

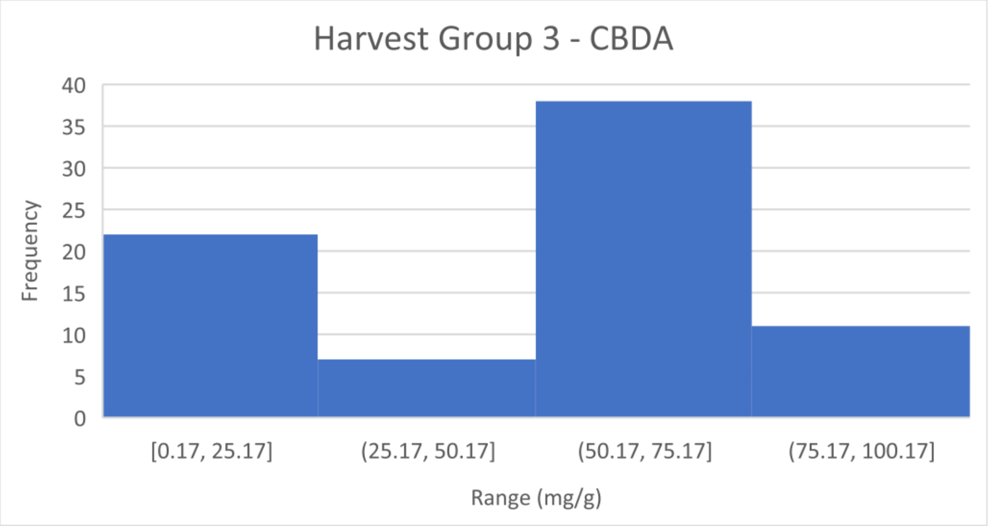
Harvest Group 3 - CBDA
Frequency
40
35
30
25
20
15
10
5
0
[0.17, 25.17]
(25.17, 50.17]
(50.17, 75.17]
(75.17, 100.17]
Range (mg/g)

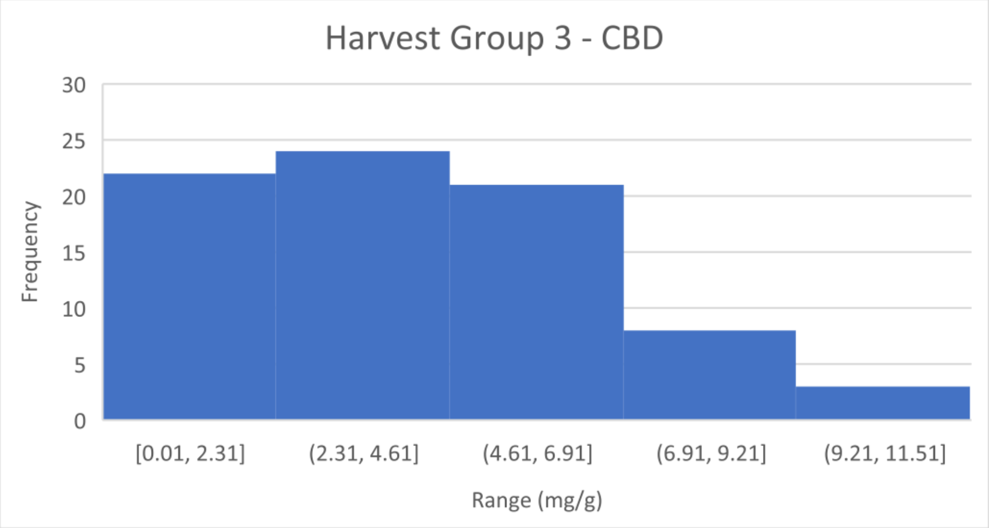
Harvest Group 3 - CBD
Frequency
30
25
20
15
10
5
0
[0.01, 2.31]
(2.31, 4.61]
(4.61, 6.91]
(6.91, 9.21]
(9.21, 11.51]
Range (mg/g)

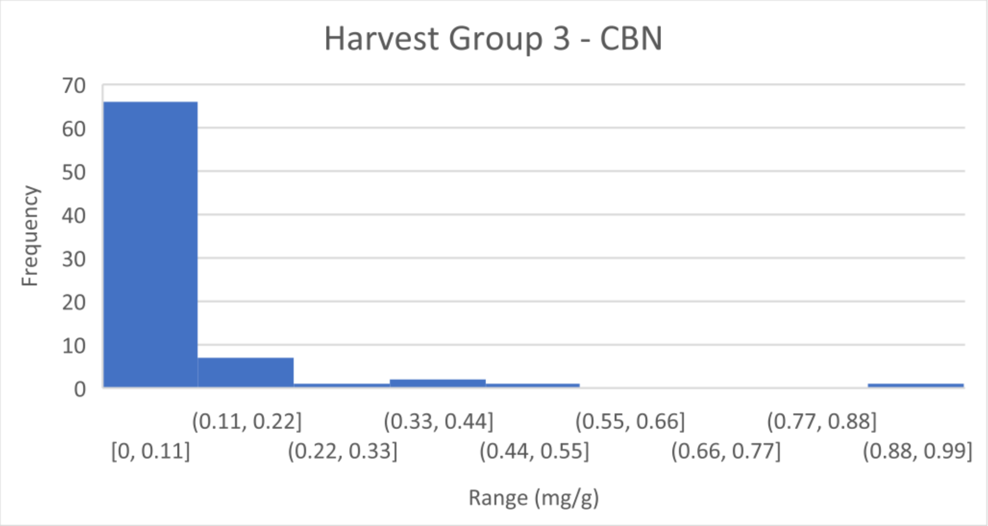
Harvest Group 3 - CBN
Frequency
70
60
50
40
30
20
10
0
[0, 0.11]
(0.11, 0.22]
(0.22, 0.33]
(0.33, 0.44]
(0.44, 0.55]
(0.55, 0.66]
(0.66, 0.77]
(0.77, 0.88]
(0.88, 0.99]
Range (mg/g)

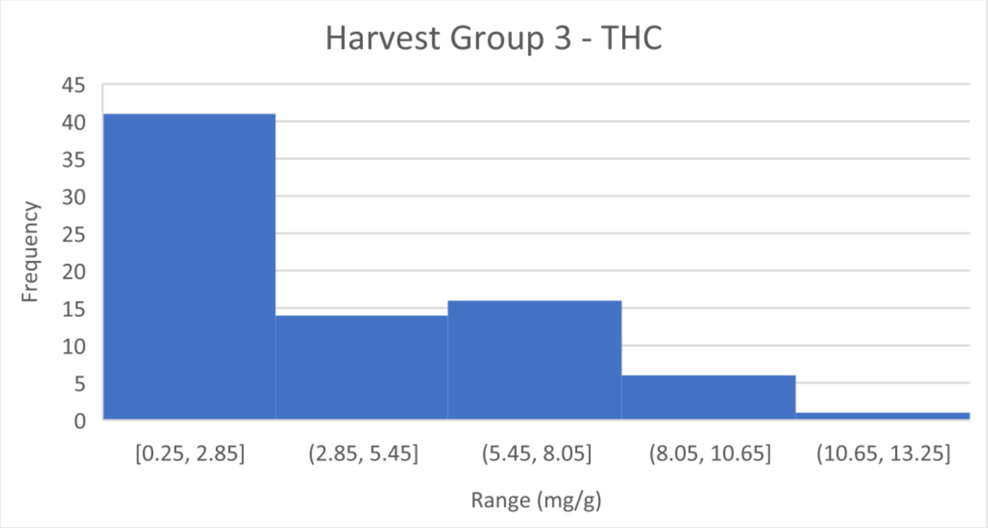
Harvest Group 3 - THC
Frequency
45
40
35
30
25
20
15
10
5
0
[0.25, 2.85]
(2.85, 5.45]
(5.45, 8.05]
(8.05, 10.65]
(10.65, 13.25]
Range (mg/g)

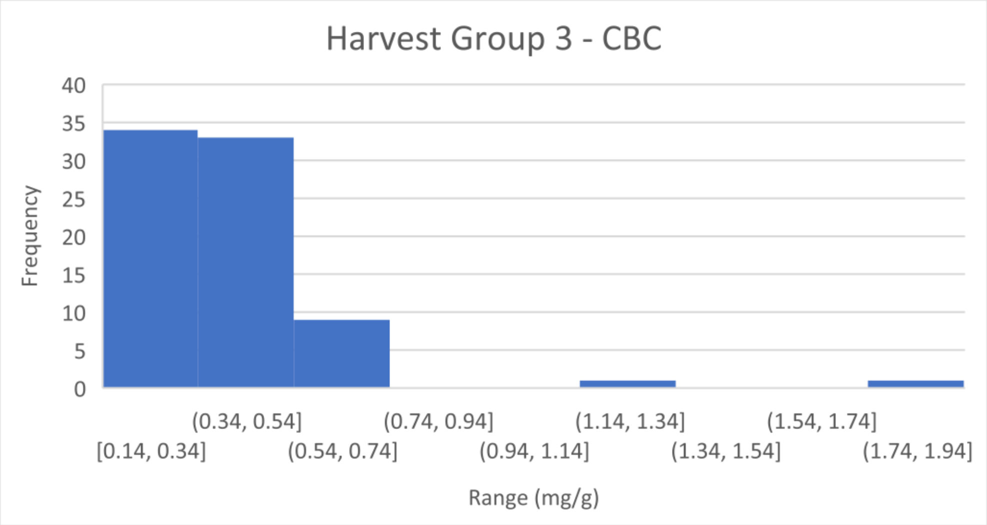
Harvest Group 3 - CBC
Frequency
40
35
30
25
20
15
10
5
0
[0.14, 0.34]
(0.34, 0.54]
(0.54, 0.74]
(0.74, 0.94]
(0.94, 1.14]
(1.14, 1.34]
(1.34, 1.54]
(1.54, 1.74]
(1.74, 1.94]
Range (mg/g)

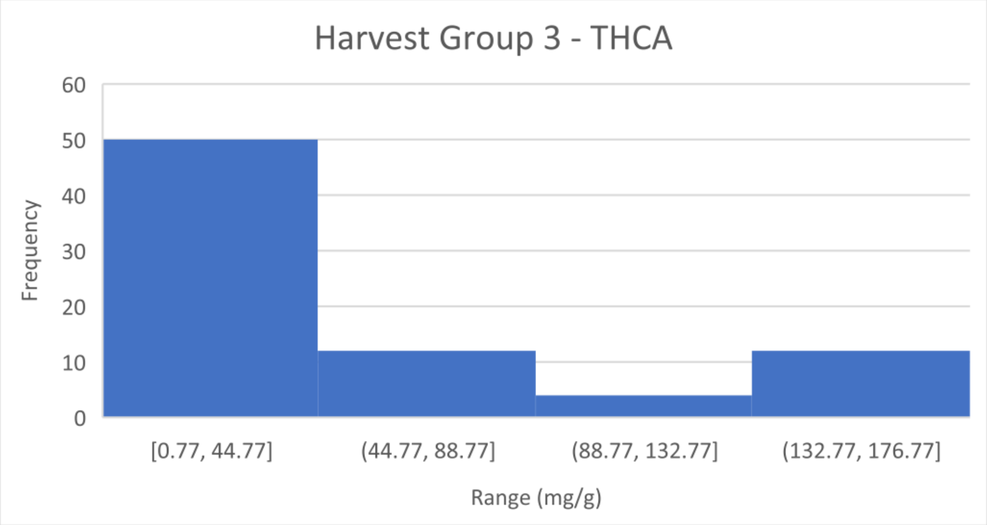
Harvest Group 3 - THCA
Frequency
60
50
40
30
20
10
0
[0.77, 44.77]
(44.77, 88.77]
(88.77, 132.77]
(132.77, 176.77]
Range (mg/g)

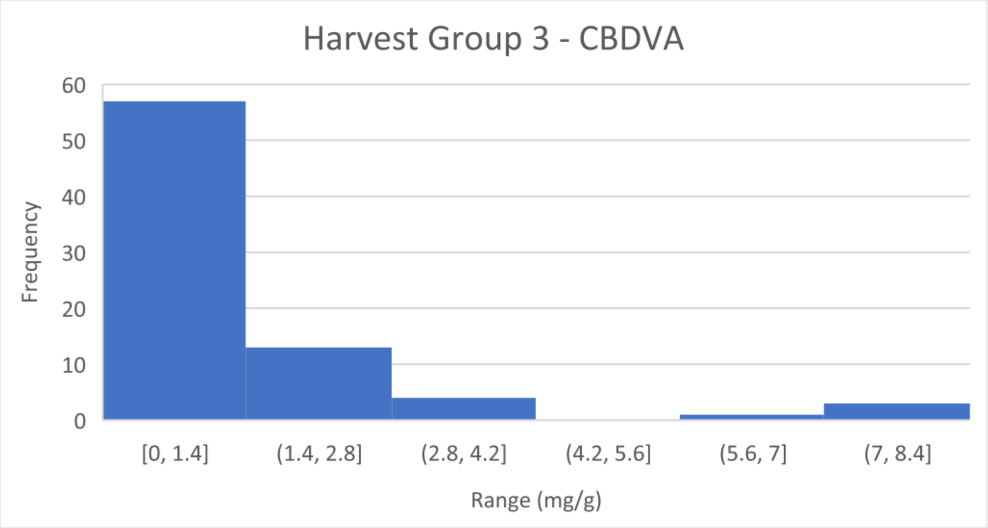
Harvest Group 3 - CBDVA
Frequency
60
50
40
30
20
10
0
[0, 1.4]
(1.4, 2.8]
(2.8, 4.2]
(4.2, 5.6]
(5.6, 7]
(7, 8.4]
Range (mg/g)

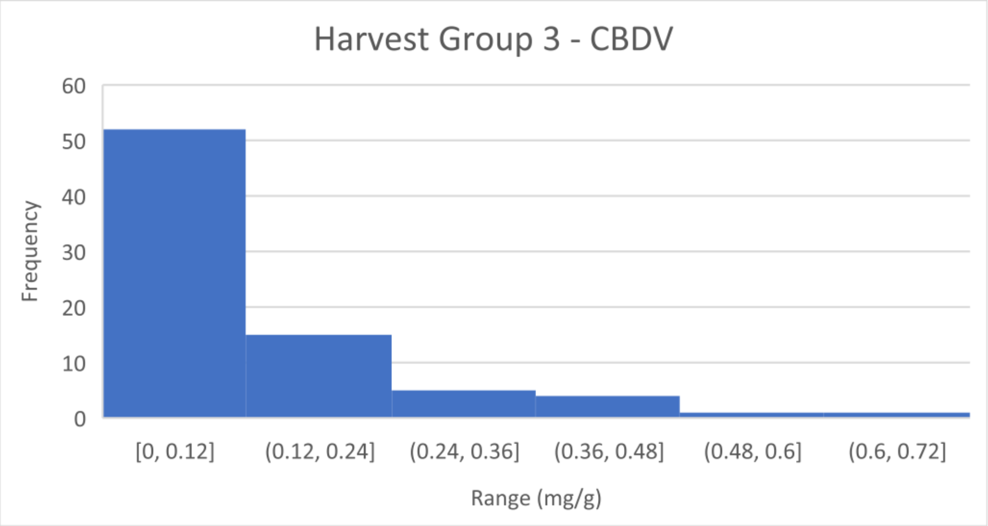
Harvest Group 3 - CBDV
Frequency
60
50
40
30
20
10
0
[0, 0.12]
(0.12, 0.24]
(0.24, 0.36]
(0.36, 0.48]
(0.48, 0.6]
(0.6, 0.72]
Range (mg/g)

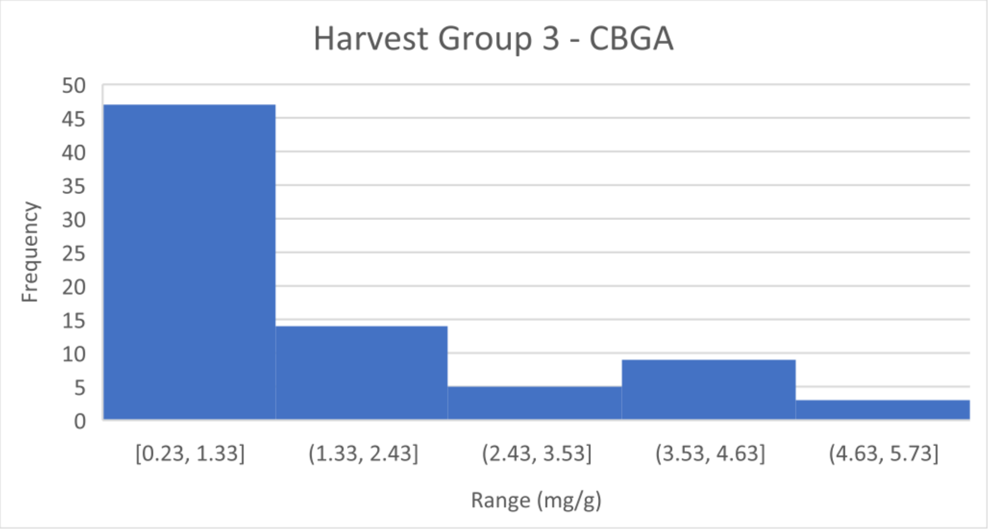
Harvest Group 3 - CBGA
Frequency
50
45
40
35
30
25
20
15
10
5
0
[0.23, 1.33]
(1.33, 2.43]
(2.43, 3.53]
(3.53, 4.63]
(4.63, 5.73]
Range (mg/g)

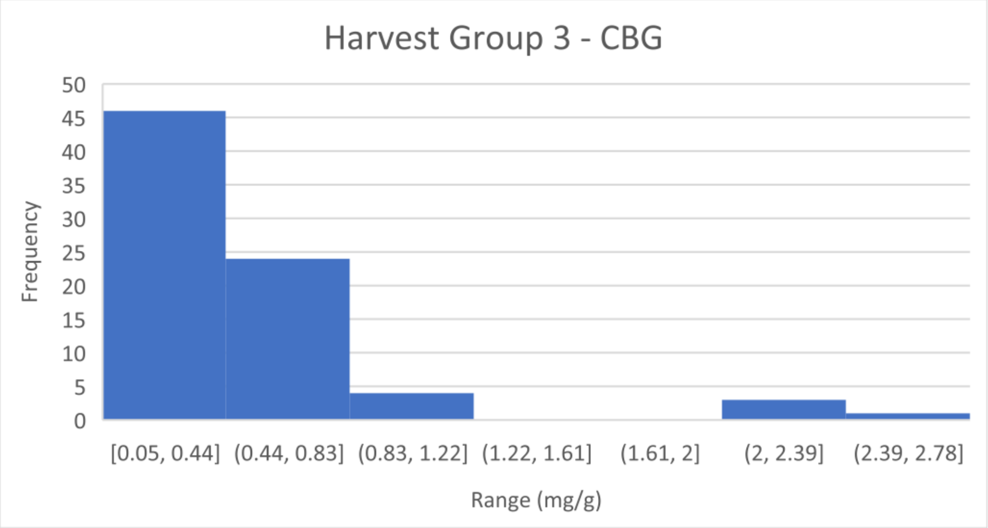
Harvest Group 3 - CBG
Frequency
50
45
40
35
30
25
20
15
10
5
0
[0.05, 0.44]
(0.44, 0.83]
(0.83, 1.22]
(1.22, 1.61]
(1.61, 2]
(2, 2.39]
(2.39, 2.78]
Range (mg/g)

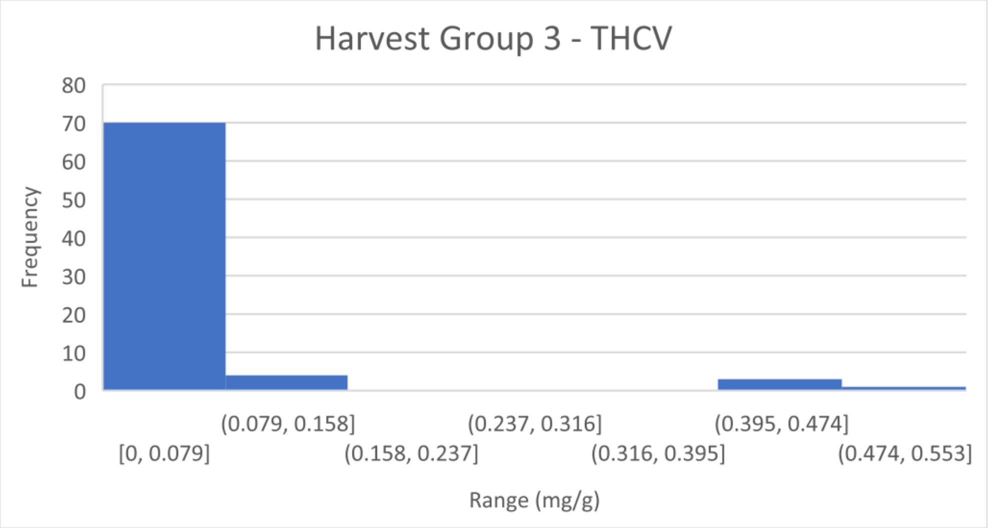
Harvest Group 3 - THCV
80
70
60
50
40
30
20
10
0
Frequency
[0, 0.079]
(0.079, 0.158]
(0.158, 0.237]
(0.237, 0.316]
(0.316, 0.395]
(0.395, 0.474]
(0.474, 0.553]
Range (mg/g)

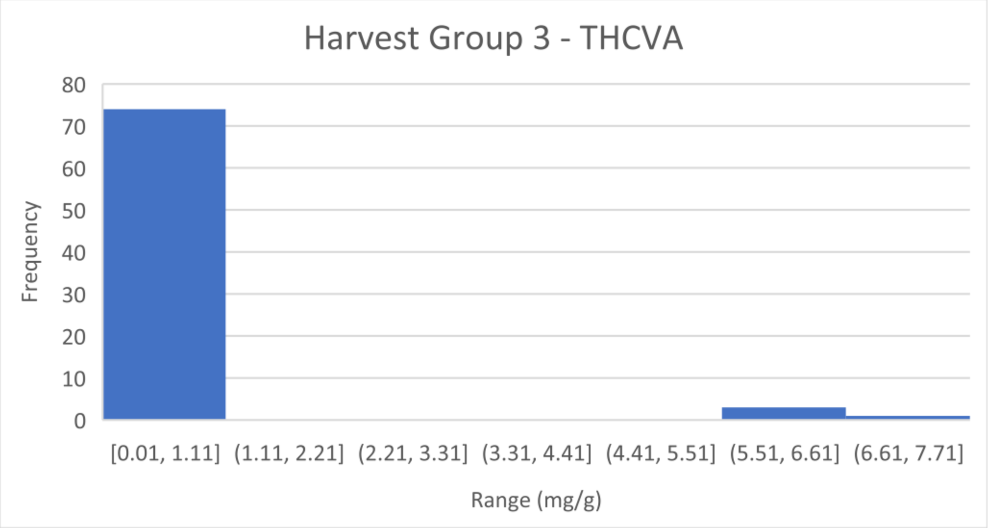
Harvest Group 3 - THCVA
80
70
60
50
40
30
20
10
0
Frequency
[0.01, 1.11]
(1.11, 2.21]
(2.21, 3.31]
(3.31, 4.41]
(4.41, 5.51]
(5.51, 6.61]
(6.61, 7.71]
Range (mg/g)

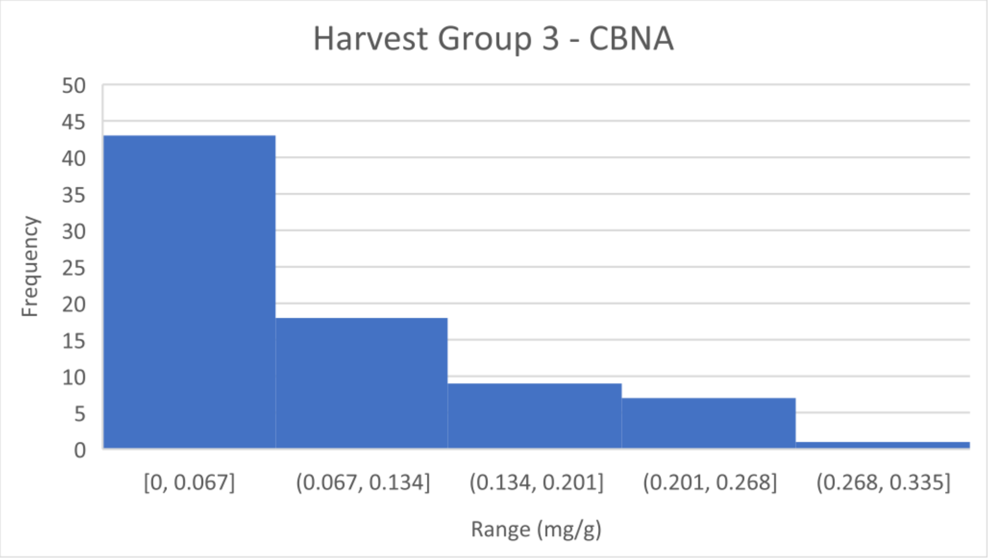
Harvest Group 3 - CBNA
Frequency
50
45
40
35
30
25
20
15
10
5
0
[0, 0.067]
(0.067, 0.134]
(0.134, 0.201]
(0.201, 0.268]
(0.268, 0.335]
Range (mg/g)

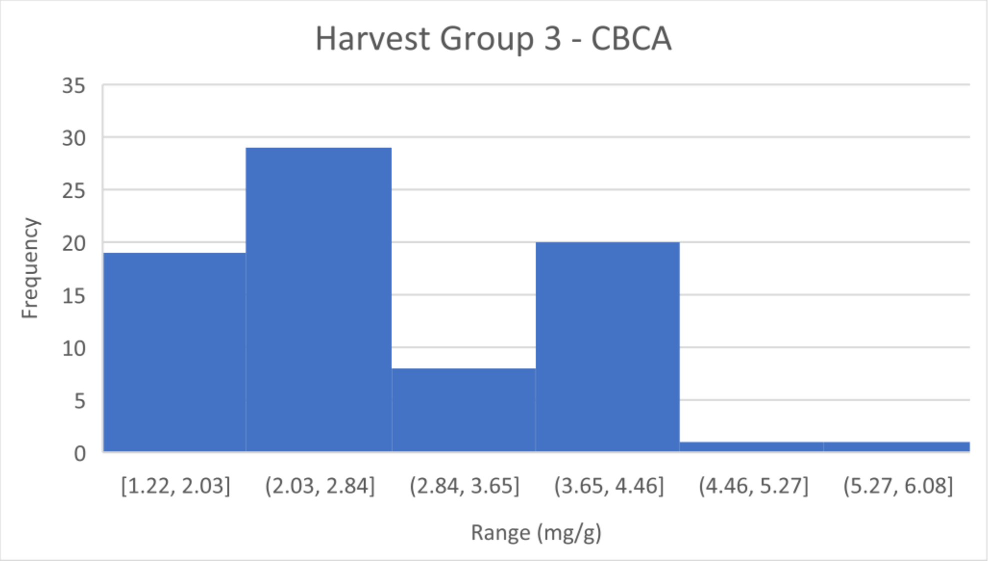
Harvest Group 3 - CBCA
Frequency
35
30
25
20
15
10
5
0
[1.22, 2.03]
(2.03, 2.84]
(2.84, 3.65]
(3.65, 4.46]
(4.46, 5.27]
(5.27, 6.08]
Range (mg/g)

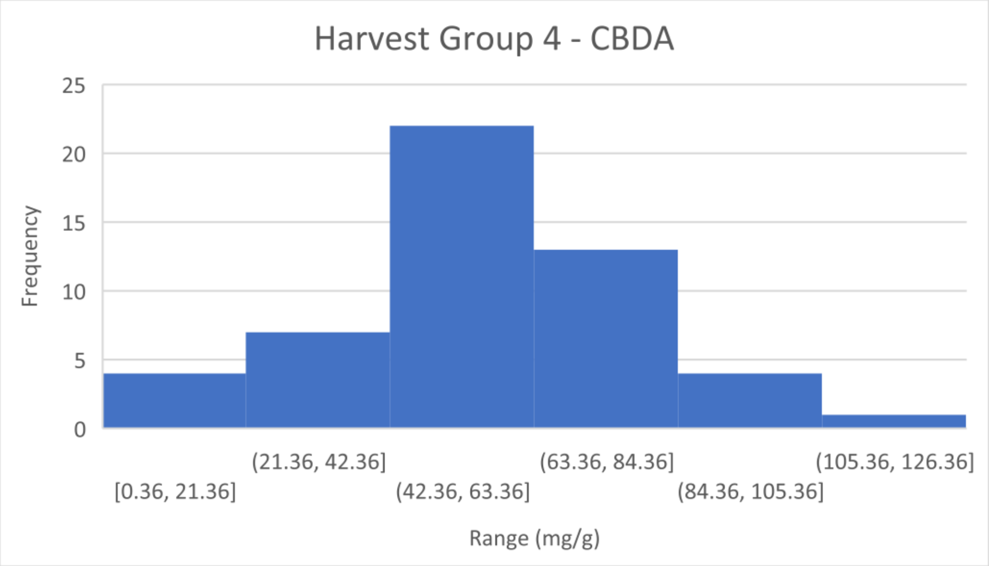
Harvest Group 4 - CBDA
25
20
15
10
5
0
Frequency
[0.36, 21.36]
(21.36, 42.36]
(42.36, 63.36]
(63.36, 84.36]
(84.36, 105.36]
(105.36, 126.36]
Range (mg/g)

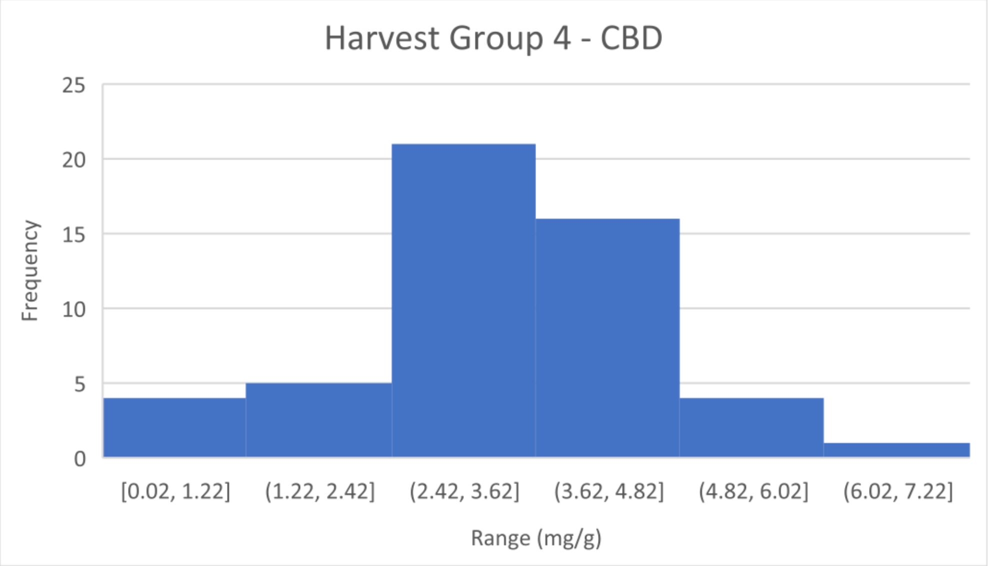
Harvest Group 4 - CBD
25
20
15
10
5
0
Frequency
[0.02, 1.22]
(1.22, 2.42]
(2.42, 3.62]
(3.62, 4.82]
(4.82, 6.02]
(6.02, 7.22]
Range (mg/g)

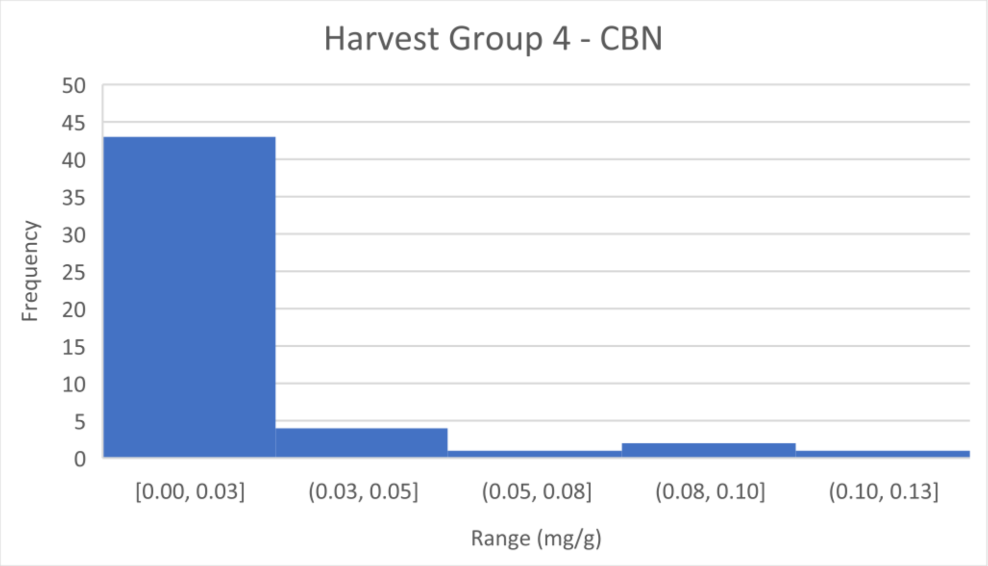
Harvest Group 4 - CBN
Frequency
50
45
40
35
30
25
20
15
10
5
0
[0.00, 0.03]
(0.03, 0.05]
(0.05, 0.08]
(0.08, 0.10]
(0.10, 0.13]
Range (mg/g)

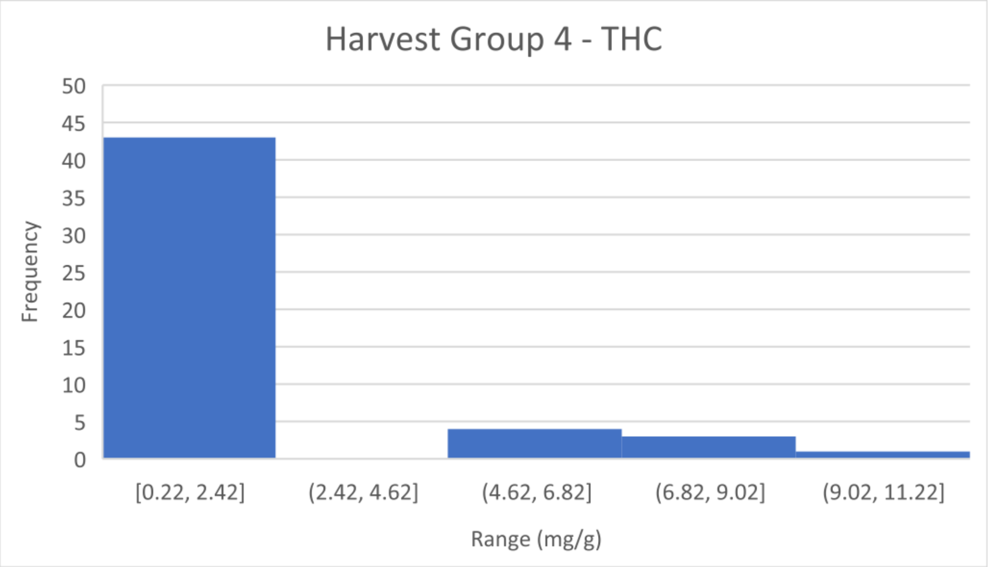
Harvest Group 4 - THC
Frequency
50
45
40
35
30
25
20
15
10
5
0
[0.22, 2.42]
(2.42, 4.62]
(4.62, 6.82]
(6.82, 9.02]
(9.02, 11.22]
Range (mg/g)

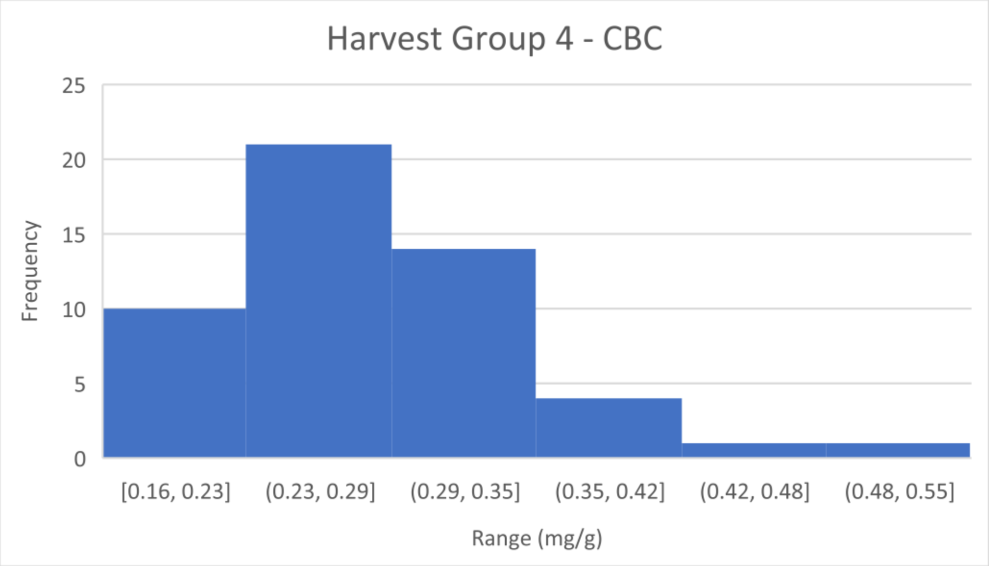
Harvest Group 4 - CBC
Frequency
25
20
15
10
5
0
[0.16, 0.23]
(0.23, 0.29]
(0.29, 0.35]
(0.35, 0.42]
(0.42, 0.48]
(0.48, 0.55]
Range (mg/g)

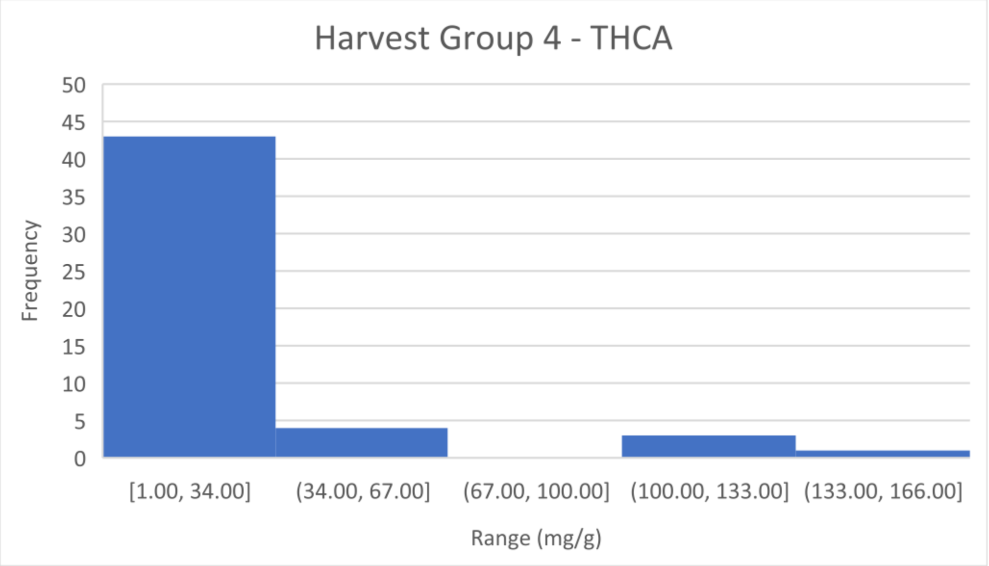
Harvest Group 4 - THCA
Frequency
50
45
40
35
30
25
20
15
10
5
0
[1.00, 34.00]
(34.00, 67.00]
(67.00, 100.00]
(100.00, 133.00]
(133.00, 166.00]
Range (mg/g)

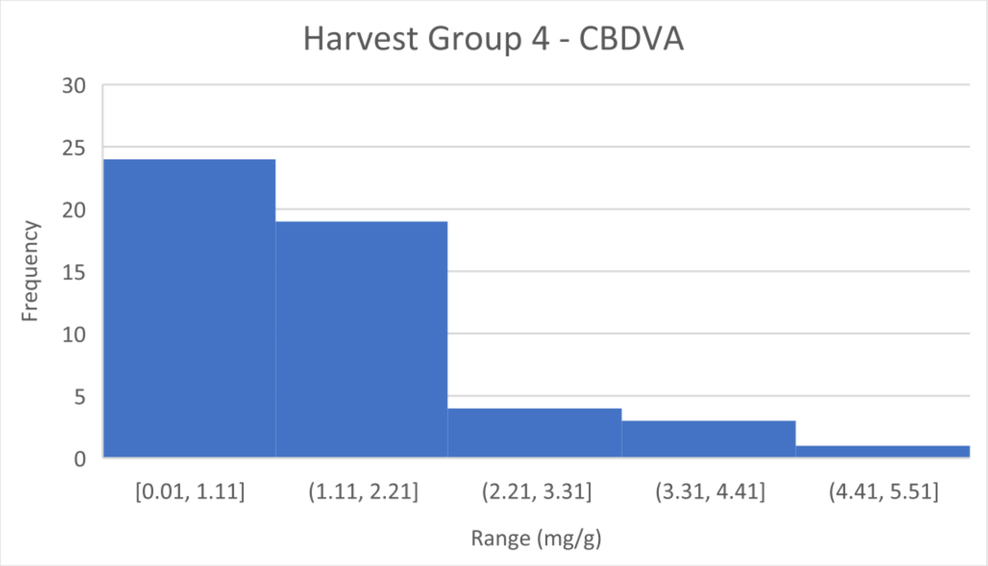
Harvest Group 4 - CBDVA
30
25
20
15
10
5
0
Frequency
[0.01, 1.11]
(1.11, 2.21]
(2.21, 3.31]
(3.31, 4.41]
(4.41, 5.51]
Range (mg/g)

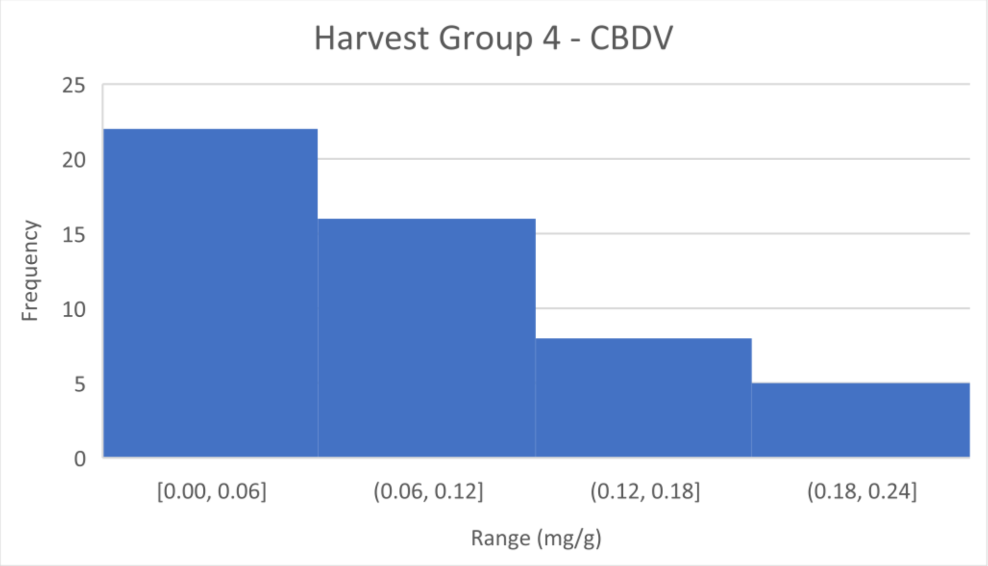
Harvest Group 4 - CBDV
25
20
15
10
5
0
Frequency
[0.00, 0.06]
(0.06, 0.12]
(0.12, 0.18]
(0.18, 0.24]
Range (mg/g)

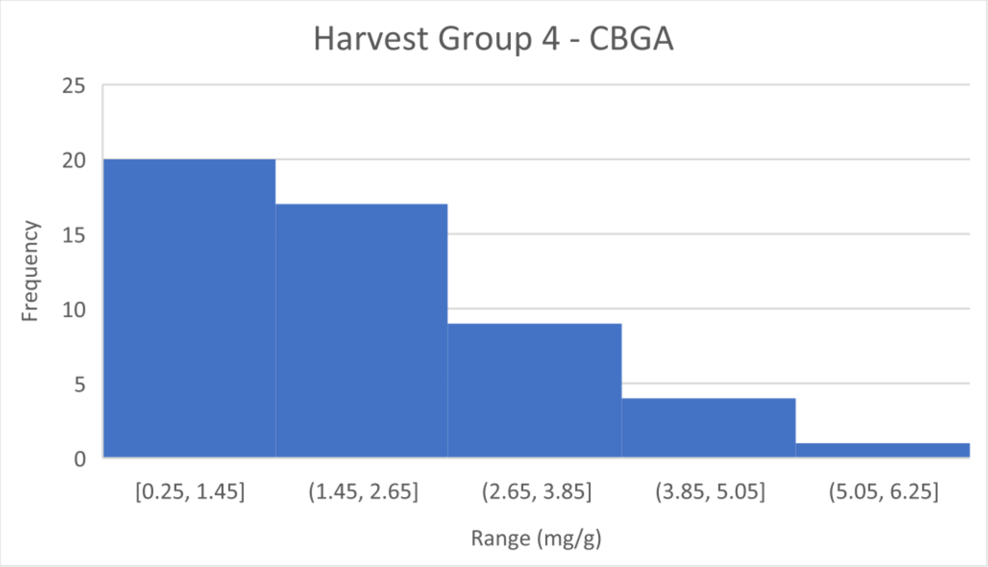
Harvest Group 4 - CBGA
25
20
15
10
5
0
Frequency
[0.25, 1.45]
(1.45, 2.65]
(2.65, 3.85]
(3.85, 5.05]
(5.05, 6.25]
Range (mg/g)

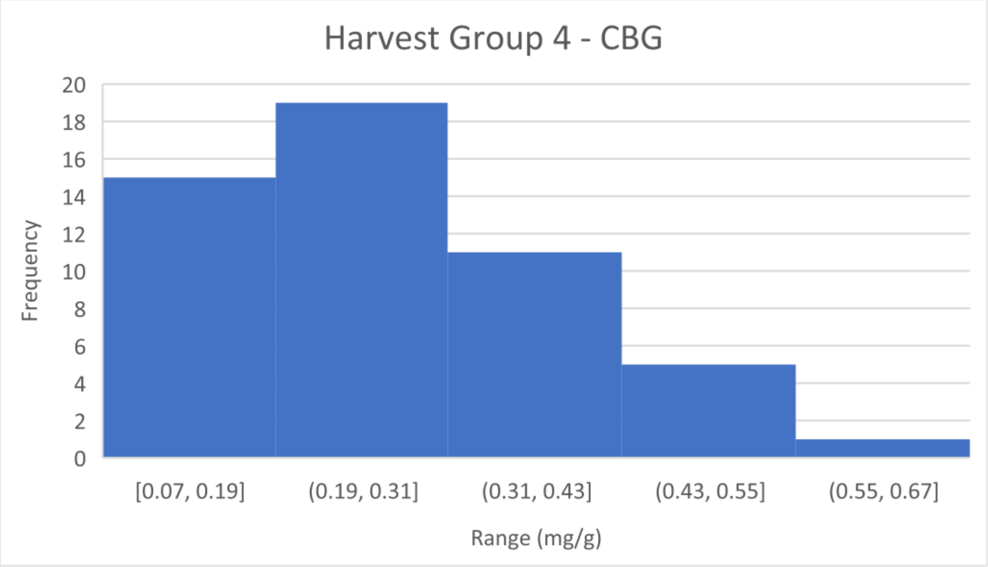
Harvest Group 4 - CBG
20
18
16
14
12
10
8
6
4
2
0
Frequency
[0.07, 0.19]
(0.19, 0.31]
(0.31, 0.43]
(0.43, 0.55]
(0.55, 0.67]
Range (mg/g)

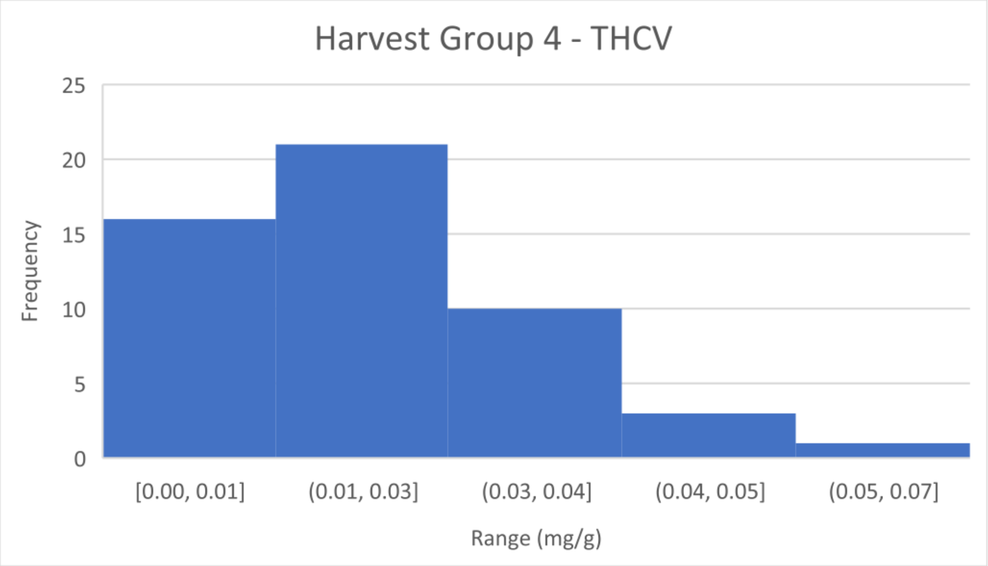
Harvest Group 4 - THCV
Frequency
25
20
15
10
5
0
[0.00, 0.01]
(0.01, 0.03]
(0.03, 0.04]
(0.04, 0.05]
(0.05, 0.07]
Range (mg/g)

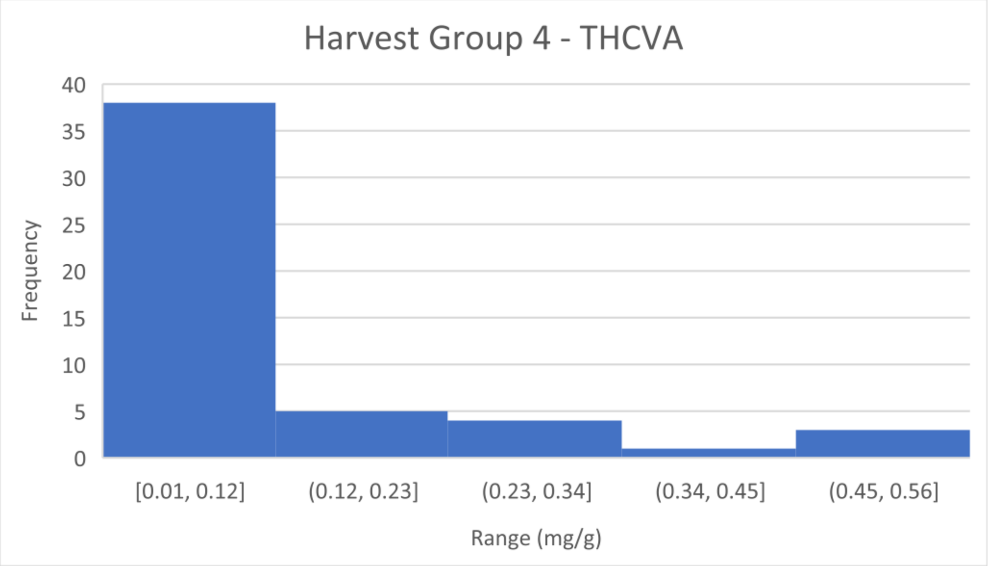
Harvest Group 4 - THCVA
Frequency
40
35
30
25
20
15
10
5
0
[0.01, 0.12]
(0.12, 0.23]
(0.23, 0.34]
(0.34, 0.45]
(0.45, 0.56]
Range (mg/g)

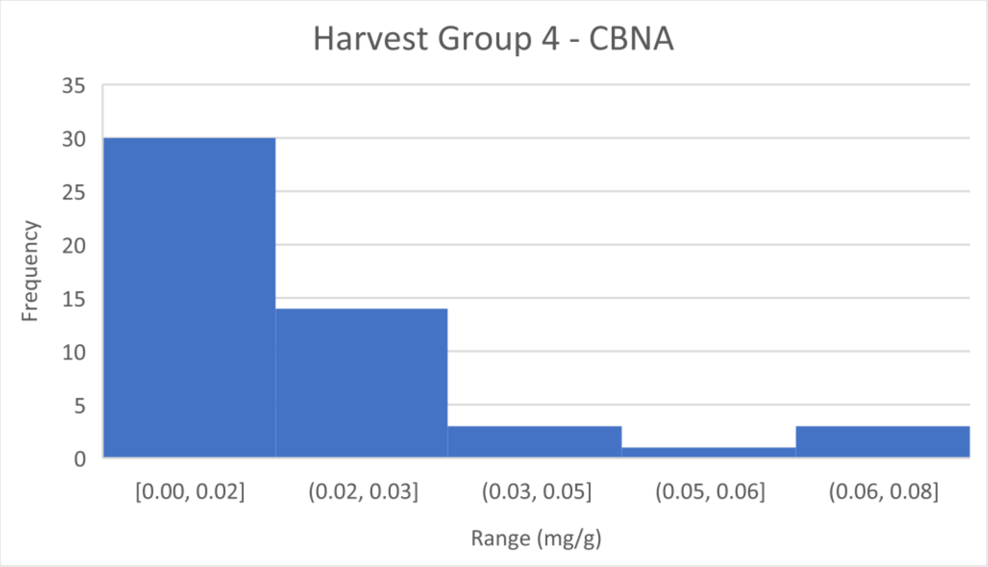
Harvest Group 4 - CBNA
Frequency
35
30
25
20
15
10
5
0
[0.00, 0.02]
(0.02, 0.03]
(0.03, 0.05]
(0.05, 0.06]
(0.06, 0.08]
Range (mg/g)

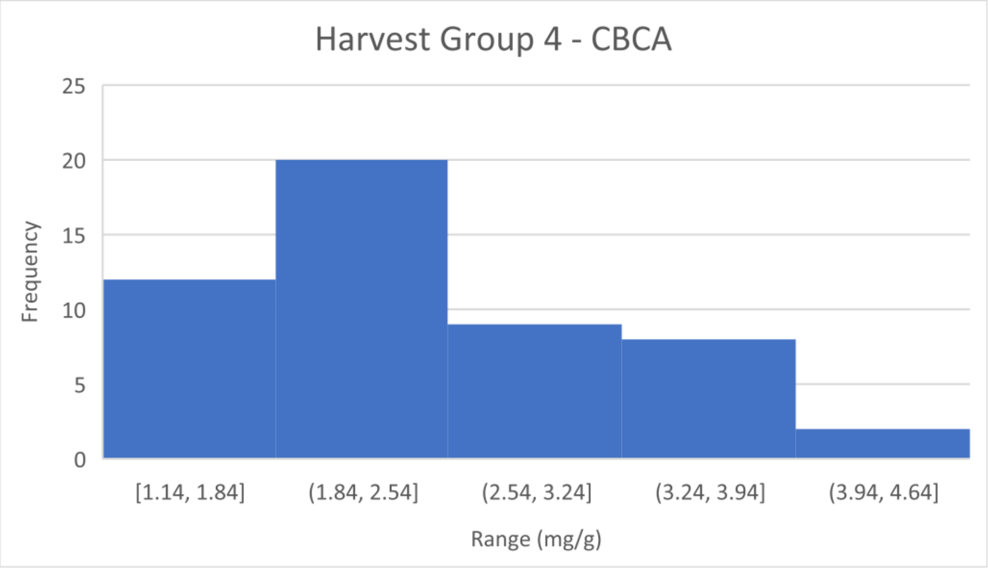
Harvest Group 4 - CBCA
Frequency
25
20
15
10
5
0
[1.14, 1.84]
(1.84, 2.54]
(2.54, 3.24]
(3.24, 3.94]
(3.94, 4.64]
Range (mg/g)
