# Supplementary material for: Developing Prediction Models Using Near-Infrared Spectroscopy to Quantify Cannabinoid Content in Cannabis Sativa
Source: Sensors (Basel). 2023 Feb 27;23(5):2607. doi: 10.3390/s23052607 (PMC10007171; doi:10.3390/s23052607)
Supplement: Supplementary file 1 [file sensors-23-02607-s001.zip › sensors-2200076-supplementary/Figure S2 Score Plot of Two Principal Components of the Entire Cannabis Sample Set Using the Bruker MPA II FT-NIR Spectrometer .pdf]

Analysis Report

Generated by vic74qy@WL2YR95M3 on 06-Jan-2023 13:56:54

Model

Principal Components Analysis Model
Developed 06-Jan-2023 13:51:26.690
Author: vic74qy@WL2YR95M3
X-block: AllHarvestsUnaveragedBrukerNIR22MM.xlsx 2202 by 949
(vic74qy@WL2YR95M3@20221104T154753.53527777 m:20221104154754.067)
Included: [1-2202] [1-949]
Included (in axis units): [n/a] [11536-3952]
Preprocessing: Detrend, SNV, 2nd Derivative (order: 2, window: 5 pt, tails: weighted), Mean Center
Num. PCs: 5
Algorithm: SVD
Cross validation: venetian blinds w/ 10 splits and blind thickness = 1
RMSEC: 0.000672104
RMSECV: 0.000725013

SSQ Table

Percent Variance Captured by PCA Model

Principal Component Number	Eigenvalue of Cov(X)	% Variance Captured This PC	% Variance Captured Total
1	6.20e-04	47.47	47.47
2	1.11e-04	8.50	55.97
3	7.16e-05	5.48	61.45
4	5.28e-05	4.04	65.48
5	2.20e-05	1.69	67.17

Figure S2: Score plot of two principal components of the entire cannabis sample set (unaveraged data, n = 2202) using the Bruker MPA II FT-NIR spectrometer (9000 cm^{-1} to 4000 cm^{-1} range).

Figures associated with the analysis:

Samples/Scores - PCA 5 PCs - AllHarvestsUnaveragedBrukerNIR22MM.xlsx

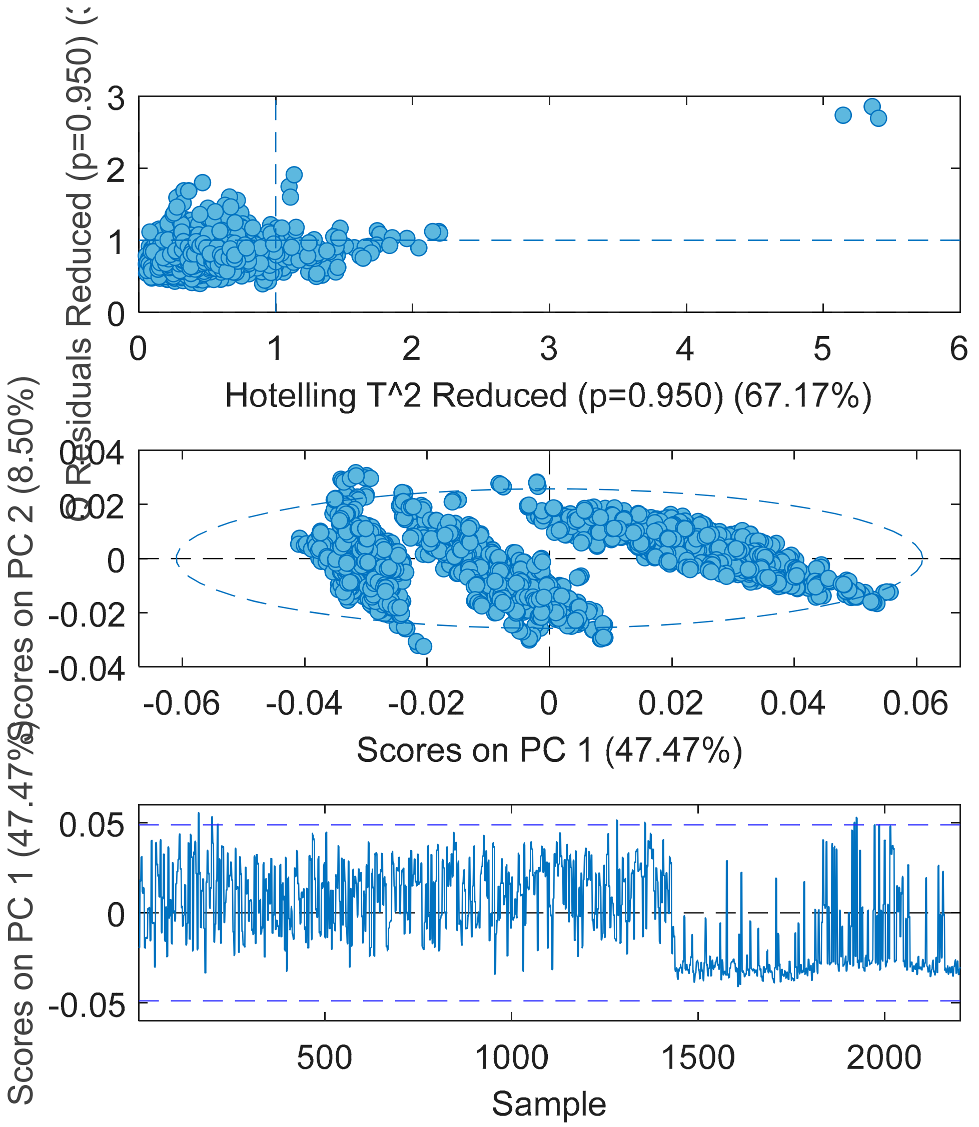

Samples/Scores - PCA 5 PCs - AllHarvestsUnaveragedBrukerNIR22MM.xlsx

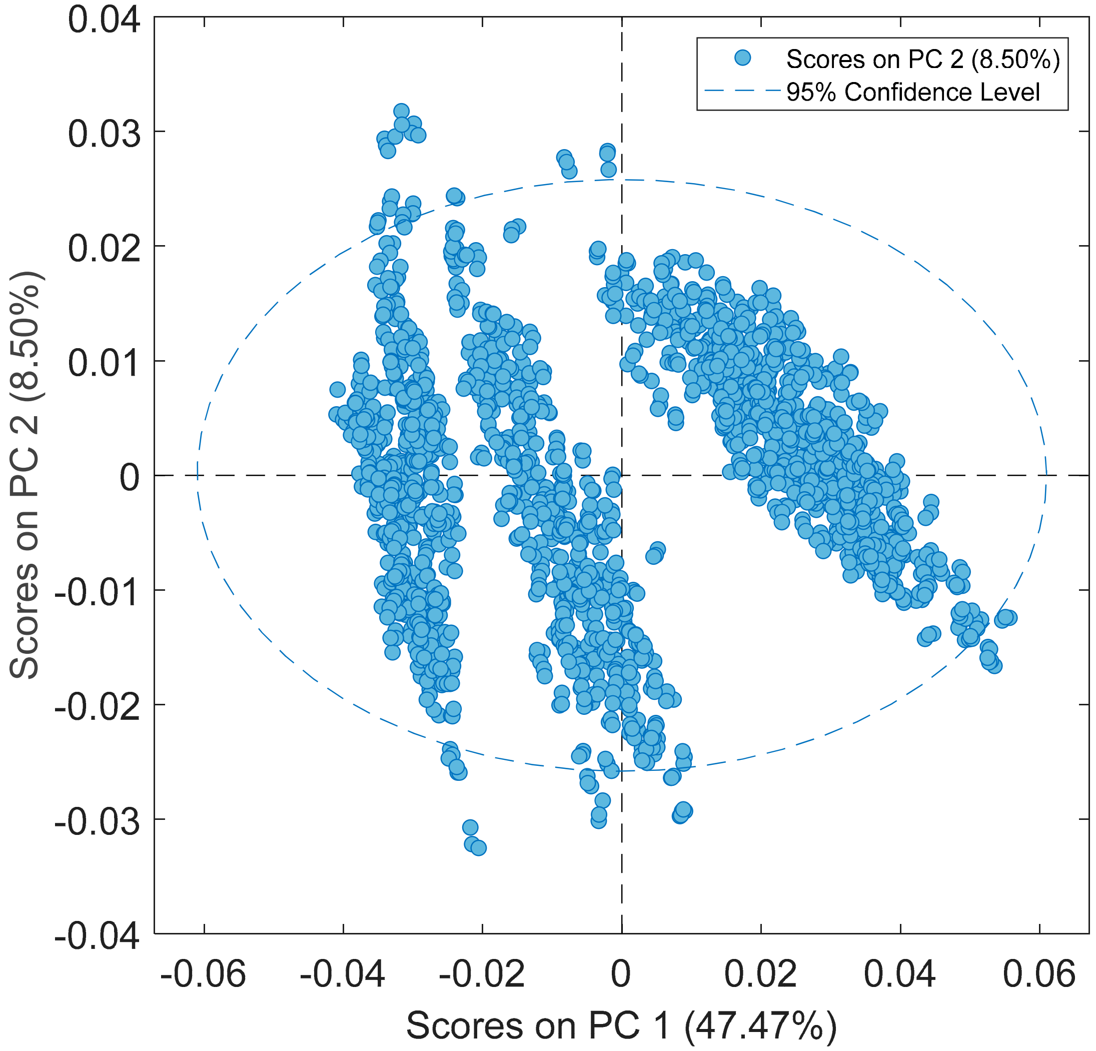

Model Statistics - PCA 5 PCs - AllHarvestsUnaveragedBrukerNIR22MM.xlsx

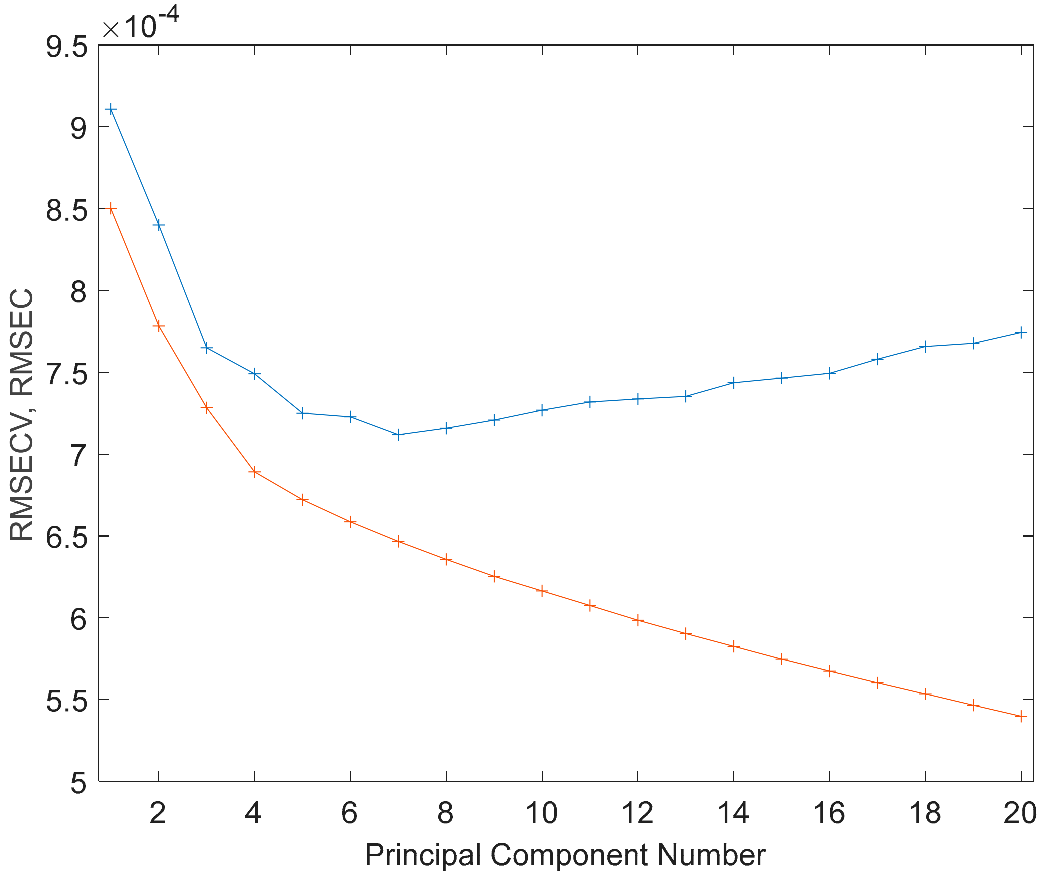

Variables/Loadings - PCA 5 PCs - AllHarvestsUnaveragedBrukerNIR22MM.xlsx

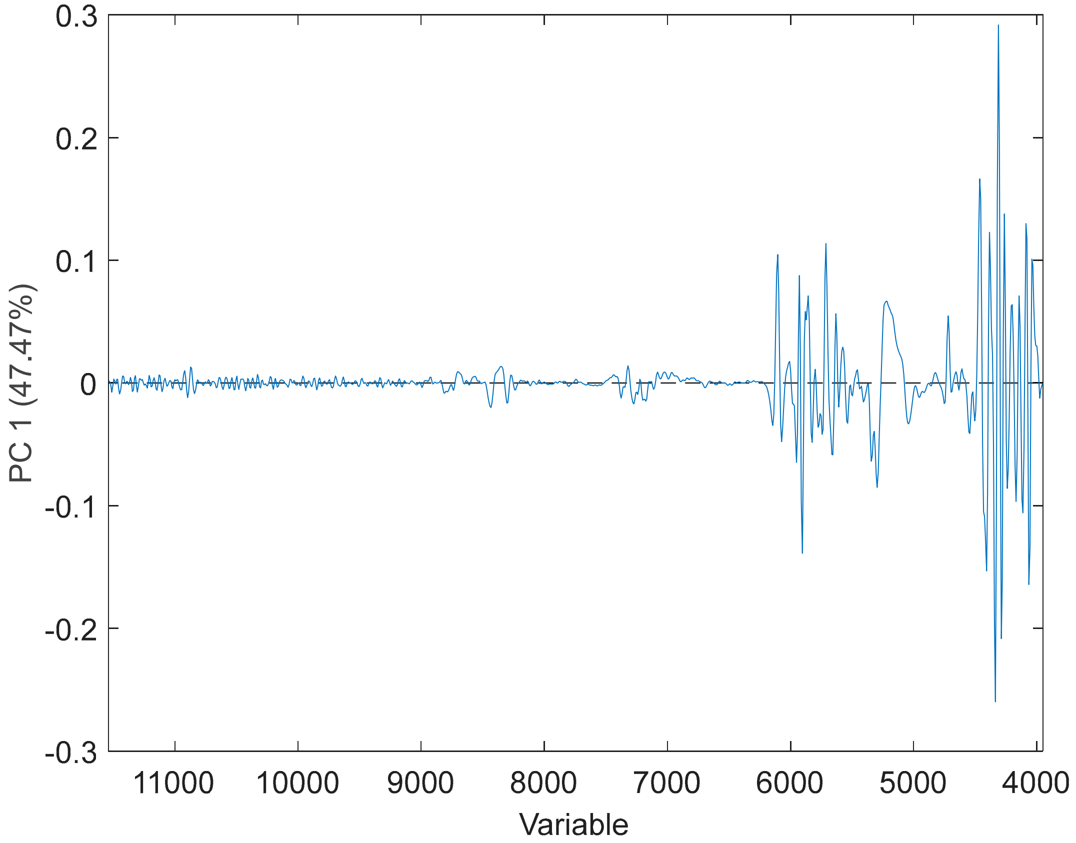

Biplot - PCA 5 PCs - AllHarvestsUnaveragedBrukerNIR22MM.xlsx

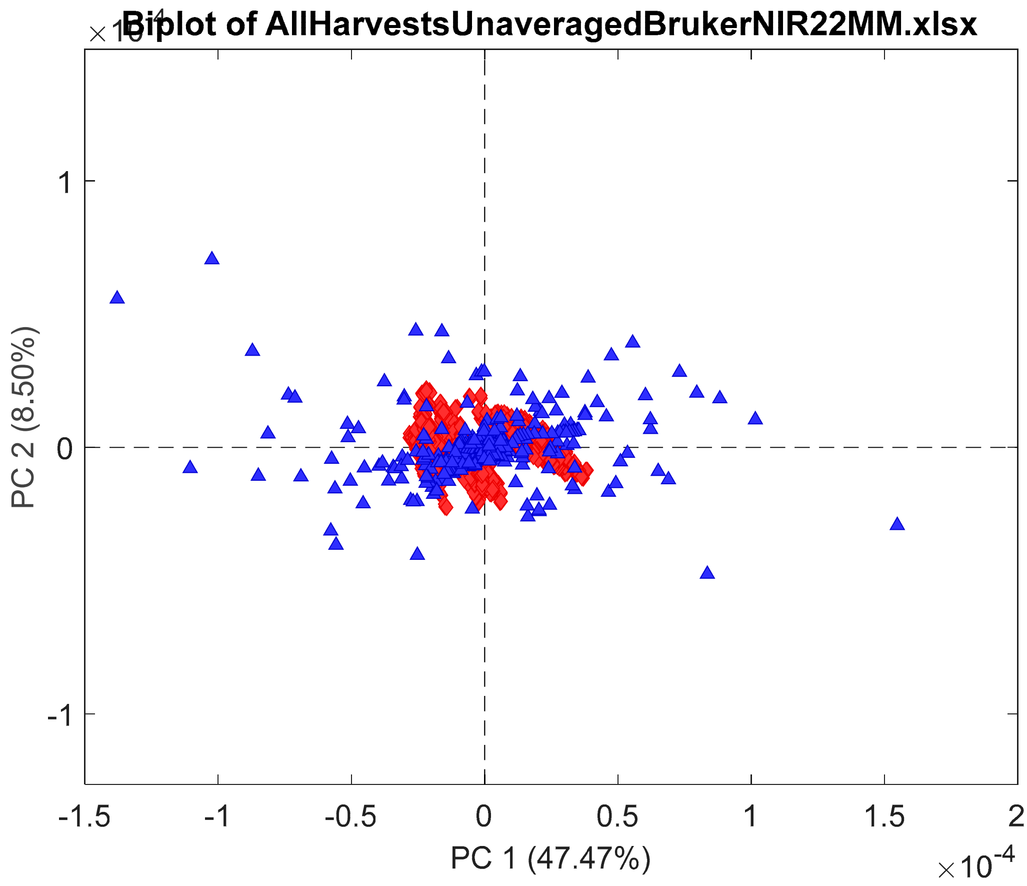
Biplot of AllHarvestsUnaveragedBrukerNIR22MM.xlsx
×10⁻⁴
1
0
-1
PC 2 (8.50%)
-1.5
-1
-0.5
0
0.5
1
1.5
2
PC 1 (47.47%)
×10⁻⁴
