# Supplementary material for: Developing Prediction Models Using Near-Infrared Spectroscopy to Quantify Cannabinoid Content in Cannabis Sativa
Source: Sensors (Basel). 2023 Feb 27;23(5):2607. doi: 10.3390/s23052607 (PMC10007171; doi:10.3390/s23052607)
Supplement: Supplementary file 1 [file sensors-23-02607-s001.zip › sensors-2200076-supplementary/Table S3 LCMS Bruker MPA II Raw Data and Classifications for PLSDA.pdf]

Table S3: Definitions of even ratio, high CBDA and high THCA in relation to the CBDA and THCA ratio using LCMS quantitation data (n = 734) for the Bruker MPA II.

List ID	**CBDA (mg/g)**	**THCA (mg/g)**	CBDA/THCA	Classification
1	35.84	14.15	2.53265	Even Ratio
2	0.29	73.53	0.00394	High THCA
3	0.39	102.92	0.00379	High THCA
4	0.24	72.84	0.00330	High THCA
5	0.33	91.69	0.00360	High THCA
6	42.58	15.54	2.74067	Even Ratio
7	56.02	20.06	2.79306	Even Ratio
8	54.79	20.07	2.73027	Even Ratio
9	0.22	45.71	0.00481	High THCA
10	77.99	39.11	1.99432	Even Ratio
11	47.99	18.49	2.59506	Even Ratio
12	0.54	116.61	0.00463	High THCA
13	59.95	23.41	2.56115	Even Ratio
14	61.23	28.44	2.15267	Even Ratio
15	26.41	1.24	21.22642	High CBDA
16	0.37	122.06	0.00303	High THCA
17	0.42	112.48	0.00373	High THCA
18	0.33	95.84	0.00344	High THCA
19	0.29	64.63	0.00449	High THCA
20	0.34	116.88	0.00291	High THCA
21	44.9	30.51	1.47166	Even Ratio
22	0.22	62.77	0.00350	High THCA
23	0.31	108.46	0.00286	High THCA
24	32.77	16.61	1.97250	Even Ratio
25	0.21	50.36	0.00417	High THCA
26	29.32	10.27	2.85508	Even Ratio
27	0.39	121.87	0.00320	High THCA
28	60.46	34.02	1.77744	Even Ratio
29	104.14	48.73	2.13709	Even Ratio
30	44.01	12.04	3.65577	Even Ratio
31	0.3	102.47	0.00293	High THCA
32	59.79	34.61	1.72735	Even Ratio
33	65.37	28.20	2.31836	Even Ratio
34	0.29	80.66	0.00360	High THCA
35	0.32	85.05	0.00376	High THCA
36	0.29	99.59	0.00291	High THCA
37	0.44	122.39	0.00360	High THCA
38	0.36	91.01	0.00396	High THCA

39	78.49	42.23	1.85873	Even Ratio
40	64.51	25.30	2.54991	Even Ratio
41	70.01	36.71	1.90717	Even Ratio
42	0.27	79.78	0.00338	High THCA
43	35.64	16.55	2.15383	Even Ratio
44	75.09	30.89	2.43082	Even Ratio
45	0.34	104.35	0.00326	High THCA
46	53	25.52	2.07701	Even Ratio
47	0.41	138.30	0.00296	High THCA
48	0.34	99.32	0.00342	High THCA
49	45.82	17.65	2.59574	Even Ratio
50	0.29	104.90	0.00276	High THCA
51	0.35	131.31	0.00267	High THCA
52	0.35	88.88	0.00394	High THCA
53	43.57	11.54	3.77416	Even Ratio
54	0.46	170.33	0.00270	High THCA
55	0.34	115.10	0.00295	High THCA
56	60.95	23.72	2.56929	Even Ratio
57	0.31	63.68	0.00487	High THCA
58	0.25	104.92	0.00238	High THCA
59	57.71	21.33	2.70580	Even Ratio
60	23.98	1.12	21.48768	High CBDA
61	0.27	93.09	0.00290	High THCA
62	0.32	104.60	0.00306	High THCA
63	0.28	57.54	0.00487	High THCA
64	0.23	63.65	0.00361	High THCA
65	0.28	95.56	0.00293	High THCA
66	0.45	147.46	0.00305	High THCA
67	0.37	129.70	0.00285	High THCA
68	57.25	15.80	3.62250	Even Ratio
69	65.8	24.99	2.63302	Even Ratio
70	0.35	82.19	0.00426	High THCA
71	0.4	143.39	0.00279	High THCA
72	47.8	25.75	1.85613	Even Ratio
73	0.3	102.91	0.00292	High THCA
74	50.71	15.34	3.30674	Even Ratio
75	0.35	109.02	0.00321	High THCA
76	0.35	120.99	0.00289	High THCA
77	74.26	28.32	2.62205	Even Ratio
78	0.21	65.50	0.00321	High THCA
79	0.3	73.25	0.00410	High THCA
80	0.35	112.87	0.00310	High THCA
81	0.24	73.56	0.00326	High THCA
82	0.29	89.78	0.00323	High THCA
83	0.3	101.69	0.00295	High THCA
84	0.3	83.00	0.00361	High THCA

85	61.42	24.33	2.52484	Even Ratio
86	27.44	12.62	2.17428	Even Ratio
87	0.4	123.99	0.00323	High THCA
88	0.34	86.26	0.00394	High THCA
89	0.27	83.37	0.00324	High THCA
90	37	16.12	2.29527	Even Ratio
91	0.34	112.97	0.00301	High THCA
92	0.29	76.69	0.00378	High THCA
93	66.55	22.46	2.96303	Even Ratio
94	46.07	16.66	2.76585	Even Ratio
95	46.52	18.23	2.55128	Even Ratio
96	0.33	98.22	0.00336	High THCA
97	0.32	91.88	0.00348	High THCA
98	0.33	79.09	0.00417	High THCA
99	68.09	29.69	2.29301	Even Ratio
100	0.25	48.09	0.00520	High THCA
101	0.27	62.97	0.00429	High THCA
102	65.39	24.44	2.67563	Even Ratio
103	38.71	22.72	1.70373	Even Ratio
104	0.33	104.28	0.00316	High THCA
105	45.89	12.07	3.80297	Even Ratio
106	0.37	112.73	0.00328	High THCA
107	0.32	91.91	0.00348	High THCA
108	0.29	53.92	0.00538	High THCA
109	0.28	67.14	0.00417	High THCA
110	50.72	20.10	2.52373	Even Ratio
111	52.13	25.85	2.01653	Even Ratio
112	0.38	103.12	0.00369	High THCA
113	29.78	13.18	2.26030	Even Ratio
114	0.61	84.76	0.00720	High THCA
115	0.31	63.85	0.00486	High THCA
116	71.69	31.17	2.30008	Even Ratio
117	0.27	70.22	0.00385	High THCA
118	80.9	46.04	1.75730	Even Ratio
119	0.39	94.79	0.00411	High THCA
120	59.27	24.71	2.39831	Even Ratio
121	85.89	36.70	2.34020	Even Ratio
122	0.28	97.62	0.00287	High THCA
123	60.11	22.52	2.66892	Even Ratio
124	65.98	21.15	3.12011	Even Ratio
125	70.02	28.43	2.46248	Even Ratio
126	64.14	21.03	3.04925	Even Ratio
127	0.37	107.38	0.00345	High THCA
128	59.34	21.06	2.81714	Even Ratio
129	0.29	86.58	0.00335	High THCA
130	0.26	71.79	0.00362	High THCA

131	39.66	12.87	3.08109	Even Ratio
132	0.46	134.40	0.00342	High THCA
133	34.2	1.27	26.90876	High CBDA
134	0.35	115.13	0.00304	High THCA
135	51.88	34.44	1.50643	Even Ratio
136	63.67	30.13	2.11330	Even Ratio
137	0.3	61.65	0.00487	High THCA
138	54.62	21.56	2.53300	Even Ratio
139	41.41	10.48	3.94972	Even Ratio
140	0.31	114.98	0.00270	High THCA
141	0.31	90.24	0.00344	High THCA
142	0.32	127.80	0.00250	High THCA
143	0.33	93.17	0.00354	High THCA
144	0.27	69.41	0.00389	High THCA
145	76	32.81	2.31627	Even Ratio
146	0.3	85.97	0.00349	High THCA
147	89.95	43.00	2.09177	Even Ratio
148	0.24	78.91	0.00304	High THCA
149	60.99	24.08	2.53305	Even Ratio
150	43.8	13.17	3.32566	Even Ratio
151	0.52	73.77	0.00705	High THCA
152	40.17	19.82	2.02642	Even Ratio
153	0.65	117.26	0.00554	High THCA
154	0.27	89.13	0.00303	High THCA
155	49	18.63	2.63008	Even Ratio
156	0.33	123.60	0.00267	High THCA
157	0.38	128.09	0.00297	High THCA
158	0.34	91.25	0.00373	High THCA
159	84.82	43.39	1.95472	Even Ratio
160	71.94	30.25	2.37840	Even Ratio
161	0.36	100.77	0.00357	High THCA
162	59.16	22.74	2.60159	Even Ratio
163	40.84	20.47	1.99544	Even Ratio
164	0.43	97.82	0.00440	High THCA
165	0.31	86.34	0.00359	High THCA
166	0.42	121.88	0.00345	High THCA
167	53.64	31.53	1.70106	Even Ratio
168	0.38	99.83	0.00381	High THCA
169	57.55	19.15	3.00469	Even Ratio
170	0.35	81.32	0.00430	High THCA
171	65.97	23.90	2.76001	Even Ratio
172	0.36	72.24	0.00498	High THCA
173	35.19	14.96	2.35171	Even Ratio
174	0.32	54.60	0.00586	High THCA
175	71.18	31.67	2.24741	Even Ratio
176	56.71	20.59	2.75361	Even Ratio

177	0.31	67.90	0.00457	High THCA
178	0.35	74.23	0.00472	High THCA
179	0.34	99.53	0.00342	High THCA
180	71.47	46.99	1.52084	Even Ratio
181	43.44	20.50	2.11861	Even Ratio
182	0.53	98.10	0.00540	High THCA
183	69.33	24.04	2.88352	Even Ratio
184	0.35	107.55	0.00325	High THCA
185	0.3	75.59	0.00397	High THCA
186	62.44	22.52	2.77247	Even Ratio
187	44.41	14.37	3.09036	Even Ratio
188	0.54	93.88	0.00575	High THCA
189	17.48	6.08	2.87431	Even Ratio
190	0.29	83.82	0.00346	High THCA
191	41.71	15.02	2.77703	Even Ratio
192	0.32	97.04	0.00330	High THCA
193	80.48	44.23	1.81977	Even Ratio
194	0.34	110.83	0.00307	High THCA
195	0.28	64.25	0.00436	High THCA
196	0.28	57.66	0.00486	High THCA
197	0.32	89.08	0.00359	High THCA
198	0.26	84.07	0.00309	High THCA
199	0.28	95.81	0.00292	High THCA
200	70.83	32.37	2.18843	Even Ratio
201	47.45	16.11	2.94561	Even Ratio
202	0.32	83.56	0.00383	High THCA
203	67.2	26.03	2.58131	Even Ratio
204	0.42	79.72	0.00527	High THCA
205	0.27	87.46	0.00309	High THCA
206	0.39	125.32	0.00311	High THCA
207	28.18	12.70	2.21869	Even Ratio
208	9.83	0.66	14.83663	High CBDA
209	0.28	100.57	0.00278	High THCA
210	0.27	76.07	0.00355	High THCA
211	22.53	9.97	2.25996	Even Ratio
212	0.34	118.30	0.00287	High THCA
213	0.33	110.49	0.00299	High THCA
214	0.33	69.98	0.00472	High THCA
215	0.36	88.46	0.00407	High THCA
216	78.59	35.65	2.20455	Even Ratio
217	0.32	97.63	0.00328	High THCA
218	0.31	101.53	0.00305	High THCA
219	70.33	47.10	1.49310	Even Ratio
220	0.34	93.50	0.00364	High THCA
221	0.34	79.96	0.00425	High THCA
222	37.5	17.34	2.16308	Even Ratio

223	38.37	13.27	2.89067	Even Ratio
224	51.67	24.59	2.10149	Even Ratio
225	72.4	28.88	2.50719	Even Ratio
226	60.98	31.24	1.95195	Even Ratio
227	0.37	110.68	0.00334	High THCA
228	0.4	102.64	0.00390	High THCA
229	0.24	67.45	0.00356	High THCA
230	60.52	25.57	2.36722	Even Ratio
231	0.29	83.54	0.00347	High THCA
232	0.43	97.66	0.00440	High THCA
233	0.3	96.96	0.00309	High THCA
234	60.06	31.10	1.93094	Even Ratio
235	0.29	73.89	0.00392	High THCA
236	0.31	86.79	0.00357	High THCA
237	0.31	102.85	0.00301	High THCA
238	54.22	20.74	2.61394	Even Ratio
239	0.26	80.77	0.00322	High THCA
240	69.96	34.72	2.01523	Even Ratio
241	63.63	26.91	2.36446	Even Ratio
242	0.29	97.69	0.00297	High THCA
243	54.94	22.95	2.39375	Even Ratio
244	0.31	109.12	0.00284	High THCA
245	0.23	70.69	0.00325	High THCA
246	0.23	77.18	0.00298	High THCA
247	0.25	104.82	0.00238	High THCA
248	0.21	73.63	0.00285	High THCA
249	0.23	79.30	0.00290	High THCA
250	0.35	107.77	0.00325	High THCA
251	0.27	59.86	0.00451	High THCA
252	0.24	66.15	0.00363	High THCA
253	0.31	91.29	0.00340	High THCA
254	29.97	13.86	2.16260	Even Ratio
255	29.42	14.74	1.99642	Even Ratio
256	74.36	25.60	2.90417	Even Ratio
257	0.32	75.36	0.00425	High THCA
258	66.39	22.77	2.91574	Even Ratio
259	0.38	122.45	0.00310	High THCA
260	68.68	29.08	2.36188	Even Ratio
261	51.23	16.10	3.18282	Even Ratio
262	0.53	99.69	0.00532	High THCA
263	42.64	15.96	2.67125	Even Ratio
264	0.34	77.21	0.00440	High THCA
265	47.27	11.43	4.13604	Even Ratio
266	0.27	85.76	0.00315	High THCA
267	0.37	95.29	0.00388	High THCA
268	0.33	110.03	0.00300	High THCA

269	40.74	14.85	2.74301	Even Ratio
270	66.62	28.68	2.32292	Even Ratio
271	72.1	23.24	3.10221	Even Ratio
272	0.4	92.83	0.00431	High THCA
273	0.3	89.63	0.00335	High THCA
274	62.91	32.49	1.93637	Even Ratio
275	66.29	25.98	2.55109	Even Ratio
276	0.34	97.11	0.00350	High THCA
277	0.29	85.75	0.00338	High THCA
278	0.46	87.37	0.00526	High THCA
279	0.22	72.30	0.00304	High THCA
280	0.29	102.99	0.00282	High THCA
281	0.25	85.53	0.00292	High THCA
282	0.34	131.32	0.00259	High THCA
283	0.45	131.66	0.00342	High THCA
284	0.39	108.73	0.00359	High THCA
285	53.95	26.66	2.02394	Even Ratio
286	0.29	104.22	0.00278	High THCA
287	72.64	21.60	3.36352	Even Ratio
288	50.1	23.66	2.11708	Even Ratio
289	48.58	17.77	2.73396	Even Ratio
290	0.27	78.51	0.00344	High THCA
291	0.35	94.29	0.00371	High THCA
292	0.26	79.57	0.00327	High THCA
293	48.99	26.95	1.81749	Even Ratio
294	57.63	22.73	2.53558	Even Ratio
295	0.3	52.00	0.00577	High THCA
296	0.33	92.86	0.00355	High THCA
297	0.25	56.85	0.00440	High THCA
298	48.28	19.17	2.51890	Even Ratio
299	0.29	77.13	0.00376	High THCA
300	0.4	114.13	0.00350	High THCA
301	0.28	62.52	0.00448	High THCA
302	64.44	20.73	3.10910	Even Ratio
303	62.79	27.71	2.26576	Even Ratio
304	76.12	34.14	2.22948	Even Ratio
305	63.75	28.62	2.22719	Even Ratio
306	0.35	87.38	0.00401	High THCA
307	0.3	97.60	0.00307	High THCA
308	0.34	100.94	0.00337	High THCA
309	0.33	116.64	0.00283	High THCA
310	0.29	81.36	0.00356	High THCA
311	0.3	88.95	0.00337	High THCA
312	0.23	59.07	0.00389	High THCA
313	0.36	101.51	0.00355	High THCA
314	53.84	18.86	2.85411	Even Ratio

315	66.76	30.22	2.20890	Even Ratio
316	58.91	28.15	2.09264	Even Ratio
317	0.31	122.31	0.00253	High THCA
318	0.35	125.36	0.00279	High THCA
319	17.14	0.82	20.94682	High CBDA
320	43.87	17.72	2.47613	Even Ratio
321	65.03	34.05	1.90998	Even Ratio
322	0.31	64.20	0.00483	High THCA
323	66.71	33.53	1.98956	Even Ratio
324	0.3	82.11	0.00365	High THCA
325	0.38	118.54	0.00321	High THCA
326	0.25	80.47	0.00311	High THCA
327	79.39	32.69	2.42868	Even Ratio
328	0.36	73.01	0.00493	High THCA
329	0.34	91.94	0.00370	High THCA
330	27.12	9.92	2.73268	Even Ratio
331	0.31	71.17	0.00436	High THCA
332	0.24	69.00	0.00348	High THCA
333	74.14	21.70	3.41643	Even Ratio
334	0.32	85.89	0.00373	High THCA
335	66.67	27.68	2.40836	Even Ratio
336	60.34	25.94	2.32622	Even Ratio
337	0.28	72.26	0.00387	High THCA
338	61.77	31.12	1.98505	Even Ratio
339	0.26	69.28	0.00375	High THCA
340	0.36	92.41	0.00390	High THCA
341	0.25	78.11	0.00320	High THCA
342	0.28	71.67	0.00391	High THCA
343	0.22	53.62	0.00410	High THCA
344	13.26	6.09	2.17695	Even Ratio
345	0.39	111.64	0.00349	High THCA
346	65.87	24.00	2.74474	Even Ratio
347	0.36	88.33	0.00408	High THCA
348	0.24	65.34	0.00367	High THCA
349	0.22	63.24	0.00348	High THCA
350	0.25	80.90	0.00309	High THCA
351	0.38	118.31	0.00321	High THCA
352	0.33	114.33	0.00289	High THCA
353	0.25	74.87	0.00334	High THCA
354	0.23	68.71	0.00335	High THCA
355	0.27	75.32	0.00358	High THCA
356	0.27	83.59	0.00323	High THCA
357	29.81	12.06	2.47119	Even Ratio
358	0.28	105.39	0.00266	High THCA
359	0.23	69.91	0.00329	High THCA
360	0.29	102.84	0.00282	High THCA

361	0.34	109.27	0.00311	High THCA
362	0.28	102.96	0.00272	High THCA
363	0.32	103.87	0.00308	High THCA
364	0.29	81.96	0.00354	High THCA
365	0.28	91.49	0.00306	High THCA
366	49.4	14.12	3.49810	Even Ratio
367	0.32	94.36	0.00339	High THCA
368	0.3	61.16	0.00491	High THCA
369	62.18	38.65	1.60895	Even Ratio
370	14.76	6.25	2.36205	Even Ratio
371	1.23	56.33	0.02184	High THCA
372	0.31	99.94	0.00310	High THCA
373	0.29	105.75	0.00274	High THCA
374	0.35	125.68	0.00278	High THCA
375	0.27	83.39	0.00324	High THCA
376	0.27	85.08	0.00317	High THCA
377	0.35	148.84	0.00235	High THCA
378	0.36	112.20	0.00321	High THCA
379	0.28	106.31	0.00263	High THCA
380	60.5	32.84	1.84240	Even Ratio
381	0.31	99.37	0.00312	High THCA
382	83.37	40.04	2.08233	Even Ratio
383	0.38	136.29	0.00279	High THCA
384	0.36	114.91	0.00313	High THCA
385	62.43	30.96	2.01617	Even Ratio
386	0.33	95.89	0.00344	High THCA
387	0.3	100.10	0.00300	High THCA
388	44.71	31.39	1.42416	Even Ratio
389	0.33	99.25	0.00332	High THCA
390	38.49	14.19	2.71190	Even Ratio
391	0.33	130.84	0.00252	High THCA
392	75.01	24.58	3.05183	Even Ratio
393	19.83	9.97	1.98849	Even Ratio
394	0.33	81.97	0.00403	High THCA
395	0.23	43.98	0.00523	High THCA
396	0.25	81.23	0.00308	High THCA
397	29.07	12.04	2.41486	Even Ratio
398	0.23	73.92	0.00311	High THCA
399	0.33	88.30	0.00374	High THCA
400	0.29	89.31	0.00325	High THCA
401	0.21	68.89	0.00305	High THCA
402	0.28	95.33	0.00294	High THCA
403	0.33	103.13	0.00320	High THCA
404	0.27	84.58	0.00319	High THCA
405	0.32	118.53	0.00270	High THCA
406	66.59	23.57	2.82517	Even Ratio

407	67.4	18.40	3.66338	Even Ratio
408	68.11	26.48	2.57243	Even Ratio
409	0.42	92.22	0.00455	High THCA
410	73.01	22.18	3.29115	Even Ratio
411	76.43	35.16	2.17355	Even Ratio
412	0.4	85.32	0.00469	High THCA
413	0.31	75.91	0.00408	High THCA
414	40.88	20.43	2.00145	Even Ratio
415	54.85	15.37	3.56802	Even Ratio
416	0.5	87.77	0.00570	High THCA
417	47.31	16.49	2.86951	Even Ratio
418	0.28	69.90	0.00401	High THCA
419	0.3	108.82	0.00276	High THCA
420	0.45	117.71	0.00382	High THCA
421	0.27	88.67	0.00304	High THCA
422	59.97	20.53	2.92160	Even Ratio
423	92.26	46.57	1.98117	Even Ratio
424	0.23	60.99	0.00377	High THCA
425	0.33	40.90	0.00807	High THCA
426	44.42	16.83	2.63899	Even Ratio
427	42.91	13.37	3.20867	Even Ratio
428	0.59	150.49	0.00392	High THCA
429	55.98	41.38	1.35280	Even Ratio
430	0.3	91.64	0.00327	High THCA
431	41.38	16.88	2.45179	Even Ratio
432	0.24	46.78	0.00513	High THCA
433	0.33	117.53	0.00281	High THCA
434	0.3	92.26	0.00325	High THCA
435	0.24	62.65	0.00383	High THCA
436	61.56	23.75	2.59244	Even Ratio
437	36.45	16.44	2.21755	Even Ratio
438	0.31	102.36	0.00303	High THCA
439	59.41	19.33	3.07278	Even Ratio
440	0.27	48.31	0.00559	High THCA
441	0.33	121.46	0.00272	High THCA
442	0.27	90.62	0.00298	High THCA
443	50.69	15.43	3.28552	Even Ratio
444	0.3	87.87	0.00341	High THCA
445	0.28	101.22	0.00277	High THCA
446	0.31	111.40	0.00278	High THCA
447	0.28	81.68	0.00343	High THCA
448	0.31	92.13	0.00336	High THCA
449	0.28	84.30	0.00332	High THCA
450	0.24	82.38	0.00291	High THCA
451	58	19.21	3.01968	Even Ratio
452	0.28	88.28	0.00317	High THCA

453	0.45	136.74	0.00329	High THCA
454	62.48	23.14	2.69989	Even Ratio
455	0.45	100.94	0.00446	High THCA
456	0.29	122.63	0.00236	High THCA
457	0.33	134.97	0.00245	High THCA
458	0.4	133.06	0.00301	High THCA
459	0.29	100.99	0.00287	High THCA
460	0.4	141.30	0.00283	High THCA
461	0.26	63.01	0.00413	High THCA
462	0.26	40.25	0.00646	High THCA
463	0.32	82.85	0.00386	High THCA
464	0.28	81.98	0.00342	High THCA
465	74.41	37.20	2.00022	Even Ratio
466	33.62	13.43	2.50377	Even Ratio
467	0.27	80.22	0.00337	High THCA
468	0.22	51.86	0.00424	High THCA
469	0.47	129.67	0.00362	High THCA
470	0.26	77.28	0.00336	High THCA
471	67.64	26.67	2.53588	Even Ratio
472	0.31	93.16	0.00333	High THCA
473	0.26	61.71	0.00421	High THCA
474	66.52	31.39	2.11937	Even Ratio
475	0.33	101.96	0.00324	High THCA
476	0.29	84.47	0.00343	High THCA
477	29.95	8.41	3.56084	Even Ratio
478	0.21	32.18	0.00653	High THCA
479	62.4	29.44	2.11966	Even Ratio
480	87.88	2.22	39.58559	High CBDA
481	69	1.81	38.12155	High CBDA
482	56.06	1.69	33.17160	High CBDA
483	69.53	1.75	39.73143	High CBDA
484	70.08	1.86	37.67742	High CBDA
485	86.38	2.39	36.14226	High CBDA
486	58.27	1.31	44.48092	High CBDA
487	74.57	1.51	49.38411	High CBDA
488	94.61	44.64	2.11940	Even Ratio
489	87.46	2.12	41.25472	High CBDA
490	97.95	3.09	31.69903	High CBDA
491	64.72	1.9	34.06316	High CBDA
492	71.12	1.22	58.29508	High CBDA
493	99.69	2.42	41.19421	High CBDA
494	113.97	2.52	45.22619	High CBDA
495	77.35	41.46	1.86565	Even Ratio
496	69.44	1.48	46.91892	High CBDA
497	51.71	1.43	36.16084	High CBDA
498	81.15	1.98	40.98485	High CBDA

499	96.87	1.95	49.67692	High CBDA
500	101.91	2.58	39.50000	High CBDA
501	67.54	1.57	43.01911	High CBDA
502	62.34	1.51	41.28477	High CBDA
503	86.93	1.76	49.39205	High CBDA
504	74.83	2.21	33.85973	High CBDA
505	60.73	1.12	54.22321	High CBDA
506	97.32	2.28	42.68421	High CBDA
507	122.48	51.64	2.37180	Even Ratio
508	76.39	1.33	57.43609	High CBDA
509	75.31	1.96	38.42347	High CBDA
510	68.89	1.99	34.61809	High CBDA
511	109.6	2.75	39.85455	High CBDA
512	52.24	1.3	40.18462	High CBDA
513	71.43	1.24	57.60484	High CBDA
514	77.84	1.87	41.62567	High CBDA
515	75.46	1.72	43.87209	High CBDA
516	89.16	2.22	40.16216	High CBDA
517	93.84	2.43	38.61728	High CBDA
518	80.51	1.99	40.45729	High CBDA
519	64.97	1.39	46.74101	High CBDA
520	85.39	47.9	1.78267	Even Ratio
521	58.88	1.54	38.23377	High CBDA
522	84.54	1.54	54.89610	High CBDA
523	92.81	2.64	35.15530	High CBDA
524	101.25	2.47	40.99190	High CBDA
525	61.39	1.29	47.58915	High CBDA
526	0.72	114.56	0.00628	High THCA
527	69.76	1.7	41.03529	High CBDA
528	56.26	1.26	44.65079	High CBDA
529	73.02	1.78	41.02247	High CBDA
530	87.62	1.9	46.11579	High CBDA
531	88.42	1.9	46.53684	High CBDA
532	83.36	1.65	50.52121	High CBDA
533	85.9	2.62	32.78626	High CBDA
534	88.76	2.48	35.79032	High CBDA
535	92.04	2.77	33.22744	High CBDA
536	63.81	1.59	40.13208	High CBDA
537	73.19	37.4	1.95695	Even Ratio
538	58.03	1.65	35.16970	High CBDA
539	0.33	113.27	0.00291	High THCA
540	89.98	2.44	36.87705	High CBDA
541	84.7	2.04	41.51961	High CBDA
542	71.04	1.43	49.67832	High CBDA
543	63.85	1.6	39.90625	High CBDA
544	120.4	3.6	33.44444	High CBDA

545	68.78	1.36	50.57353	High CBDA
546	50.98	0.93	54.81720	High CBDA
547	102.54	42.39	2.41897	Even Ratio
548	79.48	1.82	43.67033	High CBDA
549	80.44	2.25	35.75111	High CBDA
550	87.29	3.31	26.37160	High CBDA
551	59.31	1.23	48.21951	High CBDA
552	96.47	2.77	34.82671	High CBDA
553	75.11	1.83	41.04372	High CBDA
554	102.46	42.43	2.41480	Even Ratio
555	54	1.15	46.95652	High CBDA
556	69.54	1.66	41.89157	High CBDA
557	73.07	1.53	47.75817	High CBDA
558	48.47	1.01	47.99010	High CBDA
559	72.5	1.83	39.61749	High CBDA
560	64.79	1.33	48.71429	High CBDA
561	88.06	2.4	36.69167	High CBDA
562	121.15	2.88	42.06597	High CBDA
563	62.49	1.73	36.12139	High CBDA
564	77.73	1.55	50.14839	High CBDA
565	66.13	1.53	43.22222	High CBDA
566	82.91	2.02	41.04455	High CBDA
567	65.34	1.45	45.06207	High CBDA
568	56.24	1.25	44.99200	High CBDA
569	78.54	1.96	40.07143	High CBDA
570	0.6	117.11	0.00512	High THCA
571	83.17	2.15	38.68372	High CBDA
572	94.58	43.87	2.15592	Even Ratio
573	97.92	2.37	41.31646	High CBDA
574	56.23	1.78	31.58989	High CBDA
575	87.12	1.9	45.85263	High CBDA
576	68.01	1.83	37.16393	High CBDA
577	77.24	2.25	34.32889	High CBDA
578	84.21	1.79	47.04469	High CBDA
579	53.01	1.22	43.45082	High CBDA
580	50.12	1.13	44.35398	High CBDA
581	67.04	1.9	35.28421	High CBDA
582	76.31	1.66	45.96988	High CBDA
583	87.51	2.23	39.24215	High CBDA
584	53.11	1.19	44.63025	High CBDA
585	59.32	1.61	36.84472	High CBDA
586	67.88	1.75	38.78857	High CBDA
587	90.77	35.04	2.59047	Even Ratio
588	70.52	2.21	31.90950	High CBDA
589	67.26	1.6	42.03750	High CBDA
590	116.9	55.71	2.09837	Even Ratio

591	86.08	2.04	42.19608	High CBDA
592	79.7	1.74	45.80460	High CBDA
593	79.37	2.45	32.39592	High CBDA
594	79.48	2.07	38.39614	High CBDA
595	0.26	89.01	0.00292	High THCA
596	79.11	1.78	44.44382	High CBDA
597	73.02	2.08	35.10577	High CBDA
598	66.2	1.64	40.36585	High CBDA
599	82.34	1.96	42.01020	High CBDA
600	79.55	1.94	41.00515	High CBDA
601	94.92	2.31	41.09091	High CBDA
602	53.86	1.46	36.89041	High CBDA
603	78.83	2.17	36.32719	High CBDA
604	80.19	2.26	35.48230	High CBDA
605	1.91	47.14	0.04052	High THCA
606	60.17	1.58	38.08228	High CBDA
607	32.13	0.97	33.12371	High CBDA
608	81.08	37.89	2.13988	Even Ratio
609	62.12	1.74	35.70115	High CBDA
610	72.91	2.24	32.54911	High CBDA
611	79.8	36.8	2.16848	Even Ratio
612	0.31	146.21	0.00212	High THCA
613	77.07	47.63	1.61810	Even Ratio
614	68.59	48.1	1.42599	Even Ratio
615	0.33	141.85	0.00233	High THCA
616	58.26	1.69	34.47337	High CBDA
617	78.76	36.6	2.15191	Even Ratio
618	61.3	1.68	36.48810	High CBDA
619	60.58	1.26	48.07937	High CBDA
620	0.36	136.93	0.00263	High THCA
621	60.89	2.3	26.47391	High CBDA
622	0.36	134.98	0.00267	High THCA
623	61.46	1.58	38.89873	High CBDA
624	0.39	136.58	0.00286	High THCA
625	63.94	1.91	33.47644	High CBDA
626	44.27	1.41	31.39716	High CBDA
627	74.54	45.16	1.65058	Even Ratio
628	70.87	2.04	34.74020	High CBDA
629	70.13	33.21	2.11171	Even Ratio
630	52.07	1.35	38.57037	High CBDA
631	62.05	1.72	36.07558	High CBDA
632	80.27	2.41	33.30705	High CBDA
633	41.81	1.33	31.43609	High CBDA
634	75.55	2.07	36.49758	High CBDA
635	76.94	36.94	2.08284	Even Ratio
636	51.6	1.48	34.86486	High CBDA

637	71.53	32.34	2.21181	Even Ratio
638	1.15	128.41	0.00896	High THCA
639	49.78	1.43	34.81119	High CBDA
640	0.79	164.48	0.00480	High THCA
641	65.31	2.27	28.77093	High CBDA
642	0.37	161.79	0.00229	High THCA
643	68.91	1.85	37.24865	High CBDA
644	47.89	1.42	33.72535	High CBDA
645	0.36	135.85	0.00265	High THCA
646	56.54	1.57	36.01274	High CBDA
647	68.02	48.38	1.40595	Even Ratio
648	51.97	1.48	35.11486	High CBDA
649	39.48	1.17	33.74359	High CBDA
650	77.26	2.21	34.95928	High CBDA
651	58.62	1.85	31.68649	High CBDA
652	82.46	37.98	2.17114	Even Ratio
653	61.56	3.06	20.11765	High CBDA
654	58.27	2.44	23.88115	High CBDA
655	58.9	0.77	76.49351	High CBDA
656	55.44	1.46	37.97260	High CBDA
657	72.64	51.93	1.39881	Even Ratio
658	0.45	143.23	0.00314	High THCA
659	69.68	2.26	30.83186	High CBDA
660	75.69	36.76	2.05903	Even Ratio
661	62.92	1.76	35.75000	High CBDA
662	0.7	151.74	0.00461	High THCA
663	51.73	1.38	37.48551	High CBDA
664	72.86	44.96	1.62055	Even Ratio
665	74.82	2.12	35.29245	High CBDA
666	72.88	51.01	1.42874	Even Ratio
667	0.47	126.59	0.00371	High THCA
668	50.04	1.27	39.40157	High CBDA
669	58.57	1.79	32.72067	High CBDA
670	77.61	48.61	1.59659	Even Ratio
671	72.05	1.92	37.52604	High CBDA
672	0.6	135.35	0.00443	High THCA
673	0.33	128.49	0.00257	High THCA
674	0.49	136.28	0.00360	High THCA
675	0.36	130.39	0.00276	High THCA
676	62.67	1.71	36.64912	High CBDA
677	72.94	2.19	33.30594	High CBDA
678	0.21	76.28	0.00275	High THCA
679	0.47	42.88	0.01096	High THCA
680	0.26	65.05	0.00400	High THCA
681	24.45	8.43	2.90036	Even Ratio
682	0.17	66.33	0.00256	High THCA

683	0.18	67.86	0.00265	High THCA
684	48.54	1.67	29.03143	High CBDA
685	70.89	2.32	30.52028	High CBDA
686	65.98	47.28	1.39563	Even Ratio
687	66.33	2.12	31.34842	High CBDA
688	0.37	133.22	0.00280	High THCA
689	63.74	45.70	1.39471	Even Ratio
690	67.64	2.13	31.69652	High CBDA
691	49.94	2.75	18.18566	High CBDA
692	51.38	1.63	31.57729	High CBDA
693	57.10	1.86	30.69544	High CBDA
694	48.50	1.58	30.72530	High CBDA
695	74.79	2.53	29.58815	High CBDA
696	43.09	1.40	30.68182	High CBDA
697	107.13	3.34	32.05719	High CBDA
698	74.14	2.43	30.50027	High CBDA
699	60.44	1.99	30.39605	High CBDA
700	41.98	1.36	30.89258	High CBDA
701	89.96	3.03	29.70688	High CBDA
702	46.24	1.44	32.01017	High CBDA
703	51.05	1.75	29.21480	High CBDA
704	0.60	120.42	0.00498	High THCA
705	68.68	2.10	32.73813	High CBDA
706	58.30	1.82	32.01712	High CBDA
707	57.97	1.84	31.46065	High CBDA
708	32.37	1.29	25.05811	High CBDA
709	56.20	1.76	32.00284	High CBDA
710	60.95	2.04	29.85122	High CBDA
711	59.09	1.89	31.30995	High CBDA
712	40.62	1.29	31.52531	High CBDA
713	39.95	1.39	28.78886	High CBDA
714	53.86	1.77	30.47047	High CBDA
715	0.39	121.25	0.00325	High THCA
716	41.05	1.50	27.43202	High CBDA
717	70.99	51.53	1.37778	Even Ratio
718	91.75	3.75	24.44594	High CBDA
719	0.36	123.02	0.00292	High THCA
720	68.63	50.07	1.37049	Even Ratio
721	71.84	2.54	28.23700	High CBDA
722	64.11	2.07	30.95520	High CBDA
723	60.18	2.11	28.57159	High CBDA
724	54.99	2.01	27.31365	High CBDA
725	58.32	1.89	30.79401	High CBDA
726	51.78	1.70	30.44904	High CBDA
727	43.86	1.42	30.94707	High CBDA
728	30.36	1.00	30.29361	High CBDA

729	75.26	2.43	30.97377	High CBDA
730	84.36	2.80	30.16078	High CBDA
731	42.93	1.63	26.30952	High CBDA
732	89.69	3.04	29.48351	High CBDA
733	44.22	1.55	28.55198	High CBDA
734	36.35	1.16	31.24010	High CBDA
