# Supplementary material for: Developing Prediction Models Using Near-Infrared Spectroscopy to Quantify Cannabinoid Content in Cannabis Sativa
Source: Sensors (Basel). 2023 Feb 27;23(5):2607. doi: 10.3390/s23052607 (PMC10007171; doi:10.3390/s23052607)
Supplement: Supplementary file 1 [file sensors-23-02607-s001.zip › sensors-2200076-supplementary/Table S4 LCMS MicroNIR Raw Data and Classifications for PLSDA.pdf]

Table S4: Definitions of even ratio, high CBDA and high THCA in relation to the CBDA and THCA ratio using LCMS quantitation data (n = 730) for MicroNIR.

List ID	CBDA (mg/g)	THCA (mg/g)	CBDA/THCA	Classification
1	35.84	14.15	2.53265	Even Ratio
2	0.29	73.53	0.00394	High THCA
3	0.39	102.92	0.00379	High THCA
4	0.24	72.84	0.00330	High THCA
5	0.33	91.69	0.00360	High THCA
6	42.58	15.54	2.74067	Even Ratio
7	56.02	20.06	2.79306	Even Ratio
8	54.79	20.07	2.73027	Even Ratio
9	0.22	45.71	0.00481	High THCA
10	77.99	39.11	1.99432	Even Ratio
11	47.99	18.49	2.59506	Even Ratio
12	0.54	116.61	0.00463	High THCA
13	59.95	23.41	2.56115	Even Ratio
14	61.23	28.44	2.15267	Even Ratio
15	26.41	1.24	21.22642	High CBDA
16	0.37	122.06	0.00303	High THCA
17	0.42	112.48	0.00373	High THCA
18	0.33	95.84	0.00344	High THCA
19	0.29	64.63	0.00449	High THCA
20	0.34	116.88	0.00291	High THCA
21	44.9	30.51	1.47166	Even Ratio
22	0.22	62.77	0.00350	High THCA
23	0.31	108.46	0.00286	High THCA
24	32.77	16.61	1.97250	Even Ratio
25	0.21	50.36	0.00417	High THCA
26	29.32	10.27	2.85508	Even Ratio
27	0.39	121.87	0.00320	High THCA
28	60.46	34.02	1.77744	Even Ratio
29	104.14	48.73	2.13709	Even Ratio
30	44.01	12.04	3.65577	Even Ratio
31	0.3	102.47	0.00293	High THCA
32	59.79	34.61	1.72735	Even Ratio
33	65.37	28.20	2.31836	Even Ratio
34	0.29	80.66	0.00360	High THCA
35	0.32	85.05	0.00376	High THCA
36	0.29	99.59	0.00291	High THCA
37	0.44	122.39	0.00360	High THCA

38	0.36	91.01	0.00396	High THCA
39	78.49	42.23	1.85873	Even Ratio
40	64.51	25.30	2.54991	Even Ratio
41	70.01	36.71	1.90717	Even Ratio
42	0.27	79.78	0.00338	High THCA
43	35.64	16.55	2.15383	Even Ratio
44	75.09	30.89	2.43082	Even Ratio
45	0.34	104.35	0.00326	High THCA
46	53	25.52	2.07701	Even Ratio
47	0.41	138.30	0.00296	High THCA
48	0.34	99.32	0.00342	High THCA
49	45.82	17.65	2.59574	Even Ratio
50	0.29	104.90	0.00276	High THCA
51	0.35	131.31	0.00267	High THCA
52	0.35	88.88	0.00394	High THCA
53	43.57	11.54	3.77416	Even Ratio
54	0.46	170.33	0.00270	High THCA
55	0.34	115.10	0.00295	High THCA
56	60.95	23.72	2.56929	Even Ratio
57	0.31	63.68	0.00487	High THCA
58	0.25	104.92	0.00238	High THCA
59	57.71	21.33	2.70580	Even Ratio
60	23.98	1.12	21.48768	High CBDA
61	0.27	93.09	0.00290	High THCA
62	0.32	104.60	0.00306	High THCA
63	0.28	57.54	0.00487	High THCA
64	0.23	63.65	0.00361	High THCA
65	0.28	95.56	0.00293	High THCA
66	0.37	129.70	0.00285	High THCA
67	57.25	15.80	3.62250	Even Ratio
68	65.8	24.99	2.63302	Even Ratio
69	0.35	82.19	0.00426	High THCA
70	0.4	143.39	0.00279	High THCA
71	47.8	25.75	1.85613	Even Ratio
72	0.3	102.91	0.00292	High THCA
73	50.71	15.34	3.30674	Even Ratio
74	0.35	109.02	0.00321	High THCA
75	0.35	120.99	0.00289	High THCA
76	74.26	28.32	2.62205	Even Ratio
77	0.21	65.50	0.00321	High THCA
78	0.3	73.25	0.00410	High THCA
79	0.35	112.87	0.00310	High THCA
80	0.24	73.56	0.00326	High THCA
81	0.29	89.78	0.00323	High THCA
82	0.3	101.69	0.00295	High THCA
83	0.3	83.00	0.00361	High THCA

84	61.42	24.33	2.52484	Even Ratio
85	27.44	12.62	2.17428	Even Ratio
86	0.4	123.99	0.00323	High THCA
87	0.34	86.26	0.00394	High THCA
88	0.27	83.37	0.00324	High THCA
89	37	16.12	2.29527	Even Ratio
90	0.34	112.97	0.00301	High THCA
91	0.29	76.69	0.00378	High THCA
92	66.55	22.46	2.96303	Even Ratio
93	46.07	16.66	2.76585	Even Ratio
94	46.52	18.23	2.55128	Even Ratio
95	0.33	98.22	0.00336	High THCA
96	0.32	91.88	0.00348	High THCA
97	0.33	79.09	0.00417	High THCA
98	68.09	29.69	2.29301	Even Ratio
99	0.25	48.09	0.00520	High THCA
100	0.27	62.97	0.00429	High THCA
101	65.39	24.44	2.67563	Even Ratio
102	38.71	22.72	1.70373	Even Ratio
103	0.33	104.28	0.00316	High THCA
104	45.89	12.07	3.80297	Even Ratio
105	0.37	112.73	0.00328	High THCA
106	0.32	91.91	0.00348	High THCA
107	0.29	53.92	0.00538	High THCA
108	0.28	67.14	0.00417	High THCA
109	50.72	20.10	2.52373	Even Ratio
110	52.13	25.85	2.01653	Even Ratio
111	0.38	103.12	0.00369	High THCA
112	29.78	13.18	2.26030	Even Ratio
113	0.61	84.76	0.00720	High THCA
114	0.31	63.85	0.00486	High THCA
115	71.69	31.17	2.30008	Even Ratio
116	0.27	70.22	0.00385	High THCA
117	80.9	46.04	1.75730	Even Ratio
118	0.39	94.79	0.00411	High THCA
119	59.27	24.71	2.39831	Even Ratio
120	85.89	36.70	2.34020	Even Ratio
121	0.28	97.62	0.00287	High THCA
122	60.11	22.52	2.66892	Even Ratio
123	65.98	21.15	3.12011	Even Ratio
124	70.02	28.43	2.46248	Even Ratio
125	64.14	21.03	3.04925	Even Ratio
126	0.37	107.38	0.00345	High THCA
127	59.34	21.06	2.81714	Even Ratio
128	0.29	86.58	0.00335	High THCA
129	0.26	71.79	0.00362	High THCA

130	39.66	12.87	3.08109	Even Ratio
131	0.46	134.40	0.00342	High THCA
132	34.2	1.27	26.90876	High CBDA
133	51.88	34.44	1.50643	Even Ratio
134	63.67	30.13	2.11330	Even Ratio
135	0.3	61.65	0.00487	High THCA
136	54.62	21.56	2.53300	Even Ratio
137	41.41	10.48	3.94972	Even Ratio
138	0.31	114.98	0.00270	High THCA
139	0.31	90.24	0.00344	High THCA
140	0.32	127.80	0.00250	High THCA
141	0.33	93.17	0.00354	High THCA
142	0.27	69.41	0.00389	High THCA
143	76	32.81	2.31627	Even Ratio
144	89.95	43.00	2.09177	Even Ratio
145	0.24	78.91	0.00304	High THCA
146	60.99	24.08	2.53305	Even Ratio
147	43.8	13.17	3.32566	Even Ratio
148	0.52	73.77	0.00705	High THCA
149	40.17	19.82	2.02642	Even Ratio
150	0.65	117.26	0.00554	High THCA
151	0.27	89.13	0.00303	High THCA
152	49	18.63	2.63008	Even Ratio
153	0.33	123.60	0.00267	High THCA
154	0.38	128.09	0.00297	High THCA
155	0.34	91.25	0.00373	High THCA
156	84.82	43.39	1.95472	Even Ratio
157	71.94	30.25	2.37840	Even Ratio
158	0.36	100.77	0.00357	High THCA
159	59.16	22.74	2.60159	Even Ratio
160	40.84	20.47	1.99544	Even Ratio
161	0.43	97.82	0.00440	High THCA
162	0.31	86.34	0.00359	High THCA
163	0.42	121.88	0.00345	High THCA
164	53.64	31.53	1.70106	Even Ratio
165	0.38	99.83	0.00381	High THCA
166	57.55	19.15	3.00469	Even Ratio
167	0.35	81.32	0.00430	High THCA
168	65.97	23.90	2.76001	Even Ratio
169	0.36	72.24	0.00498	High THCA
170	35.19	14.96	2.35171	Even Ratio
171	0.32	54.60	0.00586	High THCA
172	71.18	31.67	2.24741	Even Ratio
173	56.71	20.59	2.75361	Even Ratio
174	0.31	67.90	0.00457	High THCA
175	0.35	74.23	0.00472	High THCA

176	0.34	99.53	0.00342	High THCA
177	71.47	46.99	1.52084	Even Ratio
178	43.44	20.50	2.11861	Even Ratio
179	0.53	98.10	0.00540	High THCA
180	69.33	24.04	2.88352	Even Ratio
181	0.35	107.55	0.00325	High THCA
182	0.3	75.59	0.00397	High THCA
183	62.44	22.52	2.77247	Even Ratio
184	44.41	14.37	3.09036	Even Ratio
185	0.54	93.88	0.00575	High THCA
186	17.48	6.08	2.87431	Even Ratio
187	0.29	83.82	0.00346	High THCA
188	41.71	15.02	2.77703	Even Ratio
189	0.32	97.04	0.00330	High THCA
190	80.48	44.23	1.81977	Even Ratio
191	0.34	110.83	0.00307	High THCA
192	0.28	64.25	0.00436	High THCA
193	0.28	57.66	0.00486	High THCA
194	0.32	89.08	0.00359	High THCA
195	0.26	84.07	0.00309	High THCA
196	0.28	95.81	0.00292	High THCA
197	70.83	32.37	2.18843	Even Ratio
198	47.45	16.11	2.94561	Even Ratio
199	0.32	83.56	0.00383	High THCA
200	67.2	26.03	2.58131	Even Ratio
201	0.42	79.72	0.00527	High THCA
202	0.27	87.46	0.00309	High THCA
203	0.39	125.32	0.00311	High THCA
204	28.18	12.70	2.21869	Even Ratio
205	9.83	0.66	14.83663	High CBDA
206	0.28	100.57	0.00278	High THCA
207	0.27	76.07	0.00355	High THCA
208	22.53	9.97	2.25996	Even Ratio
209	0.34	118.30	0.00287	High THCA
210	0.33	110.49	0.00299	High THCA
211	0.33	69.98	0.00472	High THCA
212	0.36	88.46	0.00407	High THCA
213	78.59	35.65	2.20455	Even Ratio
214	0.32	97.63	0.00328	High THCA
215	0.31	101.53	0.00305	High THCA
216	70.33	47.10	1.49310	Even Ratio
217	0.34	93.50	0.00364	High THCA
218	0.34	79.96	0.00425	High THCA
219	37.5	17.34	2.16308	Even Ratio
220	38.37	13.27	2.89067	Even Ratio
221	51.67	24.59	2.10149	Even Ratio

222	72.4	28.88	2.50719	Even Ratio
223	60.98	31.24	1.95195	Even Ratio
224	0.37	110.68	0.00334	High THCA
225	0.4	102.64	0.00390	High THCA
226	0.24	67.45	0.00356	High THCA
227	60.52	25.57	2.36722	Even Ratio
228	0.29	83.54	0.00347	High THCA
229	0.43	97.66	0.00440	High THCA
230	0.3	96.96	0.00309	High THCA
231	60.06	31.10	1.93094	Even Ratio
232	0.29	73.89	0.00392	High THCA
233	0.31	86.79	0.00357	High THCA
234	0.31	102.85	0.00301	High THCA
235	54.22	20.74	2.61394	Even Ratio
236	0.26	80.77	0.00322	High THCA
237	69.96	34.72	2.01523	Even Ratio
238	63.63	26.91	2.36446	Even Ratio
239	0.29	97.69	0.00297	High THCA
240	54.94	22.95	2.39375	Even Ratio
241	0.31	109.12	0.00284	High THCA
242	0.23	70.69	0.00325	High THCA
243	0.23	77.18	0.00298	High THCA
244	0.25	104.82	0.00238	High THCA
245	0.21	73.63	0.00285	High THCA
246	0.23	79.30	0.00290	High THCA
247	0.35	107.77	0.00325	High THCA
248	0.27	59.86	0.00451	High THCA
249	0.24	66.15	0.00363	High THCA
250	0.31	91.29	0.00340	High THCA
251	29.97	13.86	2.16260	Even Ratio
252	29.42	14.74	1.99642	Even Ratio
253	74.36	25.60	2.90417	Even Ratio
254	0.32	75.36	0.00425	High THCA
255	66.39	22.77	2.91574	Even Ratio
256	0.38	122.45	0.00310	High THCA
257	68.68	29.08	2.36188	Even Ratio
258	51.23	16.10	3.18282	Even Ratio
259	0.53	99.69	0.00532	High THCA
260	42.64	15.96	2.67125	Even Ratio
261	0.34	77.21	0.00440	High THCA
262	47.27	11.43	4.13604	Even Ratio
263	0.27	85.76	0.00315	High THCA
264	0.37	95.29	0.00388	High THCA
265	0.33	110.03	0.00300	High THCA
266	40.74	14.85	2.74301	Even Ratio
267	66.62	28.68	2.32292	Even Ratio

268	72.1	23.24	3.10221	Even Ratio
269	0.4	92.83	0.00431	High THCA
270	0.3	89.63	0.00335	High THCA
271	62.91	32.49	1.93637	Even Ratio
272	66.29	25.98	2.55109	Even Ratio
273	0.34	97.11	0.00350	High THCA
274	0.29	85.75	0.00338	High THCA
275	0.46	87.37	0.00526	High THCA
276	0.22	72.30	0.00304	High THCA
277	0.29	102.99	0.00282	High THCA
278	0.25	85.53	0.00292	High THCA
279	0.34	131.32	0.00259	High THCA
280	0.45	131.66	0.00342	High THCA
281	0.39	108.73	0.00359	High THCA
282	53.95	26.66	2.02394	Even Ratio
283	0.29	104.22	0.00278	High THCA
284	72.64	21.60	3.36352	Even Ratio
285	50.1	23.66	2.11708	Even Ratio
286	0.27	78.51	0.00344	High THCA
287	0.35	94.29	0.00371	High THCA
288	0.26	79.57	0.00327	High THCA
289	48.99	26.95	1.81749	Even Ratio
290	57.63	22.73	2.53558	Even Ratio
291	0.3	52.00	0.00577	High THCA
292	0.33	92.86	0.00355	High THCA
293	0.25	56.85	0.00440	High THCA
294	48.28	19.17	2.51890	Even Ratio
295	0.29	77.13	0.00376	High THCA
296	0.4	114.13	0.00350	High THCA
297	0.28	62.52	0.00448	High THCA
298	64.44	20.73	3.10910	Even Ratio
299	62.79	27.71	2.26576	Even Ratio
300	76.12	34.14	2.22948	Even Ratio
301	63.75	28.62	2.22719	Even Ratio
302	0.35	87.38	0.00401	High THCA
303	0.3	97.60	0.00307	High THCA
304	0.34	100.94	0.00337	High THCA
305	0.33	116.64	0.00283	High THCA
306	0.29	81.36	0.00356	High THCA
307	0.3	88.95	0.00337	High THCA
308	0.23	59.07	0.00389	High THCA
309	0.36	101.51	0.00355	High THCA
310	53.84	18.86	2.85411	Even Ratio
311	66.76	30.22	2.20890	Even Ratio
312	58.91	28.15	2.09264	Even Ratio
313	0.31	122.31	0.00253	High THCA

314	0.35	125.36	0.00279	High THCA
315	17.14	0.82	20.94682	High CBDA
316	43.87	17.72	2.47613	Even Ratio
317	65.03	34.05	1.90998	Even Ratio
318	0.31	64.20	0.00483	High THCA
319	66.71	33.53	1.98956	Even Ratio
320	0.3	82.11	0.00365	High THCA
321	0.38	118.54	0.00321	High THCA
322	0.25	80.47	0.00311	High THCA
323	79.39	32.69	2.42868	Even Ratio
324	0.36	73.01	0.00493	High THCA
325	0.34	91.94	0.00370	High THCA
326	27.12	9.92	2.73268	Even Ratio
327	0.31	71.17	0.00436	High THCA
328	0.24	69.00	0.00348	High THCA
329	74.14	21.70	3.41643	Even Ratio
330	0.32	85.89	0.00373	High THCA
331	66.67	27.68	2.40836	Even Ratio
332	60.34	25.94	2.32622	Even Ratio
333	0.28	72.26	0.00387	High THCA
334	61.77	31.12	1.98505	Even Ratio
335	0.26	69.28	0.00375	High THCA
336	0.36	92.41	0.00390	High THCA
337	0.25	78.11	0.00320	High THCA
338	0.28	71.67	0.00391	High THCA
339	0.22	53.62	0.00410	High THCA
340	13.26	6.09	2.17695	Even Ratio
341	0.39	111.64	0.00349	High THCA
342	65.87	24.00	2.74474	Even Ratio
343	0.36	88.33	0.00408	High THCA
344	0.24	65.34	0.00367	High THCA
345	0.22	63.24	0.00348	High THCA
346	0.25	80.90	0.00309	High THCA
347	0.38	118.31	0.00321	High THCA
348	0.33	114.33	0.00289	High THCA
349	0.25	74.87	0.00334	High THCA
350	0.23	68.71	0.00335	High THCA
351	0.27	75.32	0.00358	High THCA
352	0.27	83.59	0.00323	High THCA
353	29.81	12.06	2.47119	Even Ratio
354	0.28	105.39	0.00266	High THCA
355	0.23	69.91	0.00329	High THCA
356	0.29	102.84	0.00282	High THCA
357	0.34	109.27	0.00311	High THCA
358	0.28	102.96	0.00272	High THCA
359	0.32	103.87	0.00308	High THCA

360	0.29	81.96	0.00354	High THCA
361	0.28	91.49	0.00306	High THCA
362	49.4	14.12	3.49810	Even Ratio
363	0.32	94.36	0.00339	High THCA
364	0.3	61.16	0.00491	High THCA
365	62.18	38.65	1.60895	Even Ratio
366	14.76	6.25	2.36205	Even Ratio
367	1.23	56.33	0.02184	High THCA
368	0.31	99.94	0.00310	High THCA
369	0.29	105.75	0.00274	High THCA
370	0.35	125.68	0.00278	High THCA
371	0.27	83.39	0.00324	High THCA
372	0.27	85.08	0.00317	High THCA
373	0.35	148.84	0.00235	High THCA
374	0.36	112.20	0.00321	High THCA
375	0.28	106.31	0.00263	High THCA
376	60.5	32.84	1.84240	Even Ratio
377	0.31	99.37	0.00312	High THCA
378	83.37	40.04	2.08233	Even Ratio
379	0.38	136.29	0.00279	High THCA
380	0.36	114.91	0.00313	High THCA
381	62.43	30.96	2.01617	Even Ratio
382	0.33	95.89	0.00344	High THCA
383	0.3	100.10	0.00300	High THCA
384	44.71	31.39	1.42416	Even Ratio
385	0.33	99.25	0.00332	High THCA
386	38.49	14.19	2.71190	Even Ratio
387	0.33	130.84	0.00252	High THCA
388	75.01	24.58	3.05183	Even Ratio
389	19.83	9.97	1.98849	Even Ratio
390	0.33	81.97	0.00403	High THCA
391	0.23	43.98	0.00523	High THCA
392	0.25	81.23	0.00308	High THCA
393	29.07	12.04	2.41486	Even Ratio
394	0.23	73.92	0.00311	High THCA
395	0.33	88.30	0.00374	High THCA
396	0.29	89.31	0.00325	High THCA
397	0.21	68.89	0.00305	High THCA
398	0.28	95.33	0.00294	High THCA
399	0.33	103.13	0.00320	High THCA
400	0.27	84.58	0.00319	High THCA
401	0.32	118.53	0.00270	High THCA
402	66.59	23.57	2.82517	Even Ratio
403	67.4	18.40	3.66338	Even Ratio
404	68.11	26.48	2.57243	Even Ratio
405	0.42	92.22	0.00455	High THCA

406	73.01	22.18	3.29115	Even Ratio
407	76.43	35.16	2.17355	Even Ratio
408	0.4	85.32	0.00469	High THCA
409	0.31	75.91	0.00408	High THCA
410	40.88	20.43	2.00145	Even Ratio
411	54.85	15.37	3.56802	Even Ratio
412	0.5	87.77	0.00570	High THCA
413	47.31	16.49	2.86951	Even Ratio
414	0.28	69.90	0.00401	High THCA
415	0.3	108.82	0.00276	High THCA
416	0.45	117.71	0.00382	High THCA
417	0.27	88.67	0.00304	High THCA
418	59.97	20.53	2.92160	Even Ratio
419	92.26	46.57	1.98117	Even Ratio
420	0.23	60.99	0.00377	High THCA
421	0.33	40.90	0.00807	High THCA
422	44.42	16.83	2.63899	Even Ratio
423	42.91	13.37	3.20867	Even Ratio
424	0.59	150.49	0.00392	High THCA
425	55.98	41.38	1.35280	Even Ratio
426	0.3	91.64	0.00327	High THCA
427	41.38	16.88	2.45179	Even Ratio
428	0.24	46.78	0.00513	High THCA
429	0.33	117.53	0.00281	High THCA
430	0.3	92.26	0.00325	High THCA
431	0.24	62.65	0.00383	High THCA
432	61.56	23.75	2.59244	Even Ratio
433	36.45	16.44	2.21755	Even Ratio
434	0.31	102.36	0.00303	High THCA
435	59.41	19.33	3.07278	Even Ratio
436	0.27	48.31	0.00559	High THCA
437	0.33	121.46	0.00272	High THCA
438	0.27	90.62	0.00298	High THCA
439	50.69	15.43	3.28552	Even Ratio
440	0.3	87.87	0.00341	High THCA
441	0.28	101.22	0.00277	High THCA
442	0.31	111.40	0.00278	High THCA
443	0.28	81.68	0.00343	High THCA
444	0.31	92.13	0.00336	High THCA
445	0.28	84.30	0.00332	High THCA
446	0.24	82.38	0.00291	High THCA
447	58	19.21	3.01968	Even Ratio
448	0.28	88.28	0.00317	High THCA
449	0.45	136.74	0.00329	High THCA
450	62.48	23.14	2.69989	Even Ratio
451	0.45	100.94	0.00446	High THCA

452	0.29	122.63	0.00236	High THCA
453	0.33	134.97	0.00245	High THCA
454	0.4	133.06	0.00301	High THCA
455	0.29	100.99	0.00287	High THCA
456	0.4	141.30	0.00283	High THCA
457	0.26	63.01	0.00413	High THCA
458	0.26	40.25	0.00646	High THCA
459	0.32	82.85	0.00386	High THCA
460	0.28	81.98	0.00342	High THCA
461	74.41	37.20	2.00022	Even Ratio
462	33.62	13.43	2.50377	Even Ratio
463	0.27	80.22	0.00337	High THCA
464	0.22	51.86	0.00424	High THCA
465	0.47	129.67	0.00362	High THCA
466	0.26	77.28	0.00336	High THCA
467	67.64	26.67	2.53588	Even Ratio
468	0.31	93.16	0.00333	High THCA
469	0.26	61.71	0.00421	High THCA
470	66.52	31.39	2.11937	Even Ratio
471	0.33	101.96	0.00324	High THCA
472	0.29	84.47	0.00343	High THCA
473	29.95	8.41	3.56084	Even Ratio
474	0.21	32.18	0.00653	High THCA
475	62.4	29.44	2.11966	Even Ratio
476	87.88	2.22	39.58559	High CBDA
477	69	1.81	38.12155	High CBDA
478	56.06	1.69	33.17160	High CBDA
479	69.53	1.75	39.73143	High CBDA
480	70.08	1.86	37.67742	High CBDA
481	86.38	2.39	36.14226	High CBDA
482	58.27	1.31	44.48092	High CBDA
483	74.57	1.51	49.38411	High CBDA
484	94.61	44.64	2.11940	Even Ratio
485	87.46	2.12	41.25472	High CBDA
486	97.95	3.09	31.69903	High CBDA
487	64.72	1.9	34.06316	High CBDA
488	71.12	1.22	58.29508	High CBDA
489	99.69	2.42	41.19421	High CBDA
490	113.97	2.52	45.22619	High CBDA
491	77.35	41.46	1.86565	Even Ratio
492	69.44	1.48	46.91892	High CBDA
493	51.71	1.43	36.16084	High CBDA
494	81.15	1.98	40.98485	High CBDA
495	96.87	1.95	49.67692	High CBDA
496	101.91	2.58	39.50000	High CBDA
497	67.54	1.57	43.01911	High CBDA

498	62.34	1.51	41.28477	High CBDA
499	86.93	1.76	49.39205	High CBDA
500	74.83	2.21	33.85973	High CBDA
501	60.73	1.12	54.22321	High CBDA
502	97.32	2.28	42.68421	High CBDA
503	122.48	51.64	2.37180	Even Ratio
504	76.39	1.33	57.43609	High CBDA
505	75.31	1.96	38.42347	High CBDA
506	68.89	1.99	34.61809	High CBDA
507	109.6	2.75	39.85455	High CBDA
508	52.24	1.3	40.18462	High CBDA
509	71.43	1.24	57.60484	High CBDA
510	77.84	1.87	41.62567	High CBDA
511	75.46	1.72	43.87209	High CBDA
512	89.16	2.22	40.16216	High CBDA
513	93.84	2.43	38.61728	High CBDA
514	80.51	1.99	40.45729	High CBDA
515	64.97	1.39	46.74101	High CBDA
516	85.39	47.9	1.78267	Even Ratio
517	58.88	1.54	38.23377	High CBDA
518	84.54	1.54	54.89610	High CBDA
519	92.81	2.64	35.15530	High CBDA
520	101.25	2.47	40.99190	High CBDA
521	61.39	1.29	47.58915	High CBDA
522	0.72	114.56	0.00628	High THCA
523	69.76	1.7	41.03529	High CBDA
524	56.26	1.26	44.65079	High CBDA
525	73.02	1.78	41.02247	High CBDA
526	87.62	1.9	46.11579	High CBDA
527	88.42	1.9	46.53684	High CBDA
528	83.36	1.65	50.52121	High CBDA
529	85.9	2.62	32.78626	High CBDA
530	88.76	2.48	35.79032	High CBDA
531	92.04	2.77	33.22744	High CBDA
532	63.81	1.59	40.13208	High CBDA
533	73.19	37.4	1.95695	Even Ratio
534	58.03	1.65	35.16970	High CBDA
535	0.33	113.27	0.00291	High THCA
536	89.98	2.44	36.87705	High CBDA
537	84.7	2.04	41.51961	High CBDA
538	71.04	1.43	49.67832	High CBDA
539	63.85	1.6	39.90625	High CBDA
540	120.4	3.6	33.44444	High CBDA
541	68.78	1.36	50.57353	High CBDA
542	50.98	0.93	54.81720	High CBDA
543	102.54	42.39	2.41897	Even Ratio

544	79.48	1.82	43.67033	High CBDA
545	80.44	2.25	35.75111	High CBDA
546	87.29	3.31	26.37160	High CBDA
547	59.31	1.23	48.21951	High CBDA
548	96.47	2.77	34.82671	High CBDA
549	75.11	1.83	41.04372	High CBDA
550	102.46	42.43	2.41480	Even Ratio
551	54	1.15	46.95652	High CBDA
552	69.54	1.66	41.89157	High CBDA
553	73.07	1.53	47.75817	High CBDA
554	48.47	1.01	47.99010	High CBDA
555	72.5	1.83	39.61749	High CBDA
556	64.79	1.33	48.71429	High CBDA
557	88.06	2.4	36.69167	High CBDA
558	121.15	2.88	42.06597	High CBDA
559	62.49	1.73	36.12139	High CBDA
560	77.73	1.55	50.14839	High CBDA
561	66.13	1.53	43.22222	High CBDA
562	82.91	2.02	41.04455	High CBDA
563	65.34	1.45	45.06207	High CBDA
564	56.24	1.25	44.99200	High CBDA
565	78.54	1.96	40.07143	High CBDA
566	0.6	117.11	0.00512	High THCA
567	83.17	2.15	38.68372	High CBDA
568	94.58	43.87	2.15592	Even Ratio
569	97.92	2.37	41.31646	High CBDA
570	56.23	1.78	31.58989	High CBDA
571	87.12	1.9	45.85263	High CBDA
572	68.01	1.83	37.16393	High CBDA
573	77.24	2.25	34.32889	High CBDA
574	84.21	1.79	47.04469	High CBDA
575	53.01	1.22	43.45082	High CBDA
576	50.12	1.13	44.35398	High CBDA
577	67.04	1.9	35.28421	High CBDA
578	76.31	1.66	45.96988	High CBDA
579	87.51	2.23	39.24215	High CBDA
580	53.11	1.19	44.63025	High CBDA
581	59.32	1.61	36.84472	High CBDA
582	67.88	1.75	38.78857	High CBDA
583	90.77	35.04	2.59047	Even Ratio
584	70.52	2.21	31.90950	High CBDA
585	67.26	1.6	42.03750	High CBDA
586	116.9	55.71	2.09837	Even Ratio
587	86.08	2.04	42.19608	High CBDA
588	79.7	1.74	45.80460	High CBDA
589	79.37	2.45	32.39592	High CBDA

590	79.48	2.07	38.39614	High CBDA
591	0.26	89.01	0.00292	High THCA
592	79.11	1.78	44.44382	High CBDA
593	73.02	2.08	35.10577	High CBDA
594	66.2	1.64	40.36585	High CBDA
595	82.34	1.96	42.01020	High CBDA
596	79.55	1.94	41.00515	High CBDA
597	94.92	2.31	41.09091	High CBDA
598	53.86	1.46	36.89041	High CBDA
599	78.83	2.17	36.32719	High CBDA
600	80.19	2.26	35.48230	High CBDA
601	1.91	47.14	0.04052	High THCA
602	60.17	1.58	38.08228	High CBDA
603	32.13	0.97	33.12371	High CBDA
604	81.08	37.89	2.13988	Even Ratio
605	62.12	1.74	35.70115	High CBDA
606	72.91	2.24	32.54911	High CBDA
607	79.8	36.8	2.16848	Even Ratio
608	0.31	146.21	0.00212	High THCA
609	77.07	47.63	1.61810	Even Ratio
610	68.59	48.1	1.42599	Even Ratio
611	0.33	141.85	0.00233	High THCA
612	58.26	1.69	34.47337	High CBDA
613	78.76	36.6	2.15191	Even Ratio
614	61.3	1.68	36.48810	High CBDA
615	60.58	1.26	48.07937	High CBDA
616	0.36	136.93	0.00263	High THCA
617	60.89	2.3	26.47391	High CBDA
618	0.36	134.98	0.00267	High THCA
619	61.46	1.58	38.89873	High CBDA
620	0.39	136.58	0.00286	High THCA
621	63.94	1.91	33.47644	High CBDA
622	44.27	1.41	31.39716	High CBDA
623	74.54	45.16	1.65058	Even Ratio
624	70.87	2.04	34.74020	High CBDA
625	70.13	33.21	2.11171	Even Ratio
626	52.07	1.35	38.57037	High CBDA
627	62.05	1.72	36.07558	High CBDA
628	80.27	2.41	33.30705	High CBDA
629	41.81	1.33	31.43609	High CBDA
630	75.55	2.07	36.49758	High CBDA
631	76.94	36.94	2.08284	Even Ratio
632	51.6	1.48	34.86486	High CBDA
633	71.53	32.34	2.21181	Even Ratio

634	1.15	128.41	0.00896	High THCA
635	49.78	1.43	34.81119	High CBDA
636	0.79	164.48	0.00480	High THCA
637	65.31	2.27	28.77093	High CBDA
638	0.37	161.79	0.00229	High THCA
639	68.91	1.85	37.24865	High CBDA
640	47.89	1.42	33.72535	High CBDA
641	0.36	135.85	0.00265	High THCA
642	56.54	1.57	36.01274	High CBDA
643	68.02	48.38	1.40595	Even Ratio
644	51.97	1.48	35.11486	High CBDA
645	39.48	1.17	33.74359	High CBDA
646	77.26	2.21	34.95928	High CBDA
647	58.62	1.85	31.68649	High CBDA
648	82.46	37.98	2.17114	Even Ratio
649	61.56	3.06	20.11765	High CBDA
650	58.27	2.44	23.88115	High CBDA
651	58.9	0.77	76.49351	High CBDA
652	55.44	1.46	37.97260	High CBDA
653	72.64	51.93	1.39881	Even Ratio
654	0.45	143.23	0.00314	High THCA
655	69.68	2.26	30.83186	High CBDA
656	75.69	36.76	2.05903	Even Ratio
657	62.92	1.76	35.75000	High CBDA
658	0.7	151.74	0.00461	High THCA
659	51.73	1.38	37.48551	High CBDA
660	72.86	44.96	1.62055	Even Ratio
661	74.82	2.12	35.29245	High CBDA
662	72.88	51.01	1.42874	Even Ratio
663	0.47	126.59	0.00371	High THCA
664	50.04	1.27	39.40157	High CBDA
665	58.57	1.79	32.72067	High CBDA
666	77.61	48.61	1.59659	Even Ratio
667	72.05	1.92	37.52604	High CBDA
668	0.6	135.35	0.00443	High THCA
669	0.33	128.49	0.00257	High THCA
670	0.49	136.28	0.00360	High THCA
671	0.36	130.39	0.00276	High THCA
672	62.67	1.71	36.64912	High CBDA
673	72.94	2.19	33.30594	High CBDA
674	0.21	76.28	0.00275	High THCA
675	0.47	42.88	0.01096	High THCA
676	0.26	65.05	0.00400	High THCA
677	24.45	8.43	2.90036	Even Ratio

678	0.17	66.33	0.00256	High THCA
679	0.18	67.86	0.00265	High THCA
680	48.54	1.67	29.03143	High CBDA
681	70.89	2.32	30.52028	High CBDA
682	65.98	47.28	1.39563	Even Ratio
683	66.33	2.12	31.34842	High CBDA
684	0.37	133.22	0.00280	High THCA
685	63.74	45.70	1.39471	Even Ratio
686	67.64	2.13	31.69652	High CBDA
687	49.94	2.75	18.18566	High CBDA
688	51.38	1.63	31.57729	High CBDA
689	57.10	1.86	30.69544	High CBDA
690	48.50	1.58	30.72530	High CBDA
691	74.79	2.53	29.58815	High CBDA
692	43.09	1.40	30.68182	High CBDA
693	107.13	3.34	32.05719	High CBDA
694	74.14	2.43	30.50027	High CBDA
695	60.44	1.99	30.39605	High CBDA
696	41.98	1.36	30.89258	High CBDA
697	89.96	3.03	29.70688	High CBDA
698	46.24	1.44	32.01017	High CBDA
699	51.05	1.75	29.21480	High CBDA
700	0.60	120.42	0.00498	High THCA
701	68.68	2.10	32.73813	High CBDA
702	58.30	1.82	32.01712	High CBDA
703	57.97	1.84	31.46065	High CBDA
704	32.37	1.29	25.05811	High CBDA
705	56.20	1.76	32.00284	High CBDA
706	60.95	2.04	29.85122	High CBDA
707	59.09	1.89	31.30995	High CBDA
708	40.62	1.29	31.52531	High CBDA
709	39.95	1.39	28.78886	High CBDA
710	53.86	1.77	30.47047	High CBDA
711	0.39	121.25	0.00325	High THCA
712	41.05	1.50	27.43202	High CBDA
713	70.99	51.53	1.37778	Even Ratio
714	91.75	3.75	24.44594	High CBDA
715	0.36	123.02	0.00292	High THCA
716	68.63	50.07	1.37049	Even Ratio
717	71.84	2.54	28.23700	High CBDA
718	64.11	2.07	30.95520	High CBDA
719	60.18	2.11	28.57159	High CBDA
720	54.99	2.01	27.31365	High CBDA
721	58.32	1.89	30.79401	High CBDA
722	51.78	1.70	30.44904	High CBDA
723	43.86	1.42	30.94707	High CBDA

724	30.36	1.00	30.29361	High CBDA
725	75.26	2.43	30.97377	High CBDA
726	84.36	2.80	30.16078	High CBDA
727	42.93	1.63	26.30952	High CBDA
728	89.69	3.04	29.48351	High CBDA
729	44.22	1.55	28.55198	High CBDA
730	36.35	1.16	31.24010	High CBDA
