# Supplementary material for: Developing Prediction Models Using Near-Infrared Spectroscopy to Quantify Cannabinoid Content in Cannabis Sativa
Source: Sensors (Basel). 2023 Feb 27;23(5):2607. doi: 10.3390/s23052607 (PMC10007171; doi:10.3390/s23052607)
Supplement: Supplementary file 1 [file sensors-23-02607-s001.zip › sensors-2200076-supplementary/Table S5 Cannabinoid LCMS Standard Error Laboratory Values.pdf]

Table S5: Standard error laboratory values of cannabinoid dataset.

Table S5.1 Standard Error Laboratory (SEL) of cannabinoids according to reference LCMS data of all harvest groups. (n = 734)

Cannabinoid	SEL (mg/g)
**CBDA**	1.24
**CBD**	0.96
**CBN**	0.08
**THC**	0.79
**CBC**	0.08
**THCA-A**	4.45
**CBDVA**	0.07
**CBDV**	0.11
**CBGA**	0.79
**CBG**	0.17
**THCV**	0.06
**THCVA**	0.56
**CBNA**	0.13
**CBCA**	0.61

Table S5.2 Standard Error Laboratory (SEL) of cannabinoids according to reference LCMS data of harvest group 1. (n = 479)

Cannabinoid	SEL (mg/g)
**CBDA**	2.21
**CBD**	0.32
**CBN**	0.05
**THC**	0.84
**CBC**	0.06
**THCA-A**	3.68
**CBDVA**	0.07
**CBDV**	0.04
**CBGA**	0.36
**CBG**	0.08
**THCV**	0.01
**THCVA**	0.16
**CBNA**	0.12
**CBCA**	0.38

Table S5.3 Standard Error Laboratory versus the Standard Error Prediction for CBDA in the high (n = 406) and low (n = 328) ranges.

**Bruker**	**SEL (mg/g)**	**SEP (mg/g)**	**SEP/SEL**	**R^2**	**Mean Cannabinoid Concentration from LCMS data (mg/g)**
CBDA High range	1.56	4.86	**3.11**	0.91	63.80
CBDA Low range	0.36	0.66	**1.82**	0.25	0.49
**MicroNIR**					
CBDA High range	1.56	5.15	**3.30**	0.89	63.80
CBDA Low range	0.36	0.55	**1.50**	0.21	0.49

Low range: 0 – 8 mg/g; High range: 8 – 90 mg/g
