# Supplementary material for: Developing Prediction Models Using Near-Infrared Spectroscopy to Quantify Cannabinoid Content in Cannabis Sativa
Source: Sensors (Basel). 2023 Feb 27;23(5):2607. doi: 10.3390/s23052607 (PMC10007171; doi:10.3390/s23052607)
Supplement: Supplementary file 1 [file sensors-23-02607-s001.zip › sensors-2200076-supplementary/Table S6 LCMS Raw Data.pdf]

Table S6: LCMS quantitation dataset (n = 734).

| | Concentration (mg/g) | | | | | | | | | | | | | |
|---|---|---|---|---|---|---|---|---|---|---|---|---|---|---|
| List ID | **CBDA** | **CBD** | **CBN** | **THC** | **CBC** | **THCA** | **CBDVA** | **CBDV** | **CBGA** | **CBG** | **THCV** | **THCVA** | **CBNA** | **CBCA** |
| 1 | 35.84 | 4.29 | 0.12 | 3.74 | 0.33 | 14.15 | 1.17 | 0.17 | 2.05 | 0.27 | 0.23 | 0.84 | 0.21 | 1.79 |
| 2 | 0.29 | 0.13 | 0.29 | 10.68 | 0.00 | 73.53 | 0.00 | 0.00 | 1.90 | 1.14 | 0.07 | 0.30 | 0.47 | 1.51 |
| 3 | 0.39 | 0.12 | 0.15 | 6.42 | 0.39 | 102.92 | 0.00 | 0.00 | 2.71 | 0.49 | 0.04 | 0.23 | 0.85 | 2.80 |
| 4 | 0.24 | 0.12 | 0.34 | 6.64 | 0.28 | 72.84 | 0.00 | 0.00 | 7.06 | 0.61 | 0.08 | 0.75 | 0.84 | 1.26 |
| 5 | 0.33 | 0.11 | 0.01 | 1.27 | 0.00 | 91.69 | 0.00 | 0.00 | 5.44 | 0.46 | 0.03 | 0.30 | 0.34 | 3.21 |
| 6 | 42.58 | 4.35 | 0.21 | 4.34 | 0.36 | 15.54 | 0.21 | 0.11 | 1.70 | 0.29 | 0.06 | 0.14 | 0.28 | 2.76 |
| 7 | 56.02 | 2.63 | 0.16 | 1.75 | 0.27 | 20.06 | 0.26 | 0.10 | 1.20 | 0.27 | 0.04 | 0.15 | 0.63 | 3.17 |
| 8 | 54.79 | 4.04 | 0.28 | 4.07 | 0.35 | 20.07 | 0.28 | 0.11 | 1.09 | 0.30 | 0.05 | 0.17 | 0.43 | 2.95 |
| 9 | 0.22 | 0.12 | 0.70 | 10.15 | 0.42 | 45.71 | 0.00 | 0.00 | 1.17 | 0.30 | 0.04 | 0.06 | 0.59 | 1.54 |
| 10 | 77.99 | 2.43 | 0.04 | 3.07 | 0.29 | 39.11 | 0.37 | 0.11 | 2.93 | 0.40 | 0.05 | 0.31 | 0.31 | 4.19 |
| 11 | 47.99 | 2.89 | 0.13 | 3.38 | 0.29 | 18.49 | 0.22 | 0.09 | 1.33 | 0.36 | 0.05 | 0.14 | 0.37 | 2.80 |
| 12 | 0.54 | 0.12 | 0.02 | 2.03 | 0.23 | 116.61 | 0.00 | 0.00 | 6.60 | 0.98 | 0.04 | 0.57 | 0.36 | 3.21 |
| 13 | 59.95 | 1.25 | 0.02 | 0.89 | 0.24 | 23.41 | 0.31 | 0.10 | 2.65 | 0.37 | 0.03 | 0.19 | 0.16 | 3.39 |
| 14 | 61.23 | 3.85 | 0.24 | 4.11 | 0.39 | 28.44 | 0.16 | 0.10 | 0.85 | 0.39 | 0.04 | 0.09 | 0.66 | 3.91 |
| 15 | 26.41 | 0.84 | 0.00 | 0.13 | 0.22 | 1.24 | 0.73 | 0.11 | 0.25 | 0.15 | 0.03 | 0.04 | 0.02 | 1.30 |
| 16 | 0.37 | 0.11 | 0.39 | 6.54 | 0.39 | 122.06 | 0.11 | 0.00 | 3.38 | 0.61 | 0.19 | 3.37 | 1.52 | 2.51 |
| 17 | 0.42 | 0.11 | 0.06 | 3.74 | 0.25 | 112.48 | 0.11 | 0.00 | 0.90 | 0.69 | 0.13 | 3.84 | 0.66 | 2.07 |
| 18 | 0.33 | 0.11 | 0.10 | 4.55 | 0.27 | 95.84 | 0.11 | 0.00 | 1.77 | 0.57 | 0.14 | 2.96 | 0.59 | 2.04 |
| 19 | 0.29 | 0.12 | 0.17 | 4.68 | 0.33 | 64.63 | 0.00 | 0.00 | 3.41 | 0.60 | 0.05 | 0.26 | 0.99 | 2.44 |
| 20 | 0.34 | 0.12 | 0.89 | 9.08 | 0.62 | 116.88 | 0.00 | 0.00 | 3.40 | 0.63 | 0.08 | 0.59 | 1.36 | 4.25 |
| 21 | 44.9 | 2.24 | 0.05 | 3.95 | 0.34 | 30.51 | 1.30 | 0.13 | 1.91 | 0.26 | 0.19 | 1.43 | 0.15 | 3.71 |
| 22 | 0.22 | 0.11 | 0.80 | 7.31 | 0.00 | 62.77 | 0.11 | 0.00 | 0.47 | 0.34 | 0.22 | 1.79 | 0.98 | 0.91 |

| | | | | | | | | | | | | | | |
|---|---|---|---|---|---|---|---|---|---|---|---|---|---|---|
| 23 | 0.31 | 0.12 | 0.66 | 9.01 | 0.63 | 108.46 | 0.11 | 0.00 | 2.03 | 0.50 | 0.31 | 3.09 | 1.41 | 3.43 |
| 24 | 32.77 | 2.76 | 0.05 | 2.46 | 0.28 | 16.61 | 1.05 | 0.13 | 1.57 | 0.25 | 0.12 | 0.89 | 0.09 | 2.11 |
| 25 | 0.21 | 0.11 | 0.59 | 7.20 | 0.35 | 50.36 | 0.00 | 0.00 | 1.75 | 0.32 | 0.04 | 0.09 | 0.73 | 2.02 |
| 26 | 29.32 | 3.12 | 0.13 | 2.36 | 0.28 | 10.27 | 1.35 | 0.16 | 0.59 | 0.16 | 0.17 | 0.84 | 0.25 | 2.03 |
| 27 | 0.39 | 0.11 | 0.03 | 3.23 | 0.28 | 121.87 | 0.00 | 0.00 | 3.57 | 0.64 | 0.03 | 0.27 | 0.62 | 3.54 |
| 28 | 60.46 | 2.32 | 0.13 | 3.01 | 0.27 | 34.02 | 0.24 | 0.11 | 0.83 | 0.32 | 0.04 | 0.22 | 0.72 | 3.41 |
| 29 | 104.14 | 3.47 | 0.04 | 4.27 | 0.39 | 48.73 | 0.21 | 0.10 | 2.09 | 0.43 | 0.04 | 0.17 | 0.36 | 5.95 |
| 30 | 44.01 | 6.06 | 0.26 | 5.63 | 0.58 | 12.04 | 0.13 | 0.10 | 2.00 | 0.39 | 0.04 | 0.04 | 0.22 | 2.97 |
| 31 | 0.3 | 0.11 | 0.37 | 7.32 | 0.33 | 102.47 | 0.00 | 0.00 | 3.06 | 0.31 | 0.07 | 0.62 | 1.27 | 2.02 |
| 32 | 59.79 | 5.65 | 0.48 | 7.65 | 0.53 | 34.61 | 0.15 | 0.11 | 0.77 | 0.49 | 0.05 | 0.10 | 0.59 | 3.64 |
| 33 | 65.37 | 5.63 | 0.29 | 5.96 | 0.47 | 28.20 | 0.16 | 0.11 | 0.67 | 0.38 | 0.04 | 0.09 | 0.44 | 3.75 |
| 34 | 0.29 | 0.12 | 0.38 | 4.51 | 0.41 | 80.66 | 0.00 | 0.00 | 2.97 | 0.46 | 0.04 | 0.31 | 1.47 | 3.82 |
| 35 | 0.32 | 0.12 | 0.43 | 9.73 | 0.35 | 85.05 | 0.00 | 0.00 | 2.94 | 0.58 | 0.07 | 0.39 | 0.83 | 2.22 |
| 36 | 0.29 | 0.13 | 0.62 | 10.90 | 0.37 | 99.59 | 0.00 | 0.00 | 4.33 | 0.77 | 0.07 | 0.31 | 0.75 | 1.91 |
| 37 | 0.44 | 0.11 | 0.23 | 5.86 | 0.27 | 122.39 | 0.00 | 0.00 | 2.07 | 0.82 | 0.05 | 0.53 | 1.52 | 1.85 |
| 38 | 0.36 | 0.12 | 0.50 | 9.20 | 0.33 | 91.01 | 0.00 | 0.00 | 2.91 | 1.10 | 0.05 | 0.26 | 1.03 | 1.82 |
| 39 | 78.49 | 4.86 | 0.24 | 6.23 | 0.46 | 42.23 | 0.33 | 0.11 | 2.35 | 1.01 | 0.06 | 0.32 | 0.46 | 4.46 |
| 40 | 64.51 | 5.37 | 0.25 | 5.27 | 0.42 | 25.30 | 0.33 | 0.11 | 1.20 | 0.41 | 0.07 | 0.22 | 0.40 | 3.41 |
| 41 | 70.01 | 4.46 | 0.26 | 5.25 | 0.39 | 36.71 | 0.29 | 0.11 | 1.83 | 0.52 | 0.06 | 0.28 | 0.56 | 4.06 |
| 42 | 0.27 | 0.12 | 0.13 | 4.33 | 0.24 | 79.78 | 0.00 | 0.00 | 2.50 | 0.75 | 0.05 | 0.62 | 0.78 | 1.21 |
| 43 | 35.64 | 4.51 | 0.26 | 4.00 | 0.37 | 16.55 | 1.04 | 0.17 | 1.58 | 0.30 | 0.20 | 0.84 | 0.30 | 2.13 |
| 44 | 75.09 | 4.84 | 0.19 | 5.23 | 0.46 | 30.89 | 0.17 | 0.10 | 0.93 | 0.56 | 0.04 | 0.11 | 0.40 | 4.35 |
| 45 | 0.34 | 0.13 | 0.48 | 8.38 | 0.36 | 104.35 | 0.00 | 0.00 | 4.47 | 0.42 | 0.05 | 0.34 | 0.98 | 2.58 |
| 46 | 53 | 0.99 | 0.02 | 0.76 | 0.23 | 25.52 | 0.23 | 0.10 | 2.15 | 0.44 | 0.03 | 0.18 | 0.30 | 2.89 |
| 47 | 0.41 | 0.12 | 0.16 | 6.53 | 0.28 | 138.30 | 0.00 | 0.00 | 4.88 | 0.93 | 0.07 | 0.89 | 1.21 | 2.19 |
| 48 | 0.34 | 0.13 | 0.59 | 12.60 | 0.46 | 99.32 | 0.00 | 0.00 | 2.55 | 0.48 | 0.09 | 0.45 | 0.76 | 2.84 |
| 49 | 45.82 | 2.92 | 0.16 | 2.65 | 0.31 | 17.65 | 0.25 | 0.11 | 1.06 | 0.23 | 0.05 | 0.17 | 0.40 | 3.46 |
| 50 | 0.29 | 0.12 | 0.33 | 7.36 | 0.43 | 104.90 | 0.00 | 0.00 | 2.81 | 0.63 | 0.08 | 0.71 | 1.01 | 2.03 |
| 51 | 0.35 | 0.11 | 0.08 | 3.45 | 0.00 | 131.31 | 0.00 | 0.00 | 4.41 | 1.02 | 0.05 | 1.11 | 1.13 | 1.93 |

| | | | | | | | | | | | | | | |
|---|---|---|---|---|---|---|---|---|---|---|---|---|---|---|
| 52 | 0.35 | 0.12 | 0.21 | 8.34 | 0.00 | 88.88 | 0.00 | 0.00 | 0.54 | 0.68 | 0.35 | 3.23 | 0.66 | 1.81 |
| 53 | 43.57 | 7.03 | 0.19 | 6.28 | 0.55 | 11.54 | 0.14 | 0.11 | 0.62 | 0.19 | 0.05 | 0.04 | 0.16 | 2.41 |
| 54 | 0.46 | 0.11 | 0.10 | 6.87 | 0.31 | 170.33 | 0.00 | 0.00 | 3.50 | 0.50 | 0.05 | 0.64 | 0.96 | 2.62 |
| 55 | 0.34 | 0.11 | 0.09 | 2.72 | 0.00 | 115.10 | 0.00 | 0.00 | 3.47 | 0.45 | 0.04 | 0.37 | 0.76 | 2.28 |
| 56 | 60.95 | 4.44 | 0.28 | 4.16 | 0.36 | 23.72 | 0.23 | 0.11 | 1.00 | 0.50 | 0.05 | 0.15 | 0.72 | 3.50 |
| 57 | 0.31 | 0.12 | 0.45 | 8.81 | 0.39 | 63.68 | 0.00 | 0.00 | 2.70 | 0.41 | 0.04 | 0.11 | 0.69 | 2.63 |
| 58 | 0.25 | 0.11 | 0.17 | 4.05 | 0.00 | 104.92 | 0.00 | 0.00 | 2.41 | 0.24 | 0.05 | 0.63 | 0.86 | 1.39 |
| 59 | 57.71 | 4.24 | 0.18 | 4.62 | 0.39 | 21.33 | 0.24 | 0.11 | 1.15 | 0.33 | 0.05 | 0.15 | 0.36 | 3.37 |
| 60 | 23.98 | 1.59 | 0.01 | 0.18 | 0.23 | 1.12 | 0.48 | 0.11 | 0.32 | 0.15 | 0.03 | 0.03 | 0.05 | 0.84 |
| 61 | 0.27 | 0.11 | 0.12 | 3.58 | 0.25 | 93.09 | 0.00 | 0.00 | 1.20 | 0.73 | 0.04 | 0.30 | 0.71 | 1.80 |
| 62 | 0.32 | 0.12 | 0.36 | 7.33 | 0.50 | 104.60 | 0.00 | 0.00 | 4.10 | 0.61 | 0.06 | 0.54 | 0.97 | 3.66 |
| 63 | 0.28 | 0.12 | 0.20 | 5.17 | 0.29 | 57.54 | 0.00 | 0.00 | 2.56 | 0.67 | 0.04 | 0.18 | 0.78 | 1.31 |
| 64 | 0.23 | 0.11 | 0.07 | 2.17 | 0.25 | 63.65 | 0.00 | 0.00 | 3.24 | 0.34 | 0.03 | 0.14 | 0.65 | 4.66 |
| 65 | 0.28 | 0.11 | 0.11 | 3.10 | 0.00 | 95.56 | 0.00 | 0.00 | 1.01 | 0.25 | 0.05 | 0.99 | 0.82 | 0.78 |
| 66 | 0.45 | 0.11 | 0.09 | 6.70 | 0.27 | 147.46 | 0.00 | 0.00 | 4.89 | 1.49 | 0.07 | 1.17 | 1.00 | 2.22 |
| 67 | 0.37 | 0.11 | 0.08 | 2.99 | 0.28 | 129.70 | 0.00 | 0.00 | 4.61 | 0.35 | 0.05 | 1.09 | 1.08 | 5.19 |
| 68 | 57.25 | 5.66 | 0.10 | 4.58 | 0.53 | 15.80 | 0.25 | 0.11 | 2.46 | 0.34 | 0.05 | 0.11 | 0.19 | 3.32 |
| 69 | 65.8 | 5.09 | 0.17 | 5.28 | 0.42 | 24.99 | 0.35 | 0.11 | 2.31 | 0.60 | 0.06 | 0.22 | 0.31 | 3.37 |
| 70 | 0.35 | 0.11 | 0.20 | 7.04 | 0.32 | 82.19 | 0.00 | 0.00 | 1.94 | 1.39 | 0.06 | 0.50 | 0.64 | 1.81 |
| 71 | 0.4 | 0.12 | 0.23 | 5.85 | 0.32 | 143.39 | 0.11 | 0.00 | 5.50 | 0.83 | 0.20 | 4.28 | 1.18 | 2.29 |
| 72 | 47.8 | 3.04 | 0.20 | 3.79 | 0.32 | 25.75 | 0.20 | 0.11 | 0.47 | 0.29 | 0.05 | 0.16 | 0.53 | 2.81 |
| 73 | 0.3 | 0.11 | 0.05 | 5.83 | 0.25 | 102.91 | 0.00 | 0.00 | 1.03 | 1.35 | 0.05 | 0.51 | 0.55 | 1.36 |
| 74 | 50.71 | 4.58 | 0.18 | 3.94 | 0.37 | 15.34 | 0.19 | 0.11 | 1.05 | 0.29 | 0.04 | 0.09 | 0.35 | 2.68 |
| 75 | 0.35 | 0.12 | 0.38 | 9.22 | 0.70 | 109.02 | 0.00 | 0.00 | 2.16 | 0.56 | 0.08 | 0.69 | 1.21 | 3.75 |
| 76 | 0.35 | 0.12 | 0.16 | 7.94 | 0.71 | 120.99 | 0.00 | 0.00 | 4.55 | 0.39 | 0.09 | 1.01 | 1.12 | 4.63 |
| 77 | 74.26 | 5.33 | 0.20 | 5.68 | 0.48 | 28.32 | 0.32 | 0.11 | 1.42 | 0.48 | 0.06 | 0.22 | 0.36 | 4.18 |
| 78 | 0.21 | 0.11 | 0.26 | 7.43 | 0.33 | 65.50 | 0.00 | 0.00 | 2.03 | 0.27 | 0.04 | 0.09 | 0.65 | 2.08 |
| 79 | 0.3 | 0.12 | 0.34 | 8.86 | 0.39 | 73.25 | 0.00 | 0.00 | 2.42 | 0.93 | 0.05 | 0.21 | 0.63 | 1.84 |
| 80 | 0.35 | 0.11 | 0.18 | 7.01 | 0.52 | 112.87 | 0.11 | 0.00 | 2.71 | 0.29 | 0.29 | 4.33 | 1.20 | 3.67 |

| | | | | | | | | | | | | | | |
|---|---|---|---|---|---|---|---|---|---|---|---|---|---|---|
| 81 | 0.24 | 0.11 | 0.10 | 4.02 | 0.24 | 73.56 | 0.00 | 0.00 | 1.96 | 0.70 | 0.05 | 0.61 | 0.86 | 1.25 |
| 82 | 0.29 | 0.11 | 0.17 | 4.69 | 0.00 | 89.78 | 0.00 | 0.00 | 3.87 | 0.54 | 0.04 | 0.35 | 1.41 | 1.09 |
| 83 | 0.3 | 0.11 | 0.11 | 6.05 | 0.28 | 101.69 | 0.00 | 0.00 | 3.81 | 0.72 | 0.05 | 0.49 | 0.92 | 1.89 |
| 84 | 0.3 | 0.11 | 0.38 | 9.43 | 0.00 | 83.00 | 0.00 | 0.00 | 0.69 | 0.80 | 0.28 | 2.32 | 0.79 | 1.38 |
| 85 | 61.42 | 3.06 | 0.10 | 3.22 | 0.32 | 24.33 | 0.27 | 0.11 | 1.91 | 0.39 | 0.05 | 0.17 | 0.39 | 3.65 |
| 86 | 27.44 | 3.29 | 0.11 | 3.22 | 0.33 | 12.62 | 0.85 | 0.15 | 1.23 | 0.23 | 0.18 | 0.67 | 0.20 | 1.72 |
| 87 | 0.4 | 0.12 | 0.39 | 9.55 | 0.66 | 123.99 | 0.00 | 0.00 | 2.54 | 0.43 | 0.08 | 0.54 | 1.19 | 3.36 |
| 88 | 0.34 | 0.12 | 0.14 | 4.67 | 0.33 | 86.26 | 0.11 | 0.00 | 1.71 | 0.37 | 0.16 | 2.74 | 0.71 | 4.63 |
| 89 | 0.27 | 0.11 | 0.12 | 4.09 | 0.38 | 83.37 | 0.00 | 0.00 | 2.04 | 0.25 | 0.04 | 0.28 | 0.96 | 2.84 |
| 90 | 37 | 2.67 | 0.10 | 2.89 | 0.28 | 16.12 | 0.21 | 0.11 | 1.19 | 0.19 | 0.05 | 0.16 | 0.28 | 2.17 |
| 91 | 0.34 | 0.12 | 0.25 | 6.66 | 0.35 | 112.97 | 0.00 | 0.00 | 4.19 | 0.30 | 0.06 | 0.70 | 1.33 | 1.98 |
| 92 | 0.29 | 0.12 | 0.41 | 10.49 | 0.51 | 76.69 | 0.00 | 0.00 | 2.13 | 0.74 | 0.30 | 1.87 | 0.76 | 2.48 |
| 93 | 66.55 | 5.37 | 0.11 | 4.99 | 0.48 | 22.46 | 0.15 | 0.10 | 0.89 | 0.20 | 0.04 | 0.07 | 0.22 | 3.91 |
| 94 | 46.07 | 2.50 | 0.17 | 1.87 | 0.26 | 16.66 | 0.19 | 0.10 | 0.65 | 0.19 | 0.04 | 0.11 | 0.60 | 2.42 |
| 95 | 46.52 | 3.57 | 0.18 | 3.55 | 0.35 | 18.23 | 0.24 | 0.11 | 1.41 | 0.26 | 0.05 | 0.18 | 0.30 | 3.23 |
| 96 | 0.33 | 0.12 | 0.12 | 4.83 | 0.24 | 98.22 | 0.00 | 0.00 | 3.08 | 0.43 | 0.07 | 1.07 | 0.98 | 1.60 |
| 97 | 0.32 | 0.11 | 0.05 | 2.30 | 0.23 | 91.88 | 0.00 | 0.00 | 3.55 | 0.37 | 0.03 | 0.13 | 0.49 | 2.86 |
| 98 | 0.33 | 0.12 | 0.33 | 8.99 | 0.30 | 79.09 | 0.00 | 0.00 | 3.63 | 0.69 | 0.06 | 0.30 | 0.77 | 1.56 |
| 99 | 68.09 | 2.95 | 0.13 | 3.22 | 0.29 | 29.69 | 0.34 | 0.11 | 2.17 | 0.34 | 0.05 | 0.25 | 0.64 | 3.12 |
| 100 | 0.25 | 0.12 | 0.60 | 14.03 | 0.52 | 48.09 | 0.00 | 0.00 | 1.84 | 0.39 | 0.05 | 0.07 | 0.49 | 2.00 |
| 101 | 0.27 | 0.12 | 0.32 | 8.67 | 0.58 | 62.97 | 0.00 | 0.00 | 2.32 | 0.57 | 0.05 | 0.13 | 0.69 | 2.53 |
| 102 | 65.39 | 3.53 | 0.10 | 3.73 | 0.33 | 24.44 | 0.15 | 0.10 | 1.17 | 0.30 | 0.04 | 0.07 | 0.38 | 3.68 |
| 103 | 38.71 | 4.86 | 0.31 | 6.12 | 0.60 | 22.72 | 1.00 | 0.17 | 1.00 | 0.26 | 0.30 | 1.01 | 0.37 | 3.48 |
| 104 | 0.33 | 0.12 | 0.46 | 10.75 | 0.44 | 104.28 | 0.11 | 0.00 | 2.41 | 0.42 | 0.43 | 3.27 | 0.90 | 2.38 |
| 105 | 45.89 | 7.17 | 0.16 | 5.16 | 0.51 | 12.07 | 0.22 | 0.11 | 1.74 | 0.30 | 0.06 | 0.09 | 0.22 | 2.36 |
| 106 | 0.37 | 0.11 | 0.05 | 4.88 | 0.29 | 112.73 | 0.00 | 0.00 | 5.77 | 2.09 | 0.05 | 0.84 | 0.64 | 2.07 |
| 107 | 0.32 | 0.12 | 0.19 | 10.31 | 0.30 | 91.91 | 0.00 | 0.00 | 2.23 | 1.38 | 0.10 | 0.69 | 0.54 | 1.48 |
| 108 | 0.29 | 0.12 | 0.27 | 7.14 | 0.53 | 53.92 | 0.00 | 0.00 | 1.75 | 0.77 | 0.06 | 0.22 | 0.79 | 2.70 |
| 109 | 0.28 | 0.11 | 0.08 | 2.85 | 0.23 | 67.14 | 0.00 | 0.00 | 1.34 | 0.56 | 0.08 | 1.70 | 0.62 | 0.96 |

| | | | | | | | | | | | | | | |
|---|---|---|---|---|---|---|---|---|---|---|---|---|---|---|
| 110 | 50.72 | 4.50 | 0.19 | 5.01 | 0.41 | 20.10 | 0.19 | 0.11 | 0.79 | 0.43 | 0.05 | 0.12 | 0.43 | 3.25 |
| 111 | 52.13 | 4.93 | 0.29 | 6.52 | 0.48 | 25.85 | 0.21 | 0.11 | 0.66 | 0.40 | 0.06 | 0.17 | 0.46 | 3.19 |
| 112 | 0.38 | 0.12 | 0.05 | 4.18 | 0.23 | 103.12 | 0.00 | 0.00 | 3.25 | 0.65 | 0.05 | 0.70 | 0.70 | 1.53 |
| 113 | 29.78 | 2.89 | 0.13 | 2.50 | 0.27 | 13.18 | 1.01 | 0.14 | 0.91 | 0.19 | 0.15 | 0.82 | 0.29 | 1.46 |
| 114 | 0.61 | 0.12 | 0.17 | 6.26 | 0.31 | 84.76 | 0.00 | 0.00 | 3.05 | 0.46 | 0.06 | 0.42 | 1.01 | 1.85 |
| 115 | 0.31 | 0.12 | 0.35 | 9.62 | 0.00 | 63.85 | 0.00 | 0.00 | 2.80 | 0.58 | 0.06 | 0.23 | 0.72 | 1.14 |
| 116 | 71.69 | 5.86 | 0.24 | 6.83 | 0.51 | 31.17 | 0.16 | 0.10 | 0.66 | 0.44 | 0.05 | 0.10 | 0.44 | 3.85 |
| 117 | 0.27 | 0.12 | 0.27 | 6.87 | 0.90 | 70.22 | 0.00 | 0.00 | 0.51 | 0.53 | 0.23 | 2.16 | 1.21 | 3.35 |
| 118 | 80.9 | 3.57 | 0.15 | 4.76 | 0.36 | 46.04 | 0.33 | 0.10 | 2.24 | 0.89 | 0.05 | 0.34 | 0.61 | 4.30 |
| 119 | 0.39 | 0.12 | 0.28 | 8.40 | 0.34 | 94.79 | 0.00 | 0.00 | 4.88 | 0.50 | 0.07 | 0.48 | 0.95 | 2.21 |
| 120 | 59.27 | 2.74 | 0.10 | 2.78 | 0.30 | 24.71 | 0.26 | 0.10 | 1.69 | 0.36 | 0.04 | 0.17 | 0.53 | 3.44 |
| 121 | 85.89 | 5.87 | 0.21 | 5.81 | 0.51 | 36.70 | 0.39 | 0.11 | 1.90 | 0.45 | 0.07 | 0.29 | 0.49 | 4.41 |
| 122 | 0.28 | 0.11 | 0.15 | 4.15 | 0.00 | 97.62 | 0.00 | 0.00 | 2.28 | 0.30 | 0.06 | 1.03 | 1.14 | 0.80 |
| 123 | 60.11 | 4.76 | 0.17 | 4.79 | 0.41 | 22.52 | 0.25 | 0.11 | 1.16 | 0.32 | 0.05 | 0.16 | 0.37 | 3.30 |
| 124 | 65.98 | 3.82 | 0.07 | 3.36 | 0.34 | 21.15 | 0.27 | 0.11 | 1.53 | 0.26 | 0.05 | 0.14 | 0.26 | 3.39 |
| 125 | 70.02 | 5.77 | 0.27 | 6.14 | 0.51 | 28.43 | 0.31 | 0.11 | 1.20 | 0.49 | 0.06 | 0.22 | 0.50 | 3.88 |
| 126 | 64.14 | 5.06 | 0.32 | 3.87 | 0.37 | 21.03 | 0.14 | 0.10 | 0.35 | 0.28 | 0.03 | 0.05 | 0.80 | 3.58 |
| 127 | 0.37 | 0.12 | 0.12 | 5.29 | 0.25 | 107.38 | 0.00 | 0.00 | 4.49 | 0.28 | 0.04 | 0.32 | 0.86 | 1.97 |
| 128 | 59.34 | 5.03 | 0.35 | 3.79 | 0.35 | 21.06 | 0.32 | 0.11 | 1.04 | 0.32 | 0.05 | 0.21 | 0.75 | 2.86 |
| 129 | 0.29 | 0.12 | 0.23 | 5.83 | 0.00 | 86.58 | 0.00 | 0.00 | 0.64 | 0.56 | 0.15 | 2.11 | 0.91 | 1.28 |
| 130 | 0.26 | 0.11 | 0.11 | 2.97 | 0.22 | 71.79 | 0.00 | 0.00 | 5.17 | 0.35 | 0.05 | 0.71 | 0.79 | 1.05 |
| 131 | 39.66 | 0.72 | 0.02 | 0.49 | 0.22 | 12.87 | 0.19 | 0.00 | 2.09 | 0.17 | 0.03 | 0.09 | 0.25 | 2.15 |
| 132 | 0.46 | 0.12 | 0.29 | 6.67 | 0.34 | 134.40 | 0.11 | 0.00 | 1.15 | 0.34 | 0.25 | 3.90 | 1.13 | 3.32 |
| 133 | 34.2 | 2.28 | 0.01 | 0.23 | 0.24 | 1.27 | 0.58 | 0.12 | 0.33 | 0.00 | 0.03 | 0.03 | 0.08 | 1.20 |
| 134 | 0.35 | 0.11 | 0.09 | 4.58 | 0.28 | 115.13 | 0.11 | 0.00 | 3.00 | 0.42 | 0.11 | 2.36 | 0.80 | 3.16 |
| 135 | 51.88 | 2.19 | 0.11 | 3.18 | 0.30 | 34.44 | 0.13 | 0.00 | 0.60 | 0.37 | 0.03 | 0.10 | 0.53 | 3.22 |
| 136 | 63.67 | 1.24 | 0.05 | 1.31 | 0.00 | 30.13 | 0.29 | 0.10 | 0.89 | 0.30 | 0.04 | 0.24 | 0.53 | 3.37 |
| 137 | 0.3 | 0.00 | 0.06 | 2.38 | 0.25 | 61.65 | 0.00 | 0.00 | 2.71 | 0.49 | 0.03 | 0.12 | 0.49 | 3.63 |
| 138 | 54.62 | 1.49 | 0.05 | 1.29 | 0.23 | 21.56 | 0.25 | 0.10 | 1.16 | 0.23 | 0.03 | 0.16 | 0.42 | 2.76 |

| | | | | | | | | | | | | | | |
|---|---|---|---|---|---|---|---|---|---|---|---|---|---|---|
| 139 | 41.41 | 12.31 | 0.64 | 8.77 | 0.75 | 10.48 | 0.21 | 0.13 | 0.97 | 0.23 | 0.09 | 0.10 | 0.22 | 1.89 |
| 140 | 0.31 | 0.00 | 0.10 | 2.58 | 0.25 | 114.98 | 0.00 | 0.00 | 0.70 | 0.54 | 0.11 | 3.48 | 1.09 | 2.99 |
| 141 | 0.31 | 0.11 | 0.10 | 3.78 | 0.24 | 90.24 | 0.00 | 0.00 | 2.00 | 0.31 | 0.04 | 0.38 | 0.71 | 2.28 |
| 142 | 0.32 | 0.12 | 0.21 | 5.00 | 0.27 | 127.80 | 0.00 | 0.00 | 2.03 | 0.37 | 0.05 | 0.55 | 1.25 | 2.58 |
| 143 | 0.33 | 0.11 | 0.12 | 4.04 | 0.23 | 93.17 | 0.11 | 0.00 | 1.30 | 0.33 | 0.13 | 2.90 | 0.93 | 1.24 |
| 144 | 0.27 | 0.11 | 0.12 | 3.24 | 0.24 | 69.41 | 0.00 | 0.00 | 1.34 | 0.55 | 0.08 | 1.46 | 0.87 | 1.73 |
| 145 | 76 | 1.69 | 0.05 | 1.72 | 0.25 | 32.81 | 0.27 | 0.10 | 1.17 | 0.52 | 0.04 | 0.20 | 0.43 | 3.97 |
| 146 | 0.3 | 0.12 | 0.14 | 8.24 | 0.23 | 85.97 | 0.00 | 0.00 | 1.04 | 0.34 | 0.11 | 1.01 | 0.45 | 0.99 |
| 147 | 89.95 | 2.41 | 0.03 | 2.83 | 0.31 | 43.00 | 0.18 | 0.00 | 2.35 | 0.55 | 0.03 | 0.15 | 0.34 | 5.04 |
| 148 | 0.24 | 0.11 | 0.13 | 3.26 | 0.00 | 78.91 | 0.00 | 0.00 | 0.91 | 0.70 | 0.04 | 0.30 | 0.74 | 1.11 |
| 149 | 60.99 | 2.21 | 0.06 | 1.85 | 0.25 | 24.08 | 0.32 | 0.10 | 1.79 | 0.36 | 0.04 | 0.23 | 0.35 | 2.90 |
| 150 | 43.8 | 2.39 | 0.08 | 1.88 | 0.25 | 13.17 | 0.87 | 0.12 | 1.06 | 0.20 | 0.07 | 0.40 | 0.34 | 1.83 |
| 151 | 0.52 | 0.13 | 0.24 | 7.65 | 0.27 | 73.77 | 0.00 | 0.00 | 1.56 | 0.43 | 0.06 | 0.39 | 0.82 | 1.34 |
| 152 | 40.17 | 0.76 | 0.04 | 0.81 | 0.22 | 19.82 | 0.18 | 0.00 | 0.37 | 0.20 | 0.03 | 0.12 | 0.39 | 2.70 |
| 153 | 0.65 | 0.12 | 0.08 | 4.89 | 0.30 | 117.26 | 0.00 | 0.00 | 8.72 | 0.48 | 0.05 | 0.77 | 0.87 | 2.65 |
| 154 | 0.27 | 0.11 | 0.28 | 4.89 | 0.27 | 89.13 | 0.00 | 0.00 | 3.17 | 0.30 | 0.05 | 0.57 | 1.31 | 1.43 |
| 155 | 49 | 1.35 | 0.04 | 1.01 | 0.23 | 18.63 | 0.20 | 0.10 | 1.03 | 0.24 | 0.03 | 0.11 | 0.30 | 2.61 |
| 156 | 0.33 | 0.12 | 0.02 | 4.20 | 0.35 | 123.60 | 0.00 | 0.00 | 4.06 | 1.91 | 0.04 | 0.58 | 0.53 | 4.16 |
| 157 | 0.38 | 0.12 | 0.03 | 4.85 | 0.27 | 128.09 | 0.00 | 0.00 | 6.14 | 0.94 | 0.05 | 0.68 | 0.64 | 2.57 |
| 158 | 0.34 | 0.12 | 0.42 | 7.31 | 0.00 | 91.25 | 0.00 | 0.00 | 1.26 | 1.08 | 0.05 | 0.42 | 1.10 | 1.46 |
| 159 | 84.82 | 1.28 | 0.03 | 1.12 | 0.23 | 43.39 | 0.37 | 0.10 | 2.57 | 0.42 | 0.03 | 0.33 | 0.48 | 4.72 |
| 160 | 71.94 | 4.08 | 0.16 | 4.31 | 0.38 | 30.25 | 0.29 | 0.11 | 1.60 | 0.46 | 0.05 | 0.22 | 0.39 | 4.22 |
| 161 | 0.36 | 0.11 | 0.08 | 2.57 | 0.00 | 100.77 | 0.00 | 0.00 | 1.42 | 0.33 | 0.05 | 1.08 | 0.80 | 0.87 |
| 162 | 59.16 | 5.84 | 0.36 | 5.98 | 0.44 | 22.74 | 0.25 | 0.11 | 0.50 | 0.21 | 0.06 | 0.17 | 0.42 | 3.17 |
| 163 | 40.84 | 1.39 | 0.02 | 1.59 | 0.24 | 20.47 | 1.31 | 0.12 | 2.92 | 0.27 | 0.09 | 1.16 | 0.18 | 2.08 |
| 164 | 0.43 | 0.12 | 0.09 | 3.27 | 0.28 | 97.82 | 0.00 | 0.00 | 3.99 | 0.41 | 0.05 | 0.67 | 0.83 | 2.62 |
| 165 | 0.31 | 0.12 | 0.16 | 5.27 | 0.26 | 86.34 | 0.00 | 0.00 | 2.26 | 1.02 | 0.06 | 0.71 | 0.84 | 1.34 |
| 166 | 0.42 | 0.12 | 0.12 | 3.82 | 0.00 | 121.88 | 0.00 | 0.00 | 2.64 | 0.50 | 0.05 | 0.59 | 0.86 | 1.97 |
| 167 | 53.64 | 0.96 | 0.05 | 1.10 | 0.22 | 31.53 | 0.26 | 0.10 | 1.03 | 0.30 | 0.03 | 0.24 | 0.56 | 2.65 |

OFFICIAL

| | | | | | | | | | | | | | | |
|---|---|---|---|---|---|---|---|---|---|---|---|---|---|---|
| 168 | 0.38 | 0.11 | 0.18 | 6.39 | 0.28 | 99.83 | 0.00 | 0.00 | 2.06 | 0.73 | 0.05 | 0.45 | 0.88 | 1.54 |
| 169 | 57.55 | 1.86 | 0.05 | 1.34 | 0.24 | 19.15 | 0.27 | 0.10 | 2.08 | 0.23 | 0.04 | 0.15 | 0.31 | 2.83 |
| 170 | 0.35 | 0.12 | 0.46 | 7.72 | 0.27 | 81.32 | 0.00 | 0.00 | 4.11 | 0.45 | 0.08 | 0.60 | 1.12 | 1.39 |
| 171 | 65.97 | 4.10 | 0.18 | 3.71 | 0.37 | 23.90 | 0.15 | 0.10 | 0.54 | 0.22 | 0.04 | 0.07 | 0.39 | 4.14 |
| 172 | 0.36 | 0.12 | 0.39 | 7.65 | 0.30 | 72.24 | 0.00 | 0.00 | 1.17 | 0.40 | 0.04 | 0.11 | 0.99 | 1.95 |
| 173 | 35.19 | 1.30 | 0.03 | 1.37 | 0.24 | 14.96 | 1.59 | 0.12 | 1.54 | 0.19 | 0.11 | 1.18 | 0.18 | 2.23 |
| 174 | 0.32 | 0.12 | 0.38 | 7.06 | 0.32 | 54.60 | 0.00 | 0.00 | 1.42 | 0.46 | 0.15 | 1.21 | 0.88 | 1.52 |
| 175 | 71.18 | 1.74 | 0.03 | 1.76 | 0.25 | 31.67 | 0.32 | 0.10 | 1.56 | 0.39 | 0.04 | 0.26 | 0.28 | 3.32 |
| 176 | 56.71 | 4.98 | 0.29 | 4.38 | 0.37 | 20.59 | 0.30 | 0.11 | 1.44 | 0.46 | 0.06 | 0.19 | 0.50 | 2.73 |
| 177 | 0.31 | 0.12 | 0.11 | 7.28 | 0.43 | 67.90 | 0.00 | 0.00 | 2.71 | 0.88 | 0.04 | 0.11 | 0.59 | 2.34 |
| 178 | 0.35 | 0.11 | 0.09 | 4.64 | 0.24 | 74.23 | 0.11 | 0.00 | 0.38 | 0.27 | 0.09 | 1.18 | 0.59 | 1.12 |
| 179 | 0.34 | 0.12 | 0.57 | 13.15 | 0.67 | 99.53 | 0.11 | 0.00 | 2.33 | 0.39 | 0.49 | 2.94 | 1.04 | 2.33 |
| 180 | 71.47 | 2.38 | 0.05 | 3.56 | 0.29 | 46.99 | 0.16 | 0.10 | 1.13 | 0.62 | 0.04 | 0.15 | 0.40 | 3.90 |
| 181 | 43.44 | 2.63 | 0.14 | 2.75 | 0.28 | 20.50 | 0.20 | 0.10 | 0.79 | 0.27 | 0.04 | 0.13 | 0.40 | 2.65 |
| 182 | 0.53 | 0.12 | 0.03 | 2.98 | 0.26 | 98.10 | 0.00 | 0.00 | 3.16 | 0.53 | 0.03 | 0.21 | 0.57 | 2.98 |
| 183 | 69.33 | 6.87 | 0.16 | 7.01 | 0.56 | 24.04 | 0.38 | 0.11 | 3.88 | 0.78 | 0.08 | 0.24 | 0.33 | 3.32 |
| 184 | 0.35 | 0.12 | 0.41 | 7.04 | 0.32 | 107.55 | 0.00 | 0.00 | 1.76 | 0.35 | 0.05 | 0.41 | 1.40 | 1.76 |
| 185 | 0.3 | 0.12 | 0.32 | 5.16 | 0.28 | 75.59 | 0.00 | 0.00 | 4.09 | 0.42 | 0.06 | 0.58 | 1.24 | 1.51 |
| 186 | 62.44 | 3.74 | 0.12 | 3.38 | 0.32 | 22.52 | 0.30 | 0.11 | 1.88 | 0.34 | 0.05 | 0.20 | 0.33 | 3.07 |
| 187 | 44.41 | 3.17 | 0.15 | 2.82 | 0.29 | 14.37 | 0.20 | 0.10 | 0.58 | 0.20 | 0.04 | 0.10 | 0.38 | 2.27 |
| 188 | 0.54 | 0.14 | 0.21 | 8.34 | 0.44 | 93.88 | 0.00 | 0.00 | 6.21 | 0.78 | 0.06 | 0.34 | 0.61 | 2.97 |
| 189 | 17.48 | 2.03 | 0.10 | 1.60 | 0.27 | 6.08 | 1.16 | 0.16 | 0.32 | 0.15 | 0.16 | 0.64 | 0.17 | 1.08 |
| 190 | 0.29 | 0.12 | 0.39 | 6.63 | 0.00 | 83.82 | 0.00 | 0.00 | 3.01 | 0.29 | 0.05 | 0.26 | 1.16 | 1.72 |
| 191 | 41.71 | 0.78 | 0.02 | 0.53 | 0.22 | 15.02 | 0.17 | 0.00 | 1.11 | 0.24 | 0.03 | 0.09 | 0.21 | 2.34 |
| 192 | 0.32 | 0.12 | 0.44 | 6.39 | 0.54 | 97.04 | 0.00 | 0.00 | 1.26 | 0.47 | 0.06 | 0.51 | 1.62 | 4.47 |
| 193 | 80.48 | 2.59 | 0.03 | 3.56 | 0.34 | 44.23 | 0.17 | 0.00 | 1.96 | 0.57 | 0.04 | 0.14 | 0.26 | 4.65 |
| 194 | 0.34 | 0.13 | 0.86 | 9.58 | 0.40 | 110.83 | 0.00 | 0.00 | 3.87 | 0.59 | 0.06 | 0.39 | 1.58 | 2.60 |
| 195 | 0.28 | 0.12 | 0.16 | 7.55 | 0.43 | 64.25 | 0.00 | 0.00 | 2.73 | 0.41 | 0.04 | 0.12 | 0.56 | 2.13 |
| 196 | 0.28 | 0.11 | 0.05 | 2.02 | 0.24 | 57.66 | 0.00 | 0.00 | 3.18 | 0.40 | 0.03 | 0.10 | 0.51 | 2.22 |

OFFICIAL

| | | | | | | | | | | | | | | |
|---|---|---|---|---|---|---|---|---|---|---|---|---|---|---|
| 197 | 0.32 | 0.00 | 0.16 | 5.19 | 0.26 | 89.08 | 0.00 | 0.00 | 3.70 | 0.64 | 0.05 | 0.48 | 0.93 | 1.66 |
| 198 | 0.26 | 0.11 | 0.11 | 3.55 | 0.23 | 84.07 | 0.00 | 0.00 | 0.43 | 0.36 | 0.09 | 2.06 | 0.71 | 1.20 |
| 199 | 0.28 | 0.11 | 0.32 | 3.56 | 0.27 | 95.81 | 0.00 | 0.00 | 2.59 | 0.24 | 0.04 | 0.62 | 2.47 | 1.52 |
| 200 | 70.83 | 1.86 | 0.05 | 1.94 | 0.26 | 32.37 | 0.25 | 0.10 | 1.50 | 0.60 | 0.04 | 0.21 | 0.42 | 3.91 |
| 201 | 47.45 | 4.78 | 0.24 | 4.55 | 0.44 | 16.11 | 0.13 | 0.10 | 1.01 | 0.26 | 0.04 | 0.06 | 0.31 | 3.19 |
| 202 | 0.32 | 0.00 | 0.10 | 2.54 | 0.22 | 83.56 | 0.00 | 0.00 | 0.55 | 0.70 | 0.04 | 0.58 | 0.65 | 1.20 |
| 203 | 67.2 | 3.98 | 0.17 | 3.28 | 0.34 | 26.03 | 0.35 | 0.11 | 1.65 | 0.49 | 0.05 | 0.24 | 0.52 | 3.50 |
| 204 | 0.42 | 0.14 | 0.39 | 12.12 | 0.00 | 79.72 | 0.00 | 0.00 | 2.87 | 0.72 | 0.07 | 0.31 | 0.78 | 1.03 |
| 205 | 0.27 | 0.11 | 0.10 | 4.59 | 0.31 | 87.46 | 0.00 | 0.00 | 1.36 | 0.43 | 0.13 | 2.49 | 0.84 | 1.83 |
| 206 | 0.39 | 0.12 | 0.34 | 11.96 | 0.38 | 125.32 | 0.00 | 0.00 | 1.98 | 0.88 | 0.09 | 0.63 | 0.92 | 1.84 |
| 207 | 28.18 | 1.89 | 0.04 | 1.50 | 0.25 | 12.70 | 0.17 | 0.10 | 0.77 | 0.18 | 0.04 | 0.11 | 0.15 | 1.57 |
| 208 | 9.83 | 3.24 | 0.01 | 0.23 | 0.26 | 0.66 | 0.19 | 0.12 | 0.17 | 0.14 | 0.03 | 0.01 | 0.02 | 0.34 |
| 209 | 0.28 | 0.00 | 0.10 | 4.05 | 0.24 | 100.57 | 0.00 | 0.00 | 3.12 | 0.98 | 0.04 | 0.33 | 0.77 | 1.77 |
| 210 | 0.27 | 0.13 | 0.33 | 11.41 | 0.47 | 76.07 | 0.00 | 0.00 | 3.01 | 0.59 | 0.05 | 0.15 | 0.60 | 2.46 |
| 211 | 22.53 | 1.06 | 0.03 | 0.89 | 0.23 | 9.97 | 1.33 | 0.12 | 0.57 | 0.16 | 0.09 | 1.00 | 0.16 | 1.32 |
| 212 | 0.34 | 0.11 | 0.10 | 4.02 | 0.24 | 118.30 | 0.00 | 0.00 | 1.47 | 0.72 | 0.05 | 0.61 | 0.85 | 1.56 |
| 213 | 0.33 | 0.12 | 0.13 | 5.15 | 0.26 | 110.49 | 0.00 | 0.00 | 7.64 | 0.49 | 0.05 | 0.71 | 0.99 | 2.12 |
| 214 | 0.33 | 0.11 | 0.10 | 3.04 | 0.28 | 69.98 | 0.00 | 0.00 | 2.49 | 0.55 | 0.03 | 0.13 | 0.67 | 2.98 |
| 215 | 0.36 | 0.12 | 0.43 | 14.17 | 0.58 | 88.46 | 0.00 | 0.00 | 4.74 | 2.66 | 0.14 | 0.63 | 0.64 | 1.97 |
| 216 | 78.59 | 2.73 | 0.10 | 3.13 | 0.29 | 35.65 | 0.32 | 0.11 | 1.77 | 0.31 | 0.04 | 0.24 | 0.51 | 4.06 |
| 217 | 0.32 | 0.00 | 0.19 | 6.83 | 0.31 | 97.63 | 0.00 | 0.00 | 4.81 | 1.04 | 0.05 | 0.33 | 0.78 | 1.71 |
| 218 | 0.31 | 0.12 | 0.26 | 7.27 | 0.73 | 101.53 | 0.00 | 0.00 | 2.59 | 0.45 | 0.05 | 0.29 | 1.23 | 3.85 |
| 219 | 70.33 | 2.74 | 0.13 | 3.76 | 0.31 | 47.10 | 0.16 | 0.10 | 0.81 | 0.57 | 0.04 | 0.15 | 0.59 | 3.76 |
| 220 | 0.34 | 0.12 | 0.21 | 4.82 | 0.25 | 93.50 | 0.00 | 0.00 | 4.57 | 0.57 | 0.05 | 0.48 | 1.16 | 1.60 |
| 221 | 0.34 | 0.00 | 0.17 | 4.03 | 0.24 | 79.96 | 0.00 | 0.00 | 0.69 | 0.64 | 0.05 | 0.52 | 0.69 | 1.43 |
| 222 | 37.5 | 2.64 | 0.18 | 2.78 | 0.28 | 17.34 | 0.17 | 0.10 | 0.57 | 0.25 | 0.04 | 0.12 | 0.43 | 2.14 |
| 223 | 38.37 | 4.39 | 0.13 | 3.69 | 0.39 | 13.27 | 1.86 | 0.22 | 1.00 | 0.21 | 0.30 | 1.13 | 0.23 | 2.33 |
| 224 | 51.67 | 3.43 | 0.27 | 3.97 | 0.35 | 24.59 | 0.23 | 0.11 | 0.54 | 0.28 | 0.05 | 0.17 | 0.61 | 3.14 |
| 225 | 72.4 | 2.58 | 0.04 | 2.59 | 0.30 | 28.88 | 0.16 | 0.00 | 1.39 | 0.20 | 0.04 | 0.10 | 0.24 | 4.02 |

| | | | | | | | | | | | | | | |
|---|---|---|---|---|---|---|---|---|---|---|---|---|---|---|
| 226 | 60.98 | 1.15 | 0.04 | 1.13 | 0.23 | 31.24 | 0.26 | 0.00 | 2.18 | 0.33 | 0.03 | 0.22 | 0.48 | 3.36 |
| 227 | 0.37 | 0.12 | 0.29 | 5.91 | 0.31 | 110.68 | 0.00 | 0.00 | 3.92 | 0.95 | 0.05 | 0.58 | 1.53 | 2.15 |
| 228 | 0.4 | 0.00 | 0.04 | 4.32 | 0.28 | 102.64 | 0.00 | 0.00 | 4.33 | 2.08 | 0.05 | 0.69 | 0.53 | 1.93 |
| 229 | 0.24 | 0.00 | 0.33 | 10.37 | 0.54 | 67.45 | 0.00 | 0.00 | 3.89 | 1.41 | 0.08 | 0.30 | 0.70 | 2.31 |
| 230 | 60.52 | 5.49 | 0.13 | 7.21 | 0.55 | 25.57 | 0.26 | 0.11 | 0.79 | 0.32 | 0.07 | 0.18 | 0.27 | 3.37 |
| 231 | 0.29 | 0.12 | 0.25 | 12.47 | 0.33 | 83.54 | 0.00 | 0.00 | 6.36 | 0.72 | 0.20 | 1.06 | 0.57 | 1.33 |
| 232 | 0.43 | 0.11 | 0.09 | 6.55 | 0.35 | 97.66 | 0.00 | 0.00 | 1.79 | 0.42 | 0.06 | 0.61 | 0.68 | 2.74 |
| 233 | 0.3 | 0.12 | 0.33 | 11.55 | 0.38 | 96.96 | 0.00 | 0.00 | 4.43 | 0.83 | 0.09 | 0.52 | 0.78 | 1.93 |
| 234 | 60.06 | 4.32 | 0.08 | 6.36 | 0.42 | 31.10 | 0.26 | 0.11 | 1.66 | 0.38 | 0.06 | 0.24 | 0.26 | 3.16 |
| 235 | 0.29 | 0.12 | 0.51 | 8.20 | 0.47 | 73.89 | 0.00 | 0.00 | 0.61 | 0.50 | 0.30 | 2.50 | 1.15 | 3.13 |
| 236 | 0.31 | 0.00 | 0.07 | 5.32 | 0.25 | 86.79 | 0.00 | 0.00 | 0.40 | 0.33 | 0.10 | 1.37 | 0.54 | 1.36 |
| 237 | 0.31 | 0.11 | 0.04 | 2.75 | 0.22 | 102.85 | 0.00 | 0.00 | 4.27 | 0.61 | 0.04 | 0.52 | 0.69 | 1.21 |
| 238 | 54.22 | 3.47 | 0.12 | 3.54 | 0.32 | 20.74 | 0.25 | 0.11 | 2.82 | 0.53 | 0.05 | 0.17 | 0.34 | 2.67 |
| 239 | 0.26 | 0.12 | 0.34 | 7.21 | 0.34 | 80.77 | 0.00 | 0.00 | 3.82 | 0.34 | 0.05 | 0.26 | 1.00 | 2.13 |
| 240 | 69.96 | 1.54 | 0.02 | 1.88 | 0.25 | 34.72 | 0.15 | 0.00 | 1.32 | 0.36 | 0.04 | 0.09 | 0.21 | 3.15 |
| 241 | 63.63 | 3.71 | 0.18 | 3.19 | 0.30 | 26.91 | 0.26 | 0.10 | 1.04 | 0.25 | 0.06 | 0.17 | 0.43 | 2.34 |
| 242 | 0.29 | 0.13 | 0.14 | 3.32 | 0.27 | 97.69 | 0.00 | 0.00 | 2.05 | 0.96 | 0.04 | 0.29 | 1.06 | 2.04 |
| 243 | 54.94 | 2.58 | 0.18 | 2.45 | 0.24 | 22.95 | 0.21 | 0.10 | 0.66 | 0.22 | 0.05 | 0.12 | 0.55 | 1.91 |
| 244 | 0.31 | 0.00 | 0.06 | 4.07 | 0.25 | 109.12 | 0.00 | 0.00 | 2.93 | 1.37 | 0.05 | 0.50 | 0.52 | 1.52 |
| 245 | 0.23 | 0.11 | 0.33 | 5.31 | 0.22 | 70.69 | 0.00 | 0.00 | 0.54 | 0.28 | 0.04 | 0.06 | 0.85 | 1.28 |
| 246 | 0.23 | 0.11 | 0.12 | 4.69 | 0.26 | 77.18 | 0.00 | 0.00 | 1.95 | 0.29 | 0.04 | 0.10 | 0.60 | 1.60 |
| 247 | 0.25 | 0.12 | 0.49 | 5.93 | 0.34 | 104.82 | 0.00 | 0.00 | 2.44 | 0.26 | 0.16 | 1.69 | 1.32 | 1.88 |
| 248 | 0.21 | 0.11 | 0.04 | 2.27 | 0.20 | 73.63 | 0.00 | 0.00 | 1.69 | 0.30 | 0.05 | 0.61 | 0.56 | 1.04 |
| 249 | 0.23 | 0.11 | 0.16 | 6.32 | 0.28 | 79.30 | 0.00 | 0.00 | 1.96 | 0.32 | 0.04 | 0.10 | 0.64 | 1.97 |
| 250 | 0.35 | 0.00 | 0.08 | 5.36 | 0.25 | 107.77 | 0.00 | 0.00 | 4.38 | 0.71 | 0.05 | 0.40 | 0.72 | 2.12 |
| 251 | 0.27 | 0.13 | 0.36 | 10.54 | 0.70 | 59.86 | 0.00 | 0.00 | 3.35 | 0.69 | 0.06 | 0.14 | 0.66 | 2.58 |
| 252 | 0.24 | 0.12 | 0.53 | 7.27 | 0.23 | 66.15 | 0.00 | 0.00 | 4.80 | 0.49 | 0.09 | 0.62 | 1.08 | 1.29 |
| 253 | 0.31 | 0.13 | 0.41 | 8.50 | 0.30 | 91.29 | 0.11 | 0.00 | 2.80 | 0.74 | 0.46 | 4.06 | 1.01 | 1.63 |
| 254 | 29.97 | 1.31 | 0.04 | 1.42 | 0.21 | 13.86 | 0.16 | 0.09 | 0.94 | 0.16 | 0.04 | 0.10 | 0.23 | 1.70 |

| | | | | | | | | | | | | | | |
|---|---|---|---|---|---|---|---|---|---|---|---|---|---|---|
| 255 | 29.42 | 2.95 | 0.12 | 3.27 | 0.26 | 14.74 | 0.81 | 0.13 | 1.54 | 0.23 | 0.17 | 0.73 | 0.30 | 1.68 |
| 256 | 74.36 | 2.77 | 0.06 | 2.69 | 0.28 | 25.60 | 0.30 | 0.10 | 2.96 | 0.24 | 0.04 | 0.17 | 0.36 | 4.10 |
| 257 | 0.32 | 0.13 | 0.25 | 9.36 | 0.50 | 75.36 | 0.00 | 0.00 | 2.95 | 0.73 | 0.08 | 0.30 | 0.67 | 2.78 |
| 258 | 66.39 | 4.88 | 0.19 | 4.93 | 0.42 | 22.77 | 0.33 | 0.10 | 1.96 | 0.38 | 0.06 | 0.20 | 0.40 | 3.59 |
| 259 | 0.38 | 0.00 | 0.14 | 6.74 | 0.29 | 122.45 | 0.00 | 0.00 | 4.10 | 0.68 | 0.07 | 0.77 | 1.06 | 2.77 |
| 260 | 68.68 | 6.03 | 0.28 | 7.00 | 0.52 | 29.08 | 0.32 | 0.11 | 1.49 | 0.45 | 0.08 | 0.26 | 0.52 | 3.73 |
| 261 | 51.23 | 5.74 | 0.18 | 5.93 | 0.57 | 16.10 | 0.15 | 0.10 | 1.05 | 0.22 | 0.05 | 0.06 | 0.25 | 3.25 |
| 262 | 0.53 | 0.12 | 0.12 | 3.41 | 0.20 | 99.69 | 0.00 | 0.00 | 4.44 | 0.26 | 0.06 | 1.23 | 1.13 | 1.51 |
| 263 | 42.64 | 2.87 | 0.23 | 2.59 | 0.25 | 15.96 | 0.22 | 0.10 | 0.81 | 0.23 | 0.05 | 0.14 | 0.65 | 2.28 |
| 264 | 0.34 | 0.13 | 0.38 | 9.84 | 0.24 | 77.21 | 0.00 | 0.00 | 2.87 | 0.54 | 0.07 | 0.32 | 1.12 | 1.33 |
| 265 | 47.27 | 5.92 | 0.20 | 4.12 | 0.36 | 11.43 | 0.65 | 0.14 | 1.13 | 0.24 | 0.11 | 0.23 | 0.35 | 2.00 |
| 266 | 0.27 | 0.11 | 0.18 | 3.73 | 0.20 | 85.76 | 0.11 | 0.00 | 0.62 | 0.29 | 0.12 | 2.64 | 1.15 | 1.11 |
| 267 | 0.37 | 0.14 | 0.60 | 10.42 | 0.36 | 95.29 | 0.00 | 0.00 | 6.47 | 0.74 | 0.09 | 0.53 | 1.17 | 2.24 |
| 268 | 0.33 | 0.11 | 0.44 | 9.06 | 0.38 | 110.03 | 0.11 | 0.00 | 2.69 | 0.53 | 0.34 | 3.31 | 1.44 | 1.98 |
| 269 | 40.74 | 2.02 | 0.12 | 1.61 | 0.22 | 14.85 | 0.19 | 0.09 | 0.46 | 0.14 | 0.04 | 0.10 | 0.57 | 2.12 |
| 270 | 66.62 | 5.94 | 0.23 | 7.60 | 0.54 | 28.68 | 0.33 | 0.11 | 2.54 | 0.60 | 0.09 | 0.27 | 0.44 | 3.50 |
| 271 | 72.1 | 4.52 | 0.12 | 3.55 | 0.32 | 23.24 | 0.31 | 0.10 | 1.07 | 0.23 | 0.05 | 0.18 | 0.44 | 3.74 |
| 272 | 0.4 | 0.14 | 0.55 | 11.58 | 0.41 | 92.83 | 0.00 | 0.00 | 2.82 | 1.67 | 0.12 | 0.56 | 0.93 | 1.93 |
| 273 | 0.3 | 0.11 | 0.45 | 5.19 | 0.45 | 89.63 | 0.11 | 0.00 | 1.52 | 0.22 | 0.20 | 3.55 | 2.15 | 3.33 |
| 274 | 62.91 | 5.57 | 0.28 | 7.30 | 0.51 | 32.49 | 0.26 | 0.10 | 1.04 | 0.41 | 0.08 | 0.24 | 0.53 | 3.77 |
| 275 | 66.29 | 3.00 | 0.11 | 2.60 | 0.26 | 25.98 | 0.15 | 0.09 | 0.44 | 0.26 | 0.04 | 0.07 | 0.52 | 3.67 |
| 276 | 0.34 | 0.13 | 0.26 | 12.29 | 0.36 | 97.11 | 0.12 | 0.00 | 4.97 | 1.02 | 0.94 | 4.69 | 0.71 | 1.77 |
| 277 | 0.29 | 0.12 | 0.48 | 6.87 | 0.28 | 85.75 | 0.00 | 0.00 | 1.16 | 0.64 | 0.07 | 0.58 | 1.30 | 1.55 |
| 278 | 0.46 | 0.14 | 0.25 | 7.76 | 0.26 | 87.37 | 0.00 | 0.00 | 0.95 | 0.51 | 0.08 | 0.50 | 1.07 | 1.44 |
| 279 | 0.22 | 0.12 | 0.67 | 8.81 | 0.24 | 72.30 | 0.00 | 0.00 | 0.68 | 0.41 | 0.33 | 2.29 | 1.11 | 0.93 |
| 280 | 0.29 | 0.12 | 0.46 | 10.80 | 0.33 | 102.99 | 0.00 | 0.00 | 3.32 | 0.68 | 0.10 | 0.51 | 0.97 | 2.00 |
| 281 | 0.25 | 0.11 | 0.29 | 3.75 | 0.24 | 85.53 | 0.00 | 0.00 | 1.90 | 0.20 | 0.05 | 0.58 | 2.18 | 1.74 |
| 282 | 0.34 | 0.12 | 0.27 | 8.21 | 0.64 | 131.32 | 0.00 | 0.00 | 2.77 | 0.61 | 0.08 | 0.90 | 1.59 | 4.31 |
| 283 | 0.45 | 0.00 | 0.16 | 5.11 | 0.23 | 131.66 | 0.00 | 0.00 | 1.65 | 0.75 | 0.06 | 0.86 | 1.17 | 1.87 |

| | | | | | | | | | | | | | | |
|---|---|---|---|---|---|---|---|---|---|---|---|---|---|---|
| 284 | 0.39 | 0.14 | 0.34 | 10.18 | 0.34 | 108.73 | 0.00 | 0.00 | 6.56 | 0.73 | 0.10 | 0.60 | 1.14 | 2.44 |
| 285 | 53.95 | 2.62 | 0.16 | 2.98 | 0.25 | 26.66 | 0.24 | 0.09 | 1.45 | 0.50 | 0.05 | 0.21 | 0.74 | 3.14 |
| 286 | 0.29 | 0.00 | 0.07 | 3.35 | 0.23 | 104.22 | 0.00 | 0.00 | 7.89 | 0.41 | 0.05 | 0.68 | 1.02 | 2.14 |
| 287 | 72.64 | 6.44 | 0.15 | 5.24 | 0.45 | 21.60 | 0.32 | 0.11 | 1.08 | 0.28 | 0.06 | 0.17 | 0.35 | 3.67 |
| 288 | 50.1 | 0.95 | 0.04 | 0.85 | 0.20 | 23.66 | 0.23 | 0.09 | 0.61 | 0.21 | 0.04 | 0.18 | 0.50 | 2.77 |
| 289 | 48.58 | 1.24 | 0.01 | 1.05 | 0.21 | 17.77 | 0.19 | 0.09 | 2.57 | 0.33 | 0.04 | 0.11 | 0.15 | 3.11 |
| 290 | 0.27 | 0.11 | 0.20 | 4.01 | 0.24 | 78.51 | 0.00 | 0.00 | 2.79 | 0.54 | 0.05 | 0.40 | 1.43 | 1.93 |
| 291 | 0.35 | 0.11 | 0.11 | 3.17 | 0.20 | 94.29 | 0.00 | 0.00 | 0.55 | 0.65 | 0.11 | 2.61 | 0.94 | 1.31 |
| 292 | 0.26 | 0.12 | 0.12 | 4.64 | 0.21 | 79.57 | 0.00 | 0.00 | 1.55 | 0.52 | 0.05 | 0.46 | 1.06 | 1.07 |
| 293 | 48.99 | 4.62 | 0.25 | 5.91 | 0.39 | 26.95 | 0.21 | 0.10 | 2.04 | 0.36 | 0.07 | 0.19 | 0.50 | 2.99 |
| 294 | 57.63 | 1.48 | 0.04 | 1.19 | 0.21 | 22.73 | 0.29 | 0.09 | 0.87 | 0.21 | 0.04 | 0.20 | 0.44 | 2.72 |
| 295 | 0.3 | 0.11 | 0.21 | 3.83 | 0.29 | 52.00 | 0.00 | 0.00 | 2.61 | 0.31 | 0.04 | 0.17 | 1.28 | 2.58 |
| 296 | 0.33 | 0.00 | 0.15 | 3.52 | 0.24 | 92.86 | 0.00 | 0.00 | 0.70 | 0.49 | 0.13 | 3.23 | 1.28 | 3.42 |
| 297 | 0.25 | 0.11 | 0.50 | 5.18 | 0.23 | 56.85 | 0.00 | 0.00 | 0.29 | 0.19 | 0.10 | 0.90 | 1.51 | 1.15 |
| 298 | 48.28 | 1.82 | 0.05 | 1.11 | 0.21 | 19.17 | 0.14 | 0.00 | 0.30 | 0.14 | 0.03 | 0.06 | 0.37 | 2.98 |
| 299 | 0.29 | 0.12 | 0.46 | 7.45 | 0.27 | 77.13 | 0.00 | 0.00 | 2.84 | 0.50 | 0.11 | 0.92 | 1.37 | 1.44 |
| 300 | 0.4 | 0.13 | 0.19 | 6.86 | 0.25 | 114.13 | 0.00 | 0.00 | 3.45 | 1.01 | 0.08 | 1.00 | 1.26 | 1.99 |
| 301 | 0.28 | 0.12 | 0.46 | 7.23 | 0.27 | 62.52 | 0.00 | 0.00 | 1.40 | 0.39 | 0.06 | 0.21 | 0.76 | 1.42 |
| 302 | 64.44 | 7.27 | 0.25 | 6.80 | 0.64 | 20.73 | 0.16 | 0.10 | 0.89 | 0.20 | 0.05 | 0.08 | 0.35 | 3.50 |
| 303 | 62.79 | 4.62 | 0.29 | 5.19 | 0.44 | 27.71 | 0.15 | 0.10 | 0.72 | 0.28 | 0.04 | 0.09 | 0.65 | 4.06 |
| 304 | 76.12 | 1.47 | 0.06 | 1.35 | 0.21 | 34.14 | 0.29 | 0.09 | 1.04 | 0.51 | 0.04 | 0.23 | 0.70 | 3.85 |
| 305 | 63.75 | 1.80 | 0.08 | 1.23 | 0.21 | 28.62 | 0.27 | 0.09 | 1.19 | 0.23 | 0.04 | 0.21 | 0.65 | 3.48 |
| 306 | 0.35 | 0.12 | 0.40 | 7.19 | 0.25 | 87.38 | 0.11 | 0.00 | 0.35 | 0.50 | 0.34 | 3.13 | 1.11 | 1.77 |
| 307 | 0.3 | 0.11 | 0.60 | 7.68 | 0.27 | 97.60 | 0.00 | 0.00 | 2.30 | 0.76 | 0.07 | 0.54 | 1.72 | 1.67 |
| 308 | 0.34 | 0.12 | 0.48 | 8.37 | 0.28 | 100.94 | 0.00 | 0.00 | 0.82 | 0.59 | 0.08 | 0.55 | 1.34 | 1.60 |
| 309 | 0.33 | 0.12 | 0.63 | 11.77 | 0.55 | 116.64 | 0.11 | 0.00 | 2.63 | 0.40 | 0.57 | 3.78 | 1.43 | 2.69 |
| 310 | 0.29 | 0.11 | 0.36 | 5.77 | 0.22 | 81.36 | 0.00 | 0.00 | 2.09 | 0.44 | 0.05 | 0.42 | 1.71 | 1.16 |
| 311 | 0.3 | 0.12 | 0.35 | 12.26 | 0.42 | 88.95 | 0.12 | 0.00 | 4.51 | 0.89 | 0.88 | 4.17 | 0.76 | 1.86 |
| 312 | 0.23 | 0.12 | 0.40 | 7.50 | 0.49 | 59.07 | 0.00 | 0.00 | 2.00 | 0.50 | 0.05 | 0.12 | 0.93 | 2.37 |

| | | | | | | | | | | | | | | |
|---|---|---|---|---|---|---|---|---|---|---|---|---|---|---|
| 313 | 0.36 | 0.12 | 0.19 | 3.80 | 0.21 | 101.51 | 0.00 | 0.00 | 2.54 | 0.65 | 0.05 | 0.54 | 1.20 | 1.55 |
| 314 | 53.84 | 3.78 | 0.16 | 3.42 | 0.33 | 18.86 | 0.14 | 0.09 | 0.32 | 0.16 | 0.04 | 0.06 | 0.46 | 3.30 |
| 315 | 66.76 | 1.69 | 0.07 | 1.48 | 0.21 | 30.22 | 0.32 | 0.09 | 0.57 | 0.36 | 0.04 | 0.25 | 0.56 | 3.66 |
| 316 | 58.91 | 3.79 | 0.26 | 4.54 | 0.31 | 28.15 | 0.29 | 0.10 | 1.14 | 0.40 | 0.06 | 0.26 | 0.70 | 3.30 |
| 317 | 0.31 | 0.11 | 0.25 | 4.79 | 0.24 | 122.31 | 0.00 | 0.00 | 1.97 | 0.35 | 0.05 | 0.57 | 1.46 | 2.85 |
| 318 | 0.35 | 0.11 | 0.10 | 2.73 | 0.22 | 125.36 | 0.00 | 0.00 | 2.41 | 0.49 | 0.05 | 1.02 | 1.07 | 2.57 |
| 319 | 17.14 | 4.66 | 0.02 | 0.31 | 0.25 | 0.82 | 0.33 | 0.14 | 0.00 | 0.00 | 0.04 | 0.01 | 0.05 | 0.51 |
| 320 | 43.87 | 1.35 | 0.05 | 0.88 | 0.20 | 17.72 | 0.24 | 0.09 | 0.98 | 0.20 | 0.04 | 0.16 | 0.51 | 2.13 |
| 321 | 65.03 | 1.45 | 0.07 | 1.65 | 0.22 | 34.05 | 0.22 | 0.09 | 1.44 | 0.40 | 0.04 | 0.19 | 0.83 | 3.73 |
| 322 | 0.31 | 0.13 | 0.52 | 9.95 | 0.37 | 64.20 | 0.00 | 0.00 | 1.39 | 0.61 | 0.11 | 0.44 | 0.84 | 1.41 |
| 323 | 66.71 | 1.95 | 0.07 | 1.87 | 0.23 | 33.53 | 0.16 | 0.09 | 0.58 | 0.23 | 0.04 | 0.10 | 0.64 | 3.98 |
| 324 | 0.3 | 0.00 | 0.05 | 1.73 | 0.20 | 82.11 | 0.00 | 0.00 | 1.25 | 0.30 | 0.04 | 0.17 | 0.94 | 2.79 |
| 325 | 0.38 | 0.11 | 0.14 | 3.45 | 0.22 | 118.54 | 0.11 | 0.00 | 2.38 | 0.27 | 0.10 | 2.59 | 1.15 | 2.83 |
| 326 | 0.25 | 0.11 | 0.19 | 3.34 | 0.38 | 80.47 | 0.00 | 0.00 | 2.15 | 0.23 | 0.04 | 0.28 | 1.96 | 4.22 |
| 327 | 79.39 | 7.10 | 0.31 | 8.13 | 0.66 | 32.69 | 0.38 | 0.11 | 2.24 | 0.49 | 0.10 | 0.30 | 0.47 | 4.24 |
| 328 | 0.36 | 0.13 | 0.28 | 5.19 | 0.21 | 73.01 | 0.00 | 0.00 | 1.41 | 0.43 | 0.05 | 0.39 | 1.22 | 0.95 |
| 329 | 0.34 | 0.12 | 0.30 | 7.39 | 0.27 | 91.94 | 0.00 | 0.00 | 4.82 | 0.44 | 0.09 | 0.70 | 1.12 | 2.17 |
| 330 | 27.12 | 3.27 | 0.19 | 2.61 | 0.25 | 9.92 | 0.17 | 0.10 | 0.49 | 0.15 | 0.05 | 0.08 | 0.41 | 1.34 |
| 331 | 0.31 | 0.13 | 0.65 | 12.07 | 0.59 | 71.17 | 0.00 | 0.00 | 2.47 | 0.87 | 0.42 | 1.73 | 1.01 | 2.26 |
| 332 | 0.24 | 0.11 | 0.56 | 8.39 | 0.31 | 69.00 | 0.00 | 0.00 | 1.73 | 0.28 | 0.07 | 0.32 | 1.14 | 1.96 |
| 333 | 74.14 | 5.33 | 0.22 | 4.10 | 0.38 | 21.70 | 0.33 | 0.10 | 0.97 | 0.28 | 0.06 | 0.17 | 0.53 | 3.73 |
| 334 | 0.32 | 0.11 | 0.16 | 4.12 | 0.21 | 85.89 | 0.11 | 0.00 | 0.28 | 0.52 | 0.16 | 3.00 | 1.01 | 1.60 |
| 335 | 66.67 | 4.57 | 0.23 | 5.00 | 0.36 | 27.68 | 0.30 | 0.10 | 1.02 | 0.28 | 0.06 | 0.21 | 0.59 | 3.65 |
| 336 | 60.34 | 2.86 | 0.11 | 2.64 | 0.26 | 25.94 | 0.33 | 0.10 | 1.82 | 0.39 | 0.05 | 0.27 | 0.53 | 3.11 |
| 337 | 0.28 | 0.12 | 0.32 | 7.22 | 0.36 | 72.26 | 0.00 | 0.00 | 4.32 | 0.53 | 0.06 | 0.31 | 0.98 | 3.32 |
| 338 | 61.77 | 1.64 | 0.08 | 1.61 | 0.21 | 31.12 | 0.27 | 0.09 | 0.94 | 0.26 | 0.04 | 0.23 | 0.73 | 3.29 |
| 339 | 0.26 | 0.12 | 0.19 | 6.22 | 0.34 | 69.28 | 0.00 | 0.00 | 2.43 | 1.12 | 0.06 | 0.30 | 0.94 | 2.75 |
| 340 | 0.36 | 0.12 | 0.72 | 11.38 | 0.43 | 92.41 | 0.00 | 0.00 | 3.07 | 1.91 | 0.13 | 0.73 | 1.14 | 1.72 |
| 341 | 0.25 | 0.00 | 0.45 | 6.06 | 0.24 | 78.11 | 0.00 | 0.00 | 0.93 | 0.56 | 0.07 | 0.53 | 1.18 | 1.18 |

| | | | | | | | | | | | | | | |
|---|---|---|---|---|---|---|---|---|---|---|---|---|---|---|
| 342 | 0.28 | 0.11 | 0.21 | 4.07 | 0.34 | 71.67 | 0.00 | 0.00 | 1.28 | 0.30 | 0.04 | 0.17 | 1.42 | 3.27 |
| 343 | 0.22 | 0.12 | 0.52 | 9.12 | 0.45 | 53.62 | 0.00 | 0.00 | 1.91 | 0.28 | 0.08 | 0.24 | 0.99 | 2.42 |
| 344 | 13.26 | 1.20 | 0.11 | 1.32 | 0.21 | 6.09 | 0.54 | 0.11 | 0.53 | 0.14 | 0.10 | 0.37 | 0.33 | 0.85 |
| 345 | 0.39 | 0.11 | 0.11 | 3.42 | 0.22 | 111.64 | 0.00 | 0.00 | 3.09 | 0.33 | 0.04 | 0.47 | 1.39 | 2.60 |
| 346 | 65.87 | 4.42 | 0.27 | 3.88 | 0.32 | 24.00 | 0.34 | 0.10 | 1.39 | 0.36 | 0.06 | 0.24 | 0.80 | 3.24 |
| 347 | 0.36 | 0.11 | 0.20 | 3.36 | 0.33 | 88.33 | 0.00 | 0.00 | 2.48 | 0.24 | 0.05 | 0.58 | 2.14 | 2.40 |
| 348 | 0.24 | 0.12 | 0.43 | 9.12 | 1.21 | 65.34 | 0.00 | 0.00 | 0.54 | 0.54 | 0.37 | 2.02 | 1.43 | 3.59 |
| 349 | 0.22 | 0.12 | 0.66 | 7.19 | 0.36 | 63.24 | 0.00 | 0.00 | 2.11 | 0.34 | 0.04 | 0.09 | 1.15 | 2.01 |
| 350 | 0.25 | 0.12 | 0.30 | 4.93 | 0.24 | 80.90 | 0.11 | 0.00 | 3.08 | 0.52 | 0.22 | 3.62 | 1.40 | 1.48 |
| 351 | 0.38 | 0.12 | 0.54 | 9.48 | 0.35 | 118.31 | 0.00 | 0.00 | 2.49 | 0.88 | 0.11 | 0.88 | 1.57 | 2.08 |
| 352 | 0.33 | 0.11 | 0.11 | 4.03 | 0.29 | 114.33 | 0.11 | 0.00 | 2.61 | 0.32 | 0.17 | 4.59 | 0.93 | 3.62 |
| 353 | 0.25 | 0.12 | 0.24 | 3.87 | 0.23 | 74.87 | 0.00 | 0.00 | 2.81 | 0.51 | 0.04 | 0.31 | 1.71 | 2.13 |
| 354 | 0.23 | 0.11 | 0.38 | 6.77 | 0.29 | 68.71 | 0.00 | 0.00 | 0.62 | 0.52 | 0.06 | 0.24 | 1.03 | 1.55 |
| 355 | 0.27 | 0.13 | 0.63 | 10.15 | 0.43 | 75.32 | 0.00 | 0.00 | 2.70 | 0.95 | 0.31 | 1.84 | 1.16 | 2.02 |
| 356 | 0.27 | 0.13 | 0.70 | 9.98 | 0.25 | 83.59 | 0.00 | 0.00 | 4.88 | 0.52 | 0.16 | 1.05 | 1.19 | 1.35 |
| 357 | 29.81 | 1.61 | 0.05 | 1.09 | 0.21 | 12.06 | 1.58 | 0.13 | 0.40 | 0.14 | 0.11 | 1.11 | 0.28 | 2.03 |
| 358 | 0.28 | 0.11 | 0.37 | 6.96 | 0.27 | 105.39 | 0.00 | 0.00 | 3.13 | 0.30 | 0.08 | 0.66 | 1.87 | 1.83 |
| 359 | 0.23 | 0.13 | 0.83 | 12.45 | 0.49 | 69.91 | 0.00 | 0.00 | 1.61 | 0.42 | 0.10 | 0.34 | 0.95 | 2.18 |
| 360 | 0.29 | 0.12 | 0.36 | 6.97 | 0.24 | 102.84 | 0.00 | 0.00 | 2.04 | 0.66 | 0.08 | 0.74 | 1.67 | 1.56 |
| 361 | 0.34 | 0.00 | 0.16 | 4.13 | 0.21 | 109.27 | 0.00 | 0.00 | 2.06 | 0.57 | 0.06 | 0.73 | 1.26 | 1.54 |
| 362 | 0.28 | 0.11 | 0.24 | 4.87 | 0.21 | 102.96 | 0.00 | 0.00 | 0.34 | 0.34 | 0.13 | 2.60 | 1.11 | 1.41 |
| 363 | 0.32 | 0.13 | 0.47 | 8.06 | 0.40 | 103.87 | 0.00 | 0.00 | 2.35 | 1.31 | 0.07 | 0.44 | 1.92 | 2.86 |
| 364 | 0.29 | 0.12 | 0.41 | 7.12 | 0.27 | 81.96 | 0.11 | 0.00 | 2.48 | 0.56 | 0.37 | 3.74 | 1.32 | 1.54 |
| 365 | 0.28 | 0.00 | 0.37 | 5.78 | 0.24 | 91.49 | 0.00 | 0.00 | 0.83 | 0.48 | 0.06 | 0.48 | 1.47 | 1.49 |
| 366 | 49.4 | 3.90 | 0.18 | 2.62 | 0.29 | 14.12 | 0.23 | 0.10 | 1.38 | 0.21 | 0.05 | 0.11 | 0.51 | 2.60 |
| 367 | 0.32 | 0.12 | 0.41 | 4.80 | 0.24 | 94.36 | 0.00 | 0.00 | 1.91 | 0.72 | 0.05 | 0.42 | 2.14 | 1.81 |
| 368 | 0.3 | 0.11 | 0.19 | 2.06 | 0.20 | 61.16 | 0.00 | 0.00 | 0.97 | 0.34 | 0.04 | 0.41 | 2.03 | 0.98 |
| 369 | 62.18 | 1.58 | 0.04 | 2.43 | 0.24 | 38.65 | 0.25 | 0.09 | 1.28 | 0.41 | 0.04 | 0.27 | 0.35 | 4.01 |
| 370 | 14.76 | 1.07 | 0.10 | 0.92 | 0.20 | 6.25 | 1.02 | 0.12 | 0.53 | 0.13 | 0.12 | 0.71 | 0.38 | 0.75 |

| | | | | | | | | | | | | | | |
|---|---|---|---|---|---|---|---|---|---|---|---|---|---|---|
| 371 | 1.23 | 0.23 | 0.47 | 7.40 | 0.24 | 56.33 | 0.11 | 0.00 | 6.12 | 0.73 | 0.10 | 0.57 | 1.20 | 1.21 |
| 372 | 0.31 | 0.12 | 0.30 | 6.06 | 0.29 | 99.94 | 0.00 | 0.00 | 3.18 | 0.70 | 0.06 | 0.55 | 2.04 | 2.40 |
| 373 | 0.29 | 0.12 | 0.23 | 4.79 | 0.22 | 105.75 | 0.00 | 0.00 | 3.55 | 0.53 | 0.05 | 0.38 | 1.21 | 1.60 |
| 374 | 0.35 | 0.11 | 0.15 | 4.84 | 0.22 | 125.68 | 0.00 | 0.00 | 2.97 | 0.97 | 0.05 | 0.62 | 0.95 | 1.97 |
| 375 | 0.27 | 0.12 | 0.68 | 9.31 | 0.55 | 83.39 | 0.00 | 0.00 | 1.44 | 0.40 | 0.33 | 2.29 | 1.71 | 2.58 |
| 376 | 0.27 | 0.12 | 0.35 | 6.80 | 0.27 | 85.08 | 0.00 | 0.00 | 2.94 | 0.61 | 0.06 | 0.41 | 1.73 | 1.65 |
| 377 | 0.35 | 0.11 | 0.32 | 6.07 | 0.24 | 148.84 | 0.00 | 0.00 | 2.74 | 0.42 | 0.06 | 0.61 | 1.41 | 2.05 |
| 378 | 0.36 | 0.00 | 0.13 | 6.07 | 0.24 | 112.20 | 0.00 | 0.00 | 0.57 | 0.99 | 0.06 | 0.63 | 0.74 | 2.00 |
| 379 | 0.28 | 0.00 | 0.12 | 3.23 | 0.20 | 106.31 | 0.00 | 0.00 | 1.72 | 0.56 | 0.04 | 0.58 | 1.41 | 1.28 |
| 380 | 60.5 | 2.55 | 0.12 | 3.11 | 0.27 | 32.84 | 0.21 | 0.09 | 1.26 | 0.45 | 0.04 | 0.19 | 0.63 | 3.59 |
| 381 | 0.31 | 0.13 | 0.65 | 9.36 | 0.32 | 99.37 | 0.00 | 0.00 | 3.79 | 0.68 | 0.06 | 0.33 | 1.36 | 2.15 |
| 382 | 83.37 | 2.76 | 0.06 | 2.98 | 0.28 | 40.04 | 0.39 | 0.10 | 2.23 | 0.45 | 0.05 | 0.38 | 0.51 | 4.44 |
| 383 | 0.38 | 0.00 | 0.10 | 5.49 | 0.27 | 136.29 | 0.00 | 0.00 | 4.17 | 0.81 | 0.06 | 0.79 | 1.01 | 2.55 |
| 384 | 0.36 | 0.12 | 0.12 | 5.55 | 0.44 | 114.91 | 0.00 | 0.00 | 3.78 | 0.75 | 0.05 | 0.54 | 1.05 | 4.47 |
| 385 | 62.43 | 1.74 | 0.09 | 1.83 | 0.22 | 30.96 | 0.26 | 0.09 | 1.03 | 0.28 | 0.04 | 0.21 | 0.75 | 3.39 |
| 386 | 0.33 | 0.12 | 0.63 | 9.41 | 0.65 | 95.89 | 0.11 | 0.00 | 1.49 | 0.49 | 0.40 | 3.03 | 1.62 | 3.36 |
| 387 | 0.3 | 0.00 | 0.26 | 4.99 | 0.21 | 100.10 | 0.11 | 0.00 | 0.35 | 0.29 | 0.12 | 2.32 | 1.16 | 1.46 |
| 388 | 44.71 | 1.47 | 0.08 | 2.25 | 0.26 | 31.39 | 1.39 | 0.12 | 1.72 | 0.19 | 0.14 | 1.84 | 0.58 | 3.60 |
| 389 | 0.33 | 0.12 | 0.70 | 8.93 | 0.43 | 99.25 | 0.11 | 0.00 | 1.44 | 0.35 | 0.36 | 3.25 | 1.71 | 2.38 |
| 390 | 38.49 | 2.14 | 0.07 | 1.61 | 0.23 | 14.19 | 1.81 | 0.13 | 0.55 | 0.15 | 0.12 | 1.22 | 0.33 | 2.58 |
| 391 | 0.33 | 0.11 | 0.12 | 3.15 | 0.21 | 130.84 | 0.00 | 0.00 | 2.21 | 0.59 | 0.05 | 0.87 | 1.28 | 1.58 |
| 392 | 75.01 | 2.13 | 0.06 | 1.62 | 0.24 | 24.58 | 0.32 | 0.09 | 3.32 | 0.29 | 0.04 | 0.19 | 0.51 | 3.89 |
| 393 | 19.83 | 2.28 | 0.16 | 2.36 | 0.24 | 9.97 | 0.68 | 0.13 | 0.77 | 0.17 | 0.15 | 0.57 | 0.37 | 1.13 |
| 394 | 0.33 | 0.11 | 0.14 | 3.88 | 0.26 | 81.97 | 0.00 | 0.00 | 2.99 | 1.10 | 0.04 | 0.33 | 0.96 | 2.85 |
| 395 | 0.23 | 0.11 | 0.40 | 5.59 | 0.25 | 43.98 | 0.00 | 0.00 | 0.39 | 0.35 | 0.05 | 0.15 | 1.08 | 1.28 |
| 396 | 0.25 | 0.11 | 0.19 | 3.81 | 0.20 | 81.23 | 0.11 | 0.00 | 0.74 | 0.31 | 0.13 | 2.60 | 1.01 | 1.08 |
| 397 | 29.07 | 4.88 | 0.29 | 5.55 | 0.50 | 12.04 | 0.16 | 0.10 | 0.40 | 0.17 | 0.06 | 0.09 | 0.31 | 2.56 |
| 398 | 0.23 | 0.11 | 0.42 | 6.97 | 0.55 | 73.92 | 0.00 | 0.00 | 3.33 | 0.64 | 0.05 | 0.15 | 1.21 | 3.82 |
| 399 | 0.33 | 0.12 | 0.74 | 9.53 | 0.28 | 88.30 | 0.00 | 0.00 | 4.24 | 0.50 | 0.11 | 0.67 | 1.41 | 1.52 |

| | | | | | | | | | | | | | | |
|---|---|---|---|---|---|---|---|---|---|---|---|---|---|---|
| 400 | 0.29 | 0.12 | 1.09 | 14.49 | 1.15 | 89.31 | 0.00 | 0.00 | 0.85 | 0.81 | 0.78 | 3.04 | 1.36 | 3.85 |
| 401 | 0.21 | 0.11 | 0.41 | 6.96 | 0.69 | 68.89 | 0.00 | 0.00 | 2.32 | 0.26 | 0.08 | 0.43 | 1.91 | 3.30 |
| 402 | 0.28 | 0.11 | 0.26 | 4.63 | 0.22 | 95.33 | 0.00 | 0.00 | 1.21 | 0.51 | 0.05 | 0.45 | 2.05 | 1.35 |
| 403 | 0.33 | 0.11 | 0.51 | 8.33 | 0.33 | 103.13 | 0.00 | 0.00 | 2.70 | 1.42 | 0.08 | 0.70 | 1.52 | 1.92 |
| 404 | 0.27 | 0.11 | 0.54 | 6.64 | 0.24 | 84.58 | 0.00 | 0.00 | 1.36 | 0.46 | 0.24 | 2.82 | 1.41 | 1.20 |
| 405 | 0.32 | 0.11 | 0.11 | 6.35 | 0.24 | 118.53 | 0.00 | 0.00 | 1.21 | 1.29 | 0.07 | 0.66 | 0.67 | 1.66 |
| 406 | 66.59 | 6.36 | 0.34 | 6.53 | 0.51 | 23.57 | 0.32 | 0.11 | 1.06 | 0.56 | 0.07 | 0.21 | 0.54 | 3.48 |
| 407 | 67.4 | 5.07 | 0.17 | 4.36 | 0.39 | 18.40 | 0.31 | 0.10 | 3.34 | 0.43 | 0.06 | 0.15 | 0.41 | 3.34 |
| 408 | 68.11 | 6.22 | 0.34 | 6.71 | 0.49 | 26.48 | 0.30 | 0.11 | 1.51 | 0.32 | 0.08 | 0.20 | 0.55 | 3.59 |
| 409 | 0.42 | 0.11 | 0.42 | 7.67 | 0.28 | 92.22 | 0.11 | 0.00 | 0.48 | 0.63 | 0.33 | 3.30 | 1.18 | 1.94 |
| 410 | 73.01 | 6.45 | 0.29 | 5.70 | 0.50 | 22.18 | 0.16 | 0.10 | 0.81 | 0.19 | 0.05 | 0.07 | 0.50 | 3.71 |
| 411 | 76.43 | 6.34 | 0.40 | 7.64 | 0.60 | 35.16 | 0.17 | 0.10 | 1.16 | 0.44 | 0.06 | 0.12 | 0.57 | 4.04 |
| 412 | 0.4 | 0.14 | 0.64 | 10.56 | 0.94 | 85.32 | 0.00 | 0.00 | 4.59 | 0.68 | 0.09 | 0.38 | 1.23 | 3.12 |
| 413 | 0.31 | 0.11 | 0.28 | 7.57 | 0.28 | 75.91 | 0.00 | 0.00 | 3.40 | 0.46 | 0.04 | 0.11 | 0.92 | 2.72 |
| 414 | 40.88 | 3.60 | 0.25 | 3.82 | 0.29 | 20.43 | 1.20 | 0.14 | 2.74 | 0.34 | 0.21 | 1.11 | 0.50 | 2.23 |
| 415 | 54.85 | 5.41 | 0.24 | 4.12 | 0.39 | 15.37 | 0.25 | 0.10 | 0.73 | 0.23 | 0.05 | 0.12 | 0.53 | 2.81 |
| 416 | 0.5 | 0.13 | 0.63 | 9.37 | 0.27 | 87.77 | 0.00 | 0.00 | 1.74 | 0.54 | 0.13 | 1.01 | 1.37 | 1.52 |
| 417 | 47.31 | 2.41 | 0.07 | 1.70 | 0.23 | 16.49 | 0.82 | 0.11 | 1.37 | 0.20 | 0.07 | 0.43 | 0.41 | 2.09 |
| 418 | 0.28 | 0.11 | 0.53 | 6.09 | 0.42 | 69.90 | 0.11 | 0.00 | 0.88 | 0.18 | 0.30 | 3.19 | 2.00 | 2.41 |
| 419 | 0.3 | 0.11 | 0.29 | 6.49 | 0.41 | 108.82 | 0.00 | 0.00 | 3.31 | 1.24 | 0.06 | 0.47 | 2.17 | 3.16 |
| 420 | 0.45 | 0.14 | 0.74 | 13.52 | 0.54 | 117.71 | 0.00 | 0.00 | 5.40 | 0.98 | 0.10 | 0.43 | 1.20 | 2.66 |
| 421 | 0.27 | 0.11 | 0.07 | 2.78 | 0.20 | 88.67 | 0.00 | 0.00 | 1.80 | 0.50 | 0.04 | 0.49 | 0.65 | 1.12 |
| 422 | 59.97 | 4.24 | 0.20 | 3.56 | 0.33 | 20.53 | 0.31 | 0.10 | 1.95 | 0.42 | 0.05 | 0.20 | 0.61 | 3.17 |
| 423 | 92.26 | 5.01 | 0.30 | 6.38 | 0.50 | 46.57 | 0.40 | 0.10 | 2.84 | 0.75 | 0.07 | 0.40 | 0.75 | 4.86 |
| 424 | 0.23 | 0.11 | 0.16 | 3.12 | 0.36 | 60.99 | 0.00 | 0.00 | 3.63 | 0.26 | 0.04 | 0.22 | 1.46 | 4.56 |
| 425 | 0.33 | 0.11 | 0.38 | 8.01 | 2.24 | 40.90 | 0.00 | 0.00 | 0.75 | 0.24 | 0.08 | 0.21 | 1.04 | 4.33 |
| 426 | 44.42 | 3.89 | 0.21 | 3.92 | 0.36 | 16.83 | 0.22 | 0.10 | 1.31 | 0.25 | 0.06 | 0.15 | 0.50 | 3.17 |
| 427 | 42.91 | 5.01 | 0.23 | 4.58 | 0.43 | 13.37 | 0.13 | 0.09 | 0.77 | 0.23 | 0.04 | 0.04 | 0.38 | 2.87 |
| 428 | 0.59 | 0.12 | 0.63 | 9.03 | 0.48 | 150.49 | 0.00 | 0.00 | 2.86 | 0.54 | 0.07 | 0.59 | 2.19 | 2.53 |

| | | | | | | | | | | | | | | |
|---|---|---|---|---|---|---|---|---|---|---|---|---|---|---|
| 429 | 55.98 | 2.84 | 0.11 | 4.96 | 0.43 | 41.38 | 1.68 | 0.13 | 2.07 | 0.34 | 0.24 | 2.11 | 0.34 | 4.97 |
| 430 | 0.3 | 0.11 | 0.30 | 6.99 | 0.26 | 91.64 | 0.00 | 0.00 | 3.51 | 0.92 | 0.06 | 0.33 | 1.22 | 1.69 |
| 431 | 41.38 | 2.65 | 0.06 | 2.40 | 0.25 | 16.88 | 0.19 | 0.10 | 1.07 | 0.21 | 0.05 | 0.13 | 0.24 | 2.40 |
| 432 | 0.24 | 0.11 | 0.35 | 8.47 | 0.40 | 46.78 | 0.00 | 0.00 | 2.50 | 0.53 | 0.05 | 0.08 | 0.79 | 2.17 |
| 433 | 0.33 | 0.11 | 0.04 | 3.33 | 0.23 | 117.53 | 0.00 | 0.00 | 9.53 | 0.50 | 0.05 | 0.83 | 0.85 | 2.70 |
| 434 | 0.3 | 0.11 | 0.03 | 1.40 | 0.20 | 92.26 | 0.00 | 0.00 | 3.26 | 0.39 | 0.04 | 0.48 | 0.50 | 1.41 |
| 435 | 0.24 | 0.12 | 0.74 | 11.20 | 0.28 | 62.65 | 0.00 | 0.00 | 0.76 | 0.35 | 0.17 | 0.70 | 1.14 | 0.84 |
| 436 | 61.56 | 0.87 | 0.02 | 0.64 | 0.20 | 23.75 | 0.32 | 0.00 | 1.68 | 0.29 | 0.04 | 0.22 | 0.33 | 3.20 |
| 437 | 36.45 | 1.18 | 0.03 | 1.21 | 0.21 | 16.44 | 1.26 | 0.11 | 1.39 | 0.19 | 0.09 | 1.06 | 0.24 | 2.57 |
| 438 | 0.31 | 0.12 | 0.36 | 8.10 | 0.32 | 102.36 | 0.00 | 0.00 | 4.09 | 0.89 | 0.07 | 0.49 | 1.72 | 1.93 |
| 439 | 59.41 | 7.74 | 0.36 | 7.82 | 0.69 | 19.33 | 0.29 | 0.11 | 1.09 | 0.63 | 0.09 | 0.18 | 0.48 | 3.47 |
| 440 | 0.27 | 0.11 | 0.82 | 11.20 | 0.25 | 48.31 | 0.00 | 0.00 | 0.23 | 0.25 | 0.26 | 0.82 | 1.02 | 0.99 |
| 441 | 0.33 | 0.11 | 0.19 | 7.14 | 0.23 | 121.46 | 0.00 | 0.00 | 0.76 | 0.55 | 0.18 | 2.82 | 0.98 | 1.67 |
| 442 | 0.27 | 0.11 | 0.84 | 9.23 | 0.25 | 90.62 | 0.00 | 0.00 | 1.17 | 0.63 | 0.33 | 2.66 | 1.44 | 1.21 |
| 443 | 50.69 | 5.60 | 0.30 | 4.56 | 0.43 | 15.43 | 0.14 | 0.10 | 0.26 | 0.24 | 0.04 | 0.04 | 0.58 | 2.90 |
| 444 | 0.3 | 0.11 | 0.57 | 7.53 | 0.52 | 87.87 | 0.00 | 0.00 | 3.11 | 0.50 | 0.07 | 0.48 | 1.88 | 3.18 |
| 445 | 0.28 | 0.11 | 0.33 | 6.12 | 0.24 | 101.22 | 0.00 | 0.00 | 2.15 | 0.62 | 0.07 | 0.67 | 1.52 | 1.53 |
| 446 | 0.31 | 0.11 | 0.73 | 9.02 | 0.40 | 111.40 | 0.11 | 0.00 | 3.20 | 0.67 | 0.37 | 3.30 | 1.96 | 2.44 |
| 447 | 0.28 | 0.13 | 0.81 | 11.71 | 0.37 | 81.68 | 0.00 | 0.00 | 3.05 | 0.68 | 0.08 | 0.29 | 1.38 | 2.07 |
| 448 | 0.31 | 0.11 | 0.11 | 5.22 | 0.27 | 92.13 | 0.00 | 0.00 | 2.80 | 1.58 | 0.06 | 0.68 | 0.78 | 1.80 |
| 449 | 0.28 | 0.13 | 0.82 | 11.53 | 0.39 | 84.30 | 0.00 | 0.00 | 3.13 | 0.72 | 0.08 | 0.30 | 1.44 | 2.07 |
| 450 | 0.24 | 0.11 | 0.43 | 6.90 | 0.54 | 82.38 | 0.00 | 0.00 | 0.75 | 0.67 | 0.27 | 2.61 | 1.83 | 3.38 |
| 451 | 58 | 5.21 | 0.23 | 4.53 | 0.48 | 19.21 | 0.15 | 0.09 | 0.57 | 0.18 | 0.04 | 0.06 | 0.46 | 3.67 |
| 452 | 0.28 | 0.11 | 0.05 | 3.67 | 0.21 | 88.28 | 0.00 | 0.00 | 3.39 | 0.57 | 0.06 | 1.07 | 0.76 | 1.77 |
| 453 | 0.45 | 0.11 | 0.13 | 5.73 | 0.30 | 136.74 | 0.00 | 0.00 | 5.96 | 2.20 | 0.07 | 1.08 | 1.24 | 2.37 |
| 454 | 62.48 | 4.21 | 0.20 | 4.36 | 0.37 | 23.14 | 0.28 | 0.10 | 1.69 | 0.40 | 0.06 | 0.18 | 0.53 | 3.63 |
| 455 | 0.45 | 0.11 | 0.17 | 4.68 | 0.22 | 100.94 | 0.11 | 0.00 | 0.63 | 0.96 | 0.05 | 0.56 | 1.05 | 1.41 |
| 456 | 0.29 | 0.10 | 0.06 | 3.25 | 0.25 | 122.63 | 0.11 | 0.00 | 3.32 | 0.34 | 0.12 | 4.67 | 1.04 | 3.49 |
| 457 | 0.33 | 0.11 | 0.25 | 6.54 | 0.41 | 134.97 | 0.00 | 0.00 | 3.08 | 0.54 | 0.06 | 0.65 | 1.63 | 3.04 |

| | | | | | | | | | | | | | | |
|---|---|---|---|---|---|---|---|---|---|---|---|---|---|---|
| 458 | 0.4 | 0.11 | 0.17 | 6.08 | 0.26 | 133.06 | 0.00 | 0.00 | 4.89 | 1.22 | 0.07 | 1.02 | 1.47 | 2.05 |
| 459 | 0.29 | 0.11 | 0.07 | 4.03 | 0.23 | 100.99 | 0.00 | 0.00 | 3.34 | 0.39 | 0.05 | 0.50 | 0.79 | 2.61 |
| 460 | 0.4 | 0.12 | 0.16 | 8.88 | 0.33 | 141.30 | 0.00 | 0.00 | 3.96 | 1.45 | 0.08 | 0.85 | 0.96 | 2.48 |
| 461 | 0.26 | 0.12 | 0.50 | 9.02 | 0.27 | 63.01 | 0.00 | 0.00 | 1.19 | 0.43 | 0.04 | 0.08 | 1.16 | 2.01 |
| 462 | 0.26 | 0.11 | 0.44 | 5.04 | 0.31 | 40.25 | 0.00 | 0.00 | 0.52 | 0.24 | 0.16 | 1.26 | 1.32 | 1.15 |
| 463 | 0.32 | 0.12 | 0.53 | 9.31 | 0.29 | 82.85 | 0.00 | 0.00 | 8.30 | 0.97 | 0.14 | 0.94 | 1.24 | 1.74 |
| 464 | 0.28 | 0.11 | 0.32 | 8.86 | 0.40 | 81.98 | 0.00 | 0.00 | 4.01 | 0.62 | 0.07 | 0.30 | 1.28 | 2.80 |
| 465 | 74.41 | 2.03 | 0.05 | 2.49 | 0.26 | 37.20 | 0.34 | 0.10 | 2.52 | 0.46 | 0.04 | 0.30 | 0.42 | 4.50 |
| 466 | 33.62 | 3.37 | 0.18 | 3.22 | 0.30 | 13.43 | 0.17 | 0.10 | 0.34 | 0.19 | 0.05 | 0.10 | 0.44 | 2.11 |
| 467 | 0.27 | 0.11 | 0.23 | 7.18 | 0.25 | 80.22 | 0.00 | 0.00 | 10.59 | 0.67 | 0.11 | 1.02 | 1.16 | 1.74 |
| 468 | 0.22 | 0.11 | 0.30 | 6.76 | 0.28 | 51.86 | 0.00 | 0.00 | 2.50 | 0.63 | 0.05 | 0.17 | 1.02 | 1.40 |
| 469 | 0.47 | 0.12 | 0.09 | 5.93 | 0.52 | 129.67 | 0.00 | 0.00 | 5.01 | 0.96 | 0.06 | 0.65 | 0.81 | 5.27 |
| 470 | 0.26 | 0.12 | 0.51 | 8.28 | 0.29 | 77.28 | 0.00 | 0.00 | 4.21 | 0.47 | 0.09 | 0.59 | 1.12 | 1.61 |
| 471 | 67.64 | 2.52 | 0.05 | 2.31 | 0.25 | 26.67 | 0.35 | 0.10 | 2.27 | 0.35 | 0.05 | 0.24 | 0.38 | 3.59 |
| 472 | 0.31 | 0.11 | 0.11 | 3.94 | 0.21 | 93.16 | 0.11 | 0.00 | 1.10 | 0.48 | 0.12 | 2.69 | 0.96 | 1.21 |
| 473 | 0.26 | 0.11 | 0.29 | 5.34 | 0.22 | 61.71 | 0.00 | 0.00 | 2.86 | 0.36 | 0.05 | 0.31 | 1.52 | 1.07 |
| 474 | 66.52 | 4.48 | 0.38 | 4.89 | 0.38 | 31.39 | 0.29 | 0.10 | 1.69 | 0.50 | 0.06 | 0.27 | 0.92 | 3.83 |
| 475 | 0.33 | 0.11 | 0.06 | 4.22 | 0.21 | 101.96 | 0.00 | 0.00 | 2.02 | 0.48 | 0.06 | 1.14 | 0.72 | 1.06 |
| 476 | 0.29 | 0.12 | 0.53 | 7.61 | 0.38 | 84.47 | 0.11 | 0.00 | 0.72 | 0.49 | 0.34 | 3.05 | 1.46 | 2.79 |
| 477 | 29.95 | 3.72 | 0.19 | 3.34 | 0.35 | 8.41 | 0.16 | 0.10 | 1.54 | 0.28 | 0.05 | 0.06 | 0.36 | 1.83 |
| 478 | 0.21 | 0.11 | 0.38 | 7.39 | 0.32 | 32.18 | 0.00 | 0.00 | 0.92 | 0.32 | 0.04 | 0.06 | 0.77 | 1.58 |
| 479 | 62.4 | 3.47 | 0.06 | 3.23 | 0.29 | 29.44 | 2.19 | 0.15 | 3.10 | 0.37 | 0.19 | 1.97 | 0.28 | 3.33 |
| 480 | 87.88 | 3.17 | 0 | 0.28 | 0.31 | 2.22 | 2.47 | 0.09 | 2.04 | 0.43 | 0.01 | 0.1 | 0.01 | 3.64 |
| 481 | 69 | 3.23 | 0 | 0.29 | 0.31 | 1.81 | 6.36 | 0.3 | 1.67 | 0.32 | 0.05 | 0.26 | 0.01 | 3.22 |
| 482 | 56.06 | 2.5 | 0 | 0.25 | 0.28 | 1.69 | 3.15 | 0.11 | 2.16 | 0.28 | 0.02 | 0.09 | 0.02 | 2.44 |
| 483 | 69.53 | 5.1 | 0.012 | 0.43 | 0.38 | 1.75 | 0.67 | 0.05 | 2.38 | 0.26 | 0.01 | 0.02 | 0.02 | 2.8 |
| 484 | 70.08 | 1.95 | 0 | 0.19 | 0.25 | 1.86 | 1.07 | 0.03 | 1.12 | 0.18 | 0.01 | 0.04 | 0.02 | 2.92 |
| 485 | 86.38 | 7.19 | 0.014 | 0.86 | 0.59 | 2.39 | 5.11 | 0.39 | 1.76 | 0.47 | 0.06 | 0.14 | 0.02 | 3.97 |
| 486 | 58.27 | 6.33 | 0.012 | 0.61 | 0.46 | 1.31 | 1.39 | 0.16 | 1.85 | 0.35 | 0.02 | 0.03 | 0.01 | 2.27 |

| | | | | | | | | | | | | | | |
|---|---|---|---|---|---|---|---|---|---|---|---|---|---|---|
| 487 | 74.57 | 7.75 | 0.018 | 0.62 | 0.74 | 1.51 | 5.37 | 0.64 | 0.69 | 0.28 | 0.08 | 0.19 | 0.02 | 4.37 |
| 488 | 94.61 | 2.28 | 0.025 | 3.24 | 0.3 | 44.64 | 0.16 | 0 | 2.06 | 0.61 | 0.01 | 0.15 | 0.04 | 5.08 |
| 489 | 87.46 | 2.81 | 0 | 0.27 | 0.3 | 2.12 | 4.58 | 0.17 | 3.82 | 0.5 | 0.03 | 0.19 | 0.02 | 3.53 |
| 490 | 97.95 | 3.25 | 0 | 0.35 | 0.38 | 3.09 | 10.76 | 0.35 | 4.07 | 0.48 | 0.06 | 0.44 | 0.02 | 6.11 |
| 491 | 64.72 | 3.64 | 0 | 0.27 | 0.31 | 1.9 | 1.16 | 0.05 | 1.27 | 0.22 | 0.01 | 0.04 | 0.01 | 3.22 |
| 492 | 71.12 | 10.01 | 0.018 | 0.9 | 0.6 | 1.22 | 0.36 | 0.06 | 1.6 | 0.34 | 0.01 | 0 | 0.02 | 2.48 |
| 493 | 99.69 | 9.57 | 0.013 | 1.12 | 0.76 | 2.42 | 1.4 | 0.13 | 2.53 | 0.46 | 0.02 | 0.04 | 0.01 | 4.15 |
| 494 | 113.97 | 6.9 | 0 | 0.69 | 0.56 | 2.52 | 3.85 | 0.25 | 4.35 | 0.54 | 0.04 | 0.14 | 0.01 | 4.26 |
| 495 | 77.35 | 1.48 | 0.021 | 2.21 | 0.24 | 41.46 | 0.29 | 0 | 1.85 | 0.5 | 0.02 | 0.26 | 0.05 | 4.15 |
| 496 | 69.44 | 6.93 | 0.01 | 0.71 | 0.49 | 1.48 | 1.59 | 0.16 | 1.86 | 0.35 | 0.02 | 0.03 | 0.01 | 2.63 |
| 497 | 51.71 | 5.27 | 0 | 0.58 | 0.4 | 1.43 | 1.48 | 0.12 | 1.31 | 0.24 | 0.02 | 0.05 | 0.01 | 2.22 |
| 498 | 81.15 | 3.24 | 0 | 0.28 | 0.3 | 1.98 | 0.95 | 0.04 | 2.92 | 0.28 | 0.01 | 0.04 | 0.04 | 3.13 |
| 499 | 96.87 | 9.27 | 0.019 | 0.82 | 0.68 | 1.95 | 2.83 | 0.28 | 1.57 | 0.5 | 0.04 | 0.08 | 0.02 | 3.55 |
| 500 | 101.91 | 3.95 | 0 | 0.42 | 0.41 | 2.58 | 4.22 | 0.2 | 3.59 | 0.46 | 0.03 | 0.17 | 0.02 | 4.32 |
| 501 | 67.54 | 6.89 | 0.013 | 0.67 | 0.56 | 1.57 | 2.44 | 0.25 | 1.28 | 0.7 | 0.03 | 0.07 | 0.01 | 2.97 |
| 502 | 62.34 | 4.27 | 0.012 | 0.34 | 0.34 | 1.51 | 0.75 | 0.05 | 1.85 | 0.31 | 0.01 | 0.02 | 0.03 | 2.64 |
| 503 | 86.93 | 9.14 | 0.018 | 0.86 | 0.67 | 1.76 | 0.42 | 0.06 | 2.56 | 0.55 | 0.01 | 0 | 0.01 | 3.33 |
| 504 | 74.83 | 3.18 | 0 | 0.32 | 0.32 | 2.21 | 2.91 | 0.09 | 1.06 | 0.19 | 0.01 | 0.1 | 0.02 | 3.85 |
| 505 | 60.73 | 6.58 | 0.011 | 0.58 | 0.44 | 1.12 | 3.64 | 0.41 | 1.84 | 0.27 | 0.06 | 0.11 | 0.01 | 2.19 |
| 506 | 97.32 | 9.53 | 0.015 | 1.11 | 0.72 | 2.28 | 1.21 | 0.11 | 3.51 | 0.53 | 0.02 | 0 | 0.02 | 3.97 |
| 507 | 122.48 | 3.4 | 0.027 | 4.58 | 0.38 | 51.64 | 0.47 | 0.02 | 4.78 | 0.72 | 0.04 | 0.33 | 0.02 | 5.21 |
| 508 | 76.39 | 10.7 | 0.026 | 1.01 | 0.67 | 1.33 | 0.42 | 0.07 | 2.87 | 0.52 | 0.01 | 0.01 | 0.02 | 2.65 |
| 509 | 75.31 | 2.6 | 0 | 0.2 | 0.28 | 1.96 | 2.93 | 0.1 | 4.13 | 0.4 | 0.02 | 0.12 | 0.02 | 3.25 |
| 510 | 68.89 | 2.12 | 0 | 0.17 | 0.26 | 1.99 | 0.89 | 0.03 | 1.69 | 0.28 | 0 | 0.03 | 0.03 | 2.87 |
| 511 | 109.6 | 4.48 | 0 | 0.49 | 0.46 | 2.75 | 3.44 | 0.17 | 3.77 | 0.53 | 0.03 | 0.15 | 0.02 | 4.56 |
| 512 | 52.24 | 5.84 | 0.011 | 0.51 | 0.42 | 1.3 | 0.75 | 0.07 | 1.22 | 0.24 | 0.01 | 0.02 | 0.01 | 2.4 |
| 513 | 71.43 | 12.17 | 0.019 | 1.08 | 0.82 | 1.24 | 3.06 | 0.5 | 2.29 | 0.64 | 0.07 | 0.08 | 0.02 | 2.61 |
| 514 | 77.84 | 6.46 | 0.012 | 0.6 | 0.56 | 1.87 | 3.83 | 0.32 | 1.61 | 0.76 | 0.04 | 0.12 | 0.01 | 3.43 |
| 515 | 75.46 | 3.35 | 0 | 0.28 | 0.32 | 1.72 | 0.75 | 0.04 | 2.6 | 0.27 | 0.01 | 0.03 | 0.02 | 2.89 |

| | | | | | | | | | | | | | | |
|---|---|---|---|---|---|---|---|---|---|---|---|---|---|---|
| 516 | 89.16 | 3.79 | 0 | 0.36 | 0.36 | 2.22 | 2.18 | 0.1 | 1.85 | 0.43 | 0.02 | 0.05 | 0.01 | 3.51 |
| 517 | 93.84 | 9.76 | 0.023 | 0.96 | 0.77 | 2.43 | 3.91 | 0.37 | 2.1 | 0.59 | 0.05 | 0.12 | 0.02 | 4.39 |
| 518 | 80.51 | 5.77 | 0.017 | 0.49 | 0.55 | 1.99 | 6.16 | 0.5 | 2.41 | 0.38 | 0.07 | 0.25 | 0.03 | 4.47 |
| 519 | 64.97 | 6.15 | 0.015 | 0.46 | 0.45 | 1.39 | 1.67 | 0.19 | 1.87 | 0.42 | 0.02 | 0.06 | 0.03 | 2.44 |
| 520 | 85.39 | 1.79 | 0.024 | 2.78 | 0.26 | 47.9 | 0.34 | 0 | 2.63 | 0.59 | 0.02 | 0.29 | 0.05 | 4.98 |
| 521 | 58.88 | 1.91 | 0 | 0.15 | 0.24 | 1.54 | 1.91 | 0.07 | 2.36 | 0.29 | 0.01 | 0.08 | 0.02 | 2.23 |
| 522 | 84.54 | 10.7 | 0.021 | 0.99 | 0.76 | 1.54 | 3.4 | 0.44 | 2.33 | 0.49 | 0.07 | 0.11 | 0.02 | 3.03 |
| 523 | 92.81 | 3.82 | 0 | 0.37 | 0.34 | 2.64 | 3.65 | 0.13 | 2.16 | 0.44 | 0.02 | 0.1 | 0.03 | 4 |
| 524 | 101.25 | 2.88 | 0 | 0.27 | 0.33 | 2.47 | 4.91 | 0.16 | 3.93 | 0.58 | 0.02 | 0.2 | 0.05 | 3.91 |
| 525 | 61.39 | 9.2 | 0.023 | 0.98 | 0.6 | 1.29 | 0.34 | 0.06 | 2.08 | 0.41 | 0.01 | 0 | 0.02 | 2.52 |
| 526 | 0.72 | 0.12 | 0.09 | 17.19 | 0.29 | 114.56 | 0 | 0 | 5.7 | 0.84 | 0.31 | 4.73 | 0.06 | 1.64 |
| 527 | 69.76 | 3.61 | 0 | 0.33 | 0.38 | 1.7 | 3.14 | 0.16 | 2.53 | 0.44 | 0.03 | 0.11 | 0.02 | 3.22 |
| 528 | 56.26 | 4.22 | 0.01 | 0.42 | 0.34 | 1.26 | 1.42 | 0.13 | 1.01 | 0.22 | 0.02 | 0.03 | 0.01 | 2.04 |
| 529 | 73.02 | 5.25 | 0.01 | 0.48 | 0.47 | 1.78 | 2.11 | 0.15 | 1.24 | 0.65 | 0.02 | 0.07 | 0.01 | 3.26 |
| 530 | 87.62 | 8.48 | 0.014 | 0.82 | 0.78 | 1.9 | 6.55 | 0.78 | 1.55 | 0.5 | 0.11 | 0.24 | 0.01 | 4.26 |
| 531 | 88.42 | 8.32 | 0.015 | 0.67 | 0.64 | 1.9 | 3.49 | 0.33 | 1.62 | 0.47 | 0.04 | 0.07 | 0.01 | 3.59 |
| 532 | 83.36 | 12.03 | 0.017 | 1.38 | 0.9 | 1.65 | 0.55 | 0.09 | 4.03 | 0.8 | 0.01 | 0 | 0.01 | 3.38 |
| 533 | 85.9 | 3.56 | 0 | 0.32 | 0.34 | 2.62 | 5.17 | 0.19 | 2.98 | 0.26 | 0.03 | 0.21 | 0.02 | 4.64 |
| 534 | 88.76 | 3.06 | 0 | 0.3 | 0.38 | 2.48 | 7.26 | 0.28 | 3.5 | 0.38 | 0.05 | 0.33 | 0.02 | 5.05 |
| 535 | 92.04 | 3.52 | 0 | 0.36 | 0.4 | 2.77 | 5.02 | 0.17 | 4.26 | 0.59 | 0.03 | 0.2 | 0.01 | 4.36 |
| 536 | 63.81 | 1.61 | 0 | 0.13 | 0.23 | 1.59 | 1.13 | 0.03 | 1.16 | 0.18 | 0 | 0.05 | 0.01 | 2.58 |
| 537 | 73.19 | 1.47 | 0.014 | 2.21 | 0.24 | 37.4 | 0.26 | 0 | 1.66 | 0.49 | 0.02 | 0.24 | 0.03 | 3.85 |
| 538 | 58.03 | 2.35 | 0 | 0.22 | 0.27 | 1.65 | 2.95 | 0.1 | 2.06 | 0.29 | 0.01 | 0.09 | 0.01 | 2.53 |
| 539 | 0.33 | 0.1 | 0.079 | 16.51 | 0.28 | 113.27 | 0 | 0 | 5.01 | 0.77 | 0.3 | 5.11 | 0.07 | 1.69 |
| 540 | 89.98 | 3.62 | 0 | 0.36 | 0.34 | 2.44 | 3.5 | 0.14 | 1.45 | 0.42 | 0.02 | 0.14 | 0.02 | 3.64 |
| 541 | 84.7 | 7.89 | 0.015 | 0.88 | 0.69 | 2.04 | 5.42 | 0.5 | 3.63 | 0.57 | 0.07 | 0.18 | 0.01 | 4.25 |
| 542 | 71.04 | 8.09 | 0.013 | 0.79 | 0.55 | 1.43 | 0.37 | 0.05 | 2.63 | 0.43 | 0.01 | 0 | 0.01 | 2.59 |
| 543 | 63.85 | 4.88 | 0 | 0.39 | 0.4 | 1.6 | 3.63 | 0.29 | 2.81 | 0.49 | 0.03 | 0.13 | 0.01 | 2.92 |
| 544 | 120.4 | 6.78 | 0 | 0.92 | 0.69 | 3.6 | 5.62 | 0.27 | 7.4 | 0.76 | 0.05 | 0.19 | 0.01 | 5.74 |

| | | | | | | | | | | | | | | |
|---|---|---|---|---|---|---|---|---|---|---|---|---|---|---|
| 545 | 68.78 | 6.49 | 0.011 | 0.6 | 0.44 | 1.36 | 3.3 | 0.33 | 1.81 | 0.25 | 0.05 | 0.1 | 0.01 | 2.46 |
| 546 | 50.98 | 6.52 | 0.013 | 0.58 | 0.47 | 0.93 | 1.28 | 0.17 | 1.9 | 0.36 | 0.02 | 0.03 | 0.01 | 1.85 |
| 547 | 102.54 | 2.7 | 0.02 | 3.74 | 0.3 | 42.39 | 0.47 | 0.01 | 4.87 | 0.64 | 0.03 | 0.28 | 0.02 | 5.11 |
| 548 | 79.48 | 5.71 | 0.012 | 0.5 | 0.44 | 1.82 | 0.41 | 0.04 | 2.15 | 0.38 | 0.01 | 0 | 0.01 | 3.25 |
| 549 | 80.44 | 3.14 | 0 | 0.31 | 0.31 | 2.25 | 1.76 | 0.07 | 2.14 | 0.29 | 0.01 | 0.07 | 0.01 | 3.16 |
| 550 | 87.29 | 6.52 | 0.013 | 0.83 | 0.59 | 3.31 | 2.81 | 0.2 | 4.16 | 0.8 | 0.03 | 0.13 | 0.02 | 4.18 |
| 551 | 59.31 | 6.37 | 0.011 | 0.58 | 0.52 | 1.23 | 3.25 | 0.34 | 1.22 | 0.47 | 0.05 | 0.09 | 0.01 | 2.81 |
| 552 | 96.47 | 3 | 0 | 0.31 | 0.31 | 2.77 | 1.52 | 0.04 | 2.23 | 0.28 | 0.01 | 0.05 | 0.01 | 4.58 |
| 553 | 75.11 | 3.08 | 0 | 0.28 | 0.33 | 1.83 | 3.74 | 0.15 | 3.47 | 0.45 | 0.02 | 0.14 | 0.01 | 3.2 |
| 554 | 102.46 | 2.69 | 0.02 | 3.67 | 0.31 | 42.43 | 0.42 | 0.01 | 4.28 | 0.62 | 0.03 | 0.32 | 0.02 | 4.94 |
| 555 | 54 | 6.83 | 0 | 0.43 | 0.38 | 1.15 | 0.7 | 0.53 | 1.31 | 0.24 | 0.08 | 0.18 | 0.01 | 2.18 |
| 556 | 69.54 | 6.59 | 0 | 0.71 | 0.59 | 1.66 | 4.83 | 0.42 | 1.58 | 0.82 | 0.06 | 0.14 | 0.01 | 3.33 |
| 557 | 73.07 | 5.07 | 0 | 0.66 | 0.54 | 1.53 | 4.98 | 0.07 | 2.96 | 0.49 | 0.01 | 0.02 | 0.01 | 2.97 |
| 558 | 48.47 | 5.49 | 0.012 | 0.54 | 0.42 | 1.01 | 1.1 | 0.13 | 2.12 | 0.35 | 0.02 | 0.02 | 0.01 | 1.81 |
| 559 | 72.5 | 6.25 | 0.014 | 0.66 | 0.77 | 1.83 | 7.38 | 0.68 | 0.98 | 0.33 | 0.08 | 0.22 | 0.01 | 5.24 |
| 560 | 64.79 | 4.45 | 0 | 0.38 | 0.41 | 1.33 | 2.08 | 0.17 | 0.88 | 0.54 | 0.02 | 0.08 | 0.01 | 2.63 |
| 561 | 88.06 | 2.64 | 0 | 0.24 | 0.31 | 2.4 | 4.92 | 0.15 | 3.17 | 0.48 | 0.02 | 0.19 | 0.01 | 4.02 |
| 562 | 121.15 | 5.47 | 0 | 0.55 | 0.48 | 2.88 | 4.38 | 0.21 | 4.05 | 0.48 | 0.03 | 0.19 | 0.02 | 4.8 |
| 563 | 62.49 | 2.66 | 0 | 0.25 | 0.29 | 1.73 | 2.26 | 0.08 | 2.12 | 0.36 | 0.01 | 0.06 | 0.01 | 2.79 |
| 564 | 77.73 | 6.9 | 0.014 | 0.6 | 0.59 | 1.55 | 5 | 0.49 | 1.17 | 0.38 | 0.07 | 0.18 | 0.02 | 3.66 |
| 565 | 66.13 | 5.68 | 0.011 | 0.53 | 0.52 | 1.53 | 3.36 | 0.28 | 1.4 | 0.45 | 0.04 | 0.1 | 0.01 | 3.29 |
| 566 | 82.91 | 8.5 | 0.016 | 0.97 | 0.74 | 2.02 | 5.3 | 0.51 | 4.71 | 1.09 | 0.07 | 0.15 | 0.02 | 3.75 |
| 567 | 65.34 | 5.11 | 0.01 | 0.51 | 0.44 | 1.45 | 2.47 | 0.19 | 1.13 | 0.22 | 0.03 | 0.02 | 0.01 | 3 |
| 568 | 56.24 | 4.28 | 0 | 0.38 | 0.32 | 1.25 | 2.99 | 0.25 | 1.45 | 0.21 | 0.03 | 0.09 | 0.01 | 2.13 |
| 569 | 78.54 | 7.44 | 0.015 | 0.79 | 0.58 | 1.96 | 3.36 | 0.29 | 2.68 | 0.45 | 0.04 | 0.1 | 0.01 | 3.49 |
| 570 | 0.6 | 0.11 | 0.092 | 17.84 | 0.31 | 117.11 | 0 | 0 | 5.42 | 0.86 | 0.33 | 5.27 | 0.06 | 1.73 |
| 571 | 83.17 | 5.86 | 0.012 | 0.64 | 0.51 | 2.15 | 0.53 | 0.04 | 2.06 | 0.41 | 0.01 | 0 | 0.02 | 4.01 |
| 572 | 94.58 | 2.15 | 0.018 | 3.07 | 0.3 | 43.87 | 0.15 | 0 | 1.89 | 0.6 | 0.01 | 0.06 | 0.03 | 4.8 |
| 573 | 97.92 | 7.65 | 0.017 | 0.81 | 0.56 | 2.37 | 0.68 | 0.06 | 3.44 | 0.45 | 0.01 | 0 | 0.02 | 3.94 |

| | | | | | | | | | | | | | | |
|---|---|---|---|---|---|---|---|---|---|---|---|---|---|---|
| 574 | 56.23 | 1.94 | 0 | 0.2 | 0.25 | 1.78 | 1.74 | 0.05 | 0.99 | 0.2 | 0.01 | 0.06 | 0.01 | 2.46 |
| 575 | 87.12 | 7 | 0.013 | 0.63 | 0.53 | 1.9 | 4.51 | 0.37 | 1.13 | 0.38 | 0.05 | 0.16 | 0.02 | 3.52 |
| 576 | 68.01 | 2.78 | 0 | 0.23 | 0.31 | 1.83 | 4.42 | 0.17 | 1.41 | 0.2 | 0.02 | 0.17 | 0.01 | 3.78 |
| 577 | 77.24 | 2.92 | 0 | 0.27 | 0.3 | 2.25 | 0.89 | 0.02 | 3.49 | 0.23 | 0 | 0.03 | 0.01 | 3.72 |
| 578 | 84.21 | 8.24 | 0.016 | 0.84 | 0.71 | 1.79 | 5.64 | 0.59 | 2.18 | 0.43 | 0.08 | 0.18 | 0.01 | 4.21 |
| 579 | 53.01 | 2.7 | 0 | 0.23 | 0.28 | 1.22 | 2.65 | 0.13 | 1 | 0.26 | 0.02 | 0.1 | 0.01 | 2.2 |
| 580 | 50.12 | 2.11 | 0 | 0.18 | 0.25 | 1.13 | 0.67 | 0.03 | 0.93 | 0.18 | 0 | 0.02 | 0.01 | 1.92 |
| 581 | 67.04 | 3.29 | 0 | 0.32 | 0.31 | 1.9 | 1.86 | 0.07 | 1.75 | 0.27 | 0.01 | 0.05 | 0.01 | 2.92 |
| 582 | 76.31 | 4.56 | 0 | 0.47 | 0.58 | 1.66 | 6.2 | 0.46 | 0.8 | 0.27 | 0.08 | 0.25 | 0.01 | 4.79 |
| 583 | 87.51 | 4.87 | 0 | 0.53 | 0.43 | 2.23 | 0.97 | 0.05 | 5.13 | 0.43 | 0.01 | 0.04 | 0.01 | 3.67 |
| 584 | 53.11 | 2.77 | 0 | 0.22 | 0.28 | 1.19 | 0.56 | 0.03 | 1.62 | 0.22 | 0 | 0 | 0.01 | 2.14 |
| 585 | 59.32 | 3.12 | 0 | 0.29 | 0.31 | 1.61 | 2.09 | 0.08 | 2.67 | 0.37 | 0.01 | 0.07 | 0.01 | 2.63 |
| 586 | 67.88 | 2.48 | 0 | 0.21 | 0.29 | 1.75 | 2.9 | 0.11 | 3.48 | 0.39 | 0.02 | 0.13 | 0.01 | 2.81 |
| 587 | 90.77 | 3.36 | 0.021 | 4.44 | 0.34 | 35.04 | 0.4 | 0.01 | 3.4 | 0.55 | 0.03 | 0.25 | 0.02 | 4.62 |
| 588 | 70.52 | 6.06 | 0.013 | 0.67 | 0.46 | 2.21 | 2.21 | 0.2 | 1.63 | 0.52 | 0.03 | 0.11 | 0.01 | 2.89 |
| 589 | 67.26 | 2.62 | 0 | 0.23 | 0.31 | 1.6 | 2.01 | 0.09 | 0.82 | 0.18 | 0.01 | 0.08 | 0.01 | 2.92 |
| 590 | 116.9 | 2.17 | 0.015 | 3.23 | 0.33 | 55.71 | 0.19 | 0 | 2.83 | 0.78 | 0.01 | 0.18 | 0.03 | 5.91 |
| 591 | 86.08 | 2.9 | 0 | 0.29 | 0.35 | 2.04 | 4.14 | 0.16 | 3.03 | 0.67 | 0.02 | 0.17 | 0.01 | 3.52 |
| 592 | 79.7 | 7.73 | 0.011 | 0.75 | 0.7 | 1.74 | 6.8 | 0.74 | 1.3 | 0.46 | 0.1 | 0.24 | 0.01 | 4.02 |
| 593 | 79.37 | 3.44 | 0 | 0.34 | 0.35 | 2.45 | 5.07 | 0.15 | 3.65 | 0.53 | 0.02 | 0.17 | 0.01 | 3.91 |
| 594 | 79.48 | 3.16 | 0 | 0.3 | 0.34 | 2.07 | 4.33 | 0.16 | 1.47 | 0.71 | 0.02 | 0.15 | 0.01 | 3.67 |
| 595 | 0.26 | 0.1 | 0.087 | 14.07 | 0.25 | 89.01 | 0 | 0 | 4.39 | 0.59 | 0.24 | 4 | 0.07 | 1.14 |
| 596 | 79.11 | 5.63 | 0.013 | 0.49 | 0.43 | 1.78 | 3.22 | 0.24 | 1.17 | 0.42 | 0.03 | 0.12 | 0.02 | 3.11 |
| 597 | 73.02 | 3.8 | 0 | 0.36 | 0.34 | 2.08 | 2.9 | 0.11 | 1.85 | 0.4 | 0.02 | 0.1 | 0.01 | 3.44 |
| 598 | 66.2 | 2.55 | 0 | 0.21 | 0.29 | 1.64 | 5.14 | 0.22 | 1.99 | 0.29 | 0.03 | 0.21 | 0.01 | 2.87 |
| 599 | 82.34 | 4.2 | 0 | 0.41 | 0.4 | 1.96 | 3.69 | 0.2 | 1.9 | 0.3 | 0.03 | 0.15 | 0.01 | 3.39 |
| 600 | 79.55 | 8.78 | 0.015 | 1.04 | 0.8 | 1.94 | 6.05 | 0.72 | 2.51 | 0.52 | 0.08 | 0.16 | 0.01 | 4.33 |
| 601 | 94.92 | 4.04 | 0 | 0.4 | 0.43 | 2.31 | 4.41 | 0.21 | 2.52 | 0.42 | 0.03 | 0.17 | 0.01 | 4.54 |
| 602 | 53.86 | 2.35 | 0 | 0.2 | 0.27 | 1.46 | 0.73 | 0.03 | 1.23 | 0.26 | 0 | 0.03 | 0.01 | 2.51 |

| | | | | | | | | | | | | | | |
|---|---|---|---|---|---|---|---|---|---|---|---|---|---|---|
| 603 | 78.83 | 3.15 | 0 | 0.27 | 0.32 | 2.17 | 3.87 | 0.17 | 1.78 | 0.21 | 0.02 | 0.17 | 0.01 | 3.91 |
| 604 | 80.19 | 3.29 | 0 | 0.33 | 0.34 | 2.26 | 5.27 | 0.19 | 3.49 | 0.52 | 0.03 | 0.18 | 0.01 | 4.1 |
| 605 | 1.91 | 0.3 | 0.255 | 18.68 | 0.38 | 47.14 | 0 | 0 | 3.23 | 0.43 | 0.34 | 1.79 | 0.05 | 1.11 |
| 606 | 60.17 | 7.32 | 0.01 | 0.58 | 0.51 | 1.58 | 1.19 | 0.14 | 2.02 | 0.47 | 0.02 | 0.05 | 0.01 | 2.45 |
| 607 | 32.13 | 2.87 | 0 | 0.25 | 0.19 | 0.97 | 0.86 | 0.07 | 0.3 | 0.05 | 0.01 | 0.03 | 0 | 1.22 |
| 608 | 81.08 | 4.36 | 0.07 | 5.8 | 0.35 | 37.89 | 0.37 | 0.03 | 1.44 | 0.4 | 0.05 | 0.3 | 0.06 | 3.95 |
| 609 | 62.12 | 5.63 | 0 | 0.49 | 0.4 | 1.74 | 1.79 | 0.16 | 0.55 | 0.25 | 0.02 | 0.07 | 0 | 2.49 |
| 610 | 72.91 | 5.07 | 0 | 0.34 | 0.35 | 2.24 | 0.65 | 0.05 | 1.76 | 0.15 | 0 | 0.04 | 0.01 | 3.05 |
| 611 | 79.8 | 3.91 | 0.09 | 5.35 | 0.38 | 36.8 | 0.12 | 0.01 | 0.81 | 0.45 | 0.02 | 0.1 | 0.08 | 4.33 |
| 612 | 0.31 | 0.01 | 0.05 | 4.32 | 0.14 | 146.21 | 0.01 | 0 | 4.53 | 2.26 | 0.02 | 0.62 | 0.24 | 2.08 |
| 613 | 77.07 | 2.77 | 0.07 | 4.6 | 0.29 | 47.63 | 0.13 | 0.01 | 1.13 | 0.55 | 0.01 | 0.15 | 0.09 | 4.22 |
| 614 | 68.59 | 2.47 | 0.07 | 4.17 | 0.24 | 48.1 | 0.28 | 0.02 | 1.1 | 0.48 | 0.03 | 0.33 | 0.11 | 3.98 |
| 615 | 0.33 | 0.02 | 0.12 | 6.57 | 0.19 | 141.85 | 0.01 | 0 | 5.46 | 0.45 | 0.04 | 0.68 | 0.22 | 1.56 |
| 616 | 58.26 | 5.21 | 0.01 | 0.43 | 0.43 | 1.69 | 1.43 | 0.15 | 0.63 | 0.47 | 0.02 | 0.06 | 0 | 2.47 |
| 617 | 78.76 | 4.29 | 0.08 | 6.12 | 0.39 | 36.6 | 0.38 | 0.03 | 1.25 | 0.44 | 0.05 | 0.31 | 0.06 | 3.77 |
| 618 | 61.3 | 5.27 | 0.01 | 0.4 | 0.36 | 1.68 | 2.08 | 0.17 | 0.46 | 0.22 | 0.02 | 0.09 | 0.02 | 2.59 |
| 619 | 60.58 | 3.55 | 0 | 0.31 | 0.42 | 1.26 | 1.11 | 0.18 | 0.72 | 0.23 | 0.02 | 0.07 | 0 | 1.72 |
| 620 | 0.36 | 0.02 | 0.11 | 6.26 | 0.22 | 136.93 | 0.01 | 0 | 2.62 | 0.51 | 0.04 | 0.58 | 0.2 | 2.05 |
| 621 | 60.89 | 5.76 | 0.01 | 0.61 | 0.47 | 2.3 | 2.97 | 0.29 | 0.96 | 0.32 | 0.04 | 0.13 | 0.02 | 2.82 |
| 622 | 0.36 | 0.02 | 0.15 | 6.82 | 0.18 | 134.98 | 0.01 | 0 | 4.37 | 0.43 | 0.05 | 0.68 | 0.21 | 1.43 |
| 623 | 61.46 | 8.85 | 0.01 | 0.61 | 0.56 | 1.58 | 1.56 | 0.2 | 1.51 | 0.46 | 0.02 | 0.06 | 0.01 | 2.45 |
| 624 | 0.39 | 0.02 | 0.13 | 6.41 | 0.17 | 136.58 | 0.01 | 0 | 3.67 | 0.41 | 0.04 | 0.67 | 0.22 | 1.52 |
| 625 | 63.94 | 5.6 | 0 | 0.49 | 0.4 | 1.91 | 1.94 | 0.17 | 0.49 | 0.25 | 0.02 | 0.08 | 0.01 | 2.7 |
| 626 | 44.27 | 3.6 | 0 | 0.31 | 0.24 | 1.41 | 1.33 | 0.1 | 0.36 | 0.08 | 0.01 | 0.05 | 0 | 1.82 |
| 627 | 74.54 | 3.29 | 0.09 | 5.53 | 0.33 | 45.16 | 0.11 | 0.01 | 0.9 | 0.46 | 0.02 | 0.14 | 0.08 | 4 |
| 628 | 70.87 | 7.67 | 0.01 | 0.64 | 0.62 | 2.04 | 7.64 | 1000 | 1.62 | 0.35 | 0.11 | 0.3 | 0.02 | 3.69 |
| 629 | 70.13 | 3.59 | 0.09 | 5.36 | 0.35 | 33.21 | 0.1 | 0.01 | 0.75 | 0.41 | 0.02 | 0.09 | 0.07 | 3.85 |
| 630 | 52.07 | 5.46 | 0 | 0.54 | 0.44 | 1.35 | 0.34 | 0.04 | 0.3 | 0.14 | 0 | 0.01 | 0 | 2.33 |
| 631 | 62.05 | 7.65 | 0.01 | 0.55 | 0.5 | 1.72 | 1.49 | 0.17 | 1.46 | 0.45 | 0.02 | 0.06 | 0.01 | 2.54 |

| | | | | | | | | | | | | | | |
|---|---|---|---|---|---|---|---|---|---|---|---|---|---|---|
| 632 | 80.27 | 5.21 | 0.01 | 0.45 | 0.48 | 2.41 | 7.15 | 0.47 | 1.61 | 0.33 | 0.07 | 0.31 | 0.02 | 4.48 |
| 633 | 41.81 | 3.53 | 0 | 0.33 | 0.26 | 1.33 | 1.25 | 0.12 | 0.39 | 0.13 | 0.02 | 0.07 | 0 | 1.58 |
| 634 | 75.55 | 9.19 | 0.01 | 0.74 | 0.66 | 2.07 | 3.63 | 0.43 | 1.36 | 0.75 | 0.06 | 0.14 | 0 | 3.4 |
| 635 | 76.94 | 3.31 | 0.09 | 4.45 | 0.27 | 36.94 | 0.36 | 0.02 | 1.17 | 0.36 | 0.04 | 0.3 | 0.11 | 3.72 |
| 636 | 51.6 | 5.76 | 0.01 | 0.5 | 0.45 | 1.48 | 1.72 | 0.2 | 1.17 | 0.22 | 0.02 | 0.07 | 0 | 2.12 |
| 637 | 71.53 | 3.6 | 0.08 | 4.85 | 0.34 | 32.34 | 0.11 | 0.01 | 0.79 | 0.37 | 0.01 | 0.09 | 0.07 | 3.87 |
| 638 | 1.15 | 0.08 | 0.1 | 7.91 | 0.18 | 128.41 | 0.05 | 0.01 | 4.56 | 0.93 | 0.42 | 6.01 | 0.11 | 1.52 |
| 639 | 49.78 | 4.93 | 0 | 0.37 | 0.35 | 1.43 | 1.39 | 0.13 | 0.52 | 0.4 | 0.02 | 0.06 | 0 | 2.1 |
| 640 | 0.79 | 0.04 | 0.06 | 4.19 | 0.16 | 164.48 | 0.02 | 0 | 4.63 | 2.3 | 0.02 | 0.65 | 0.28 | 2.33 |
| 641 | 65.31 | 6.42 | 0.01 | 0.64 | 0.52 | 2.27 | 2.63 | 0.27 | 1.62 | 0.38 | 0.04 | 0.13 | 0.01 | 2.94 |
| 642 | 0.37 | 0.02 | 0.05 | 5.11 | 0.18 | 161.79 | 0.01 | 0 | 4.17 | 2.56 | 0.03 | 0.64 | 0.24 | 2.3 |
| 643 | 68.91 | 9.21 | 0.01 | 0.73 | 0.67 | 1.85 | 3.39 | 0.45 | 1.15 | 0.63 | 0.06 | 0.13 | 0 | 3.05 |
| 644 | 47.89 | 4.69 | 0 | 0.34 | 0.31 | 1.42 | 1.3 | 0.12 | 0.54 | 0.35 | 0.01 | 0.05 | 0 | 2.01 |
| 645 | 0.36 | 0.02 | 0.11 | 6.69 | 0.2 | 135.85 | 0.01 | 0 | 2.86 | 0.51 | 0.04 | 0.58 | 0.19 | 1.96 |
| 646 | 56.54 | 6.34 | 0.01 | 0.52 | 0.49 | 1.57 | 2.7 | 0.32 | 0.77 | 0.3 | 0.04 | 0.11 | 0.01 | 2.53 |
| 647 | 68.02 | 2.48 | 0.07 | 4.4 | 0.26 | 48.38 | 0.29 | 0.02 | 1.08 | 0.51 | 0.03 | 0.34 | 0.1 | 3.9 |
| 648 | 51.97 | 4.43 | 0 | 0.45 | 0.37 | 1.48 | 0.3 | 0.03 | 0.46 | 0.16 | 0 | 0.01 | 0 | 2.38 |
| 649 | 39.48 | 3.63 | 0 | 0.29 | 0.24 | 1.17 | 1.1 | 0.1 | 0.36 | 0.07 | 0.01 | 0.05 | 0 | 1.52 |
| 650 | 77.26 | 7.37 | 0.01 | 0.59 | 0.62 | 2.21 | 7.02 | 0.62 | 1.29 | 0.32 | 0.09 | 0.28 | 0.02 | 4.18 |
| 651 | 58.62 | 4.74 | 0 | 0.33 | 0.33 | 1.85 | 0.53 | 0.05 | 1.38 | 0.13 | 0 | 0.03 | 0 | 2.45 |
| 652 | 82.46 | 4.08 | 0.07 | 5.84 | 0.34 | 37.98 | 0.36 | 0.03 | 1.32 | 0.4 | 0.05 | 0.3 | 0.07 | 3.96 |
| 653 | 61.56 | 6.12 | 0.01 | 0.68 | 0.44 | 3.06 | 1.98 | 0.19 | 1.42 | 0.27 | 0.03 | 0.08 | 0 | 2.48 |
| 654 | 58.27 | 5.04 | 0 | 0.46 | 0.37 | 2.44 | 1.4 | 0.13 | 0.72 | 0.44 | 0.02 | 0.09 | 0.01 | 2.38 |
| 655 | 58.9 | 2.96 | 0 | 0.26 | 0.39 | 0.77 | 1.25 | 0.18 | 0.26 | 0.21 | 0.02 | 0.07 | 0 | 1.23 |
| 656 | 55.44 | 6.15 | 0.01 | 0.48 | 0.45 | 1.46 | 2.63 | 0.3 | 0.72 | 0.28 | 0.04 | 0.1 | 0.02 | 2.45 |
| 657 | 72.64 | 2.11 | 0.06 | 0.38 | 0.22 | 51.93 | 0.29 | 0.02 | 1.16 | 0.48 | 0.03 | 0.34 | 0.13 | 4.06 |
| 658 | 0.45 | 0.03 | 0.1 | 9.02 | 0.19 | 143.23 | 0.04 | 0 | 4.95 | 1.1 | 0.5 | 6.66 | 0.1 | 1.7 |
| 659 | 69.68 | 6.07 | 0 | 0.43 | 0.41 | 2.26 | 0.66 | 0.07 | 1.34 | 0.15 | 0 | 0.04 | 0.01 | 3.04 |
| 660 | 75.69 | 3.96 | 0.1 | 5.73 | 0.38 | 36.76 | 0.11 | 0.01 | 0.77 | 0.4 | 0.02 | 0.1 | 0.07 | 3.97 |

| | | | | | | | | | | | | | | |
|---|---|---|---|---|---|---|---|---|---|---|---|---|---|---|
| 661 | 62.92 | 7.69 | 0.01 | 0.59 | 0.53 | 1.76 | 2.79 | 0.32 | 1.04 | 0.55 | 0.04 | 0.11 | 0 | 2.71 |
| 662 | 0.7 | 0.04 | 0.05 | 4.54 | 0.18 | 151.74 | 0.01 | 0 | 4.19 | 2.26 | 0.02 | 0.61 | 0.25 | 2.32 |
| 663 | 51.73 | 4.42 | 0 | 0.4 | 0.35 | 1.38 | 0.3 | 0.03 | 0.32 | 0.14 | 0 | 0.01 | 0 | 2.36 |
| 664 | 72.86 | 2.31 | 0.06 | 3.78 | 0.22 | 44.96 | 0.11 | 0.01 | 1.08 | 0.47 | 0.01 | 0.13 | 0.11 | 3.85 |
| 665 | 74.82 | 6.9 | 0.01 | 0.58 | 0.64 | 2.12 | 6.22 | 0.56 | 1.13 | 0.29 | 0.08 | 0.25 | 0.02 | 4.18 |
| 666 | 72.88 | 2.48 | 0.07 | 4.45 | 0.27 | 51.01 | 0.31 | 0.02 | 1.18 | 0.47 | 0.03 | 0.36 | 0.11 | 4.1 |
| 667 | 0.47 | 0.03 | 0.08 | 8.14 | 0.18 | 126.59 | 0.03 | 0 | 4.6 | 0.93 | 0.44 | 6.2 | 0.1 | 1.57 |
| 668 | 50.04 | 5.39 | 0 | 0.48 | 0.39 | 1.27 | 0.29 | 0.04 | 0.32 | 0.12 | 0 | 0.01 | 0 | 2.16 |
| 669 | 58.57 | 6.04 | 0.01 | 0.55 | 0.44 | 1.79 | 1.57 | 0.18 | 1.28 | 0.24 | 0.02 | 0.06 | 0 | 2.38 |
| 670 | 77.61 | 2.61 | 0.07 | 4.44 | 0.25 | 48.61 | 0.12 | 0.01 | 1.24 | 0.55 | 0.01 | 0.15 | 0.1 | 4.08 |
| 671 | 72.05 | 9.64 | 0.01 | 0.71 | 0.68 | 1.92 | 3.66 | 0.44 | 1.24 | 0.65 | 0.06 | 0.14 | 0.01 | 2.99 |
| 672 | 0.6 | 0.06 | 0.1 | 8.18 | 0.18 | 135.35 | 0.04 | 0 | 4.43 | 0.99 | 0.44 | 6.42 | 0.11 | 1.61 |
| 673 | 0.33 | 0.02 | 0.14 | 7.29 | 0.18 | 128.49 | 0.01 | 0 | 2.97 | 0.49 | 0.05 | 0.57 | 0.19 | 1.81 |
| 674 | 0.49 | 0.03 | 0.13 | 6.38 | 0.17 | 136.28 | 0.01 | 0 | 5.17 | 0.4 | 0.04 | 0.66 | 0.2 | 1.46 |
| 675 | 0.36 | 0.02 | 0.12 | 6.49 | 0.2 | 130.39 | 0.01 | 0 | 2.8 | 0.47 | 0.04 | 0.57 | 0.19 | 1.83 |
| 676 | 62.67 | 7.86 | 0.01 | 0.55 | 0.52 | 1.71 | 1.36 | 0.16 | 1.71 | 0.45 | 0.02 | 0.06 | 0.01 | 2.55 |
| 677 | 72.94 | 3.85 | 0 | 0.31 | 0.3 | 2.19 | 0.6 | 0.04 | 2.56 | 0.18 | 0 | 0.03 | 0 | 2.98 |
| 678 | 0.21 | 0.02 | 0.2 | 7.3 | 1.32 | 76.28 | 0 | 0 | 1.55 | 0.27 | 0.05 | 0.33 | 0.17 | 6 |
| 679 | 0.47 | 0.18 | 0.29 | 5.97 | 0.42 | 42.88 | 0 | 0 | 0.55 | 0.42 | 0.03 | 0.22 | 0.14 | 2.56 |
| 680 | 0.26 | 0.1 | 0.49 | 11.63 | 1.78 | 65.05 | 0 | 0 | 0.85 | 0.22 | 0.08 | 0.27 | 0.17 | 3.8 |
| 681 | 24.45 | 9.57 | 0.39 | 8.11 | 0.74 | 8.43 | 0.04 | 0.02 | 0.23 | 0.16 | 0.03 | 0.03 | 0.04 | 1.41 |
| 682 | 0.17 | 0.02 | 0.34 | 9.36 | 0.54 | 66.33 | 0 | 0 | 0.96 | 0.19 | 0.07 | 0.37 | 0.18 | 2.17 |
| 683 | 0.18 | 0.03 | 0.94 | 10.26 | 0.62 | 67.86 | 0 | 0 | 1.29 | 0.63 | 0.07 | 0.31 | 0.22 | 2.89 |
| 684 | 48.54 | 3.21 | 0.00 | 0.34 | 0.27 | 1.67 | 1.71 | 0.12 | 0.69 | 0.18 | 0.02 | 0.08 | 0.01 | 1.96 |
| 685 | 70.89 | 4.15 | 0.00 | 0.27 | 0.21 | 2.32 | 0.38 | 0.02 | 0.83 | 0.21 | 0.00 | 0.02 | 0.01 | 2.86 |
| 686 | 65.98 | 2.27 | 0.04 | 4.88 | 0.27 | 47.28 | 0.24 | 0.01 | 1.85 | 0.38 | 0.03 | 0.28 | 0.03 | 3.45 |
| 687 | 66.33 | 4.70 | 0.00 | 0.50 | 0.38 | 2.12 | 3.44 | 0.23 | 2.12 | 0.28 | 0.04 | 0.14 | 0.02 | 2.80 |
| 688 | 0.37 | 0.02 | 0.08 | 7.53 | 0.19 | 133.22 | 0.01 | 0.00 | 3.43 | 0.42 | 0.04 | 0.41 | 0.06 | 2.12 |
| 689 | 63.74 | 2.40 | 0.05 | 5.07 | 0.28 | 45.70 | 0.22 | 0.01 | 1.46 | 0.36 | 0.04 | 0.26 | 0.03 | 3.37 |

| | | | | | | | | | | | | | | |
|---|---|---|---|---|---|---|---|---|---|---|---|---|---|---|
| 690 | 67.64 | 4.36 | 0.00 | 0.46 | 0.35 | 2.13 | 1.69 | 0.10 | 4.11 | 0.47 | 0.02 | 0.07 | 0.02 | 2.75 |
| 691 | 49.94 | 3.00 | 0.00 | 0.42 | 0.23 | 2.75 | 1.31 | 0.07 | 2.82 | 0.22 | 0.01 | 0.06 | 0.02 | 2.01 |
| 692 | 51.38 | 3.67 | 0.00 | 0.35 | 0.29 | 1.63 | 1.93 | 0.12 | 2.43 | 0.28 | 0.02 | 0.08 | 0.02 | 2.13 |
| 693 | 57.10 | 2.93 | 0.00 | 0.31 | 0.24 | 1.86 | 0.41 | 0.02 | 2.11 | 0.20 | 0.00 | 0.02 | 0.02 | 2.25 |
| 694 | 48.50 | 3.19 | 0.00 | 0.32 | 0.26 | 1.58 | 2.08 | 0.13 | 0.88 | 0.17 | 0.02 | 0.08 | 0.01 | 1.93 |
| 695 | 74.79 | 3.91 | 0.00 | 0.34 | 0.32 | 2.53 | 3.82 | 0.18 | 4.42 | 0.48 | 0.03 | 0.16 | 0.02 | 3.27 |
| 696 | 43.09 | 2.71 | 0.00 | 0.26 | 0.22 | 1.40 | 0.23 | 0.02 | 0.95 | 0.11 | 0.00 | 0.01 | 0.01 | 1.68 |
| 697 | 107.13 | 5.12 | 0.00 | 0.56 | 0.41 | 3.34 | 2.42 | 0.11 | 2.91 | 0.35 | 0.02 | 0.11 | 0.02 | 4.29 |
| 698 | 74.14 | 4.99 | 0.00 | 0.40 | 0.34 | 2.43 | 0.44 | 0.04 | 1.00 | 0.30 | 0.00 | 0.02 | 0.02 | 3.01 |
| 699 | 60.44 | 3.61 | 0.00 | 0.33 | 0.29 | 1.99 | 2.29 | 0.12 | 4.12 | 0.38 | 0.02 | 0.09 | 0.01 | 2.51 |
| 700 | 41.98 | 2.66 | 0.00 | 0.25 | 0.19 | 1.36 | 1.39 | 0.07 | 0.59 | 0.12 | 0.01 | 0.06 | 0.01 | 1.65 |
| 701 | 89.96 | 4.53 | 0.00 | 0.41 | 0.31 | 3.03 | 1.82 | 0.08 | 2.36 | 0.27 | 0.01 | 0.07 | 0.01 | 3.63 |
| 702 | 46.24 | 2.50 | 0.00 | 0.25 | 0.20 | 1.44 | 1.20 | 0.06 | 2.64 | 0.26 | 0.01 | 0.05 | 0.02 | 1.82 |
| 703 | 51.05 | 4.04 | 0.00 | 0.37 | 0.30 | 1.75 | 1.07 | 0.08 | 1.12 | 0.23 | 0.01 | 0.05 | 0.01 | 2.35 |
| 704 | 0.60 | 0.04 | 0.09 | 7.07 | 0.24 | 120.42 | 0.02 | 0.00 | 2.57 | 0.41 | 0.04 | 0.46 | 0.07 | 2.09 |
| 705 | 68.68 | 6.08 | 0.00 | 0.61 | 0.48 | 2.10 | 1.45 | 0.12 | 4.72 | 0.61 | 0.02 | 0.06 | 0.01 | 2.91 |
| 706 | 58.30 | 4.00 | 0.00 | 0.39 | 0.31 | 1.82 | 2.85 | 0.17 | 2.44 | 0.26 | 0.03 | 0.11 | 0.02 | 2.44 |
| 707 | 57.97 | 3.74 | 0.00 | 0.35 | 0.30 | 1.84 | 1.69 | 0.10 | 1.91 | 0.20 | 0.02 | 0.07 | 0.02 | 2.39 |
| 708 | 32.37 | 2.28 | 0.00 | 0.23 | 0.18 | 1.29 | 0.19 | 0.02 | 0.76 | 0.08 | 0.00 | 0.01 | 0.01 | 1.29 |
| 709 | 56.20 | 4.06 | 0.00 | 0.40 | 0.34 | 1.76 | 2.08 | 0.14 | 3.00 | 0.36 | 0.02 | 0.08 | 0.01 | 2.39 |
| 710 | 60.95 | 3.45 | 0.00 | 0.34 | 0.27 | 2.04 | 1.84 | 0.10 | 3.38 | 0.25 | 0.02 | 0.08 | 0.01 | 2.53 |
| 711 | 59.09 | 3.86 | 0.00 | 0.40 | 0.32 | 1.89 | 3.29 | 0.20 | 2.16 | 0.25 | 0.03 | 0.13 | 0.01 | 2.56 |
| 712 | 40.62 | 3.28 | 0.00 | 0.30 | 0.26 | 1.29 | 0.27 | 0.03 | 0.57 | 0.09 | 0.00 | 0.01 | 0.00 | 1.65 |
| 713 | 39.95 | 3.41 | 0.00 | 0.33 | 0.26 | 1.39 | 1.44 | 0.11 | 0.58 | 0.14 | 0.02 | 0.06 | 0.00 | 1.64 |
| 714 | 53.86 | 3.83 | 0.00 | 0.35 | 0.31 | 1.77 | 1.99 | 0.13 | 2.31 | 0.28 | 0.02 | 0.08 | 0.02 | 2.24 |
| 715 | 0.39 | 0.02 | 0.10 | 7.26 | 0.25 | 121.25 | 0.01 | 0.00 | 2.08 | 0.41 | 0.04 | 0.46 | 0.07 | 2.17 |
| 716 | 41.05 | 2.59 | 0.00 | 0.27 | 0.21 | 1.50 | 0.93 | 0.05 | 1.12 | 0.18 | 0.01 | 0.04 | 0.01 | 1.70 |
| 717 | 70.99 | 2.25 | 0.05 | 4.96 | 0.26 | 51.53 | 0.25 | 0.01 | 1.81 | 0.42 | 0.03 | 0.31 | 0.03 | 3.82 |
| 718 | 91.75 | 3.84 | 0.00 | 0.49 | 0.33 | 3.75 | 2.14 | 0.08 | 2.50 | 0.30 | 0.02 | 0.10 | 0.02 | 3.77 |

| | | | | | | | | | | | | | | |
|---|---|---|---|---|---|---|---|---|---|---|---|---|---|---|
| 719 | 0.36 | 0.02 | 0.11 | 9.72 | 0.32 | 123.02 | 0.01 | 0.00 | 2.88 | 0.46 | 0.06 | 0.45 | 0.05 | 2.21 |
| 720 | 68.63 | 2.53 | 0.04 | 5.23 | 0.24 | 50.07 | 0.21 | 0.01 | 1.66 | 0.37 | 0.03 | 0.23 | 0.03 | 3.71 |
| 721 | 71.84 | 2.98 | 0.00 | 0.33 | 0.25 | 2.54 | 0.43 | 0.02 | 2.70 | 0.24 | 0.00 | 0.02 | 0.01 | 2.83 |
| 722 | 64.11 | 4.39 | 0.00 | 0.46 | 0.36 | 2.07 | 3.46 | 0.22 | 2.67 | 0.32 | 0.04 | 0.13 | 0.01 | 2.71 |
| 723 | 60.18 | 3.37 | 0.00 | 0.30 | 0.27 | 2.11 | 1.56 | 0.08 | 2.44 | 0.20 | 0.01 | 0.06 | 0.01 | 2.51 |
| 724 | 54.99 | 5.43 | 0.00 | 0.59 | 0.46 | 2.01 | 1.31 | 0.14 | 3.26 | 0.48 | 0.02 | 0.06 | 0.01 | 2.39 |
| 725 | 58.32 | 3.14 | 0.00 | 0.31 | 0.25 | 1.89 | 0.32 | 0.02 | 1.07 | 0.16 | 0.00 | 0.02 | 0.01 | 2.28 |
| 726 | 51.78 | 3.31 | 0.00 | 0.30 | 0.26 | 1.70 | 0.90 | 0.05 | 0.99 | 0.16 | 0.01 | 0.04 | 0.00 | 2.16 |
| 727 | 43.86 | 3.10 | 0.00 | 0.32 | 0.25 | 1.42 | 1.87 | 0.12 | 0.78 | 0.15 | 0.02 | 0.07 | 0.01 | 1.81 |
| 728 | 30.36 | 2.33 | 0.00 | 0.22 | 0.16 | 1.00 | 1.00 | 0.07 | 0.25 | 0.07 | 0.01 | 0.04 | 0.00 | 1.14 |
| 729 | 75.26 | 4.51 | 0.00 | 0.33 | 0.26 | 2.43 | 0.40 | 0.03 | 1.33 | 0.24 | 0.00 | 0.02 | 0.01 | 3.04 |
| 730 | 84.36 | 4.48 | 0.00 | 0.47 | 0.35 | 2.80 | 1.77 | 0.09 | 1.02 | 0.24 | 0.02 | 0.07 | 0.01 | 3.44 |
| 731 | 42.93 | 2.61 | 0.00 | 0.24 | 0.18 | 1.63 | 0.25 | 0.02 | 0.42 | 0.10 | 0.00 | 0.01 | 0.01 | 1.61 |
| 732 | 89.69 | 4.87 | 0.00 | 0.38 | 0.33 | 3.04 | 5.36 | 0.23 | 5.38 | 0.50 | 0.03 | 0.18 | 0.01 | 3.95 |
| 733 | 44.22 | 2.59 | 0.00 | 0.25 | 0.20 | 1.55 | 1.07 | 0.06 | 1.28 | 0.17 | 0.01 | 0.05 | 0.01 | 1.78 |
| 734 | 36.35 | 3.27 | 0.00 | 0.28 | 0.25 | 1.16 | 0.24 | 0.02 | 0.50 | 0.08 | 0.00 | 0.01 | 0.00 | 1.47 |
